# Fluctuation of ecological niches and geographic range shifts along chile pepper's domestication gradient

**DOI:** 10.1002/ece3.10731

**Published:** 2023-11-28

**Authors:** Natalia E. Martínez‐Ainsworth, Hannah Scheppler, Alejandra Moreno‐Letelier, Vivian Bernau, Michael B. Kantar, Kristin L. Mercer, Lev Jardón‐Barbolla

**Affiliations:** ^1^ Centro de Investigaciones Interdisciplinarias en Ciencias y Humanidades Universidad Nacional Autónoma de México Ciudad de México Mexico; ^2^ Department of Horticulture and Crop Science Ohio State University Columbus Ohio USA; ^3^ Jardín Botánico del Instituto de Biología Universidad Nacional Autónoma de México, Ciudad Universitaria Ciudad de México Mexico; ^4^ Plant Introduction Research Unit, United States Department of Agriculture‐Agricultural Research Service (USDA‐ARS), and Department of Agronomy Iowa State University Ames Iowa USA; ^5^ Department of Tropical Plant and Soil Sciences University of Hawai'i Honolulu Hawaii USA

**Keywords:** center of origin, chile pepper, climate change, climate niche model, domestication gradient, future projection

## Abstract

Domestication is an ongoing well‐described process. However, while many have studied the changes domestication causes in plant genetics, few have explored its impact on the portion of the geographic landscape in which the plants exist. Therefore, the goal of this study was to understand how the process of domestication changed the geographic space suitable for chile pepper (*Capsicum annuum*) in its center of origin (domestication). *C. annuum* is a major crop species globally whose center of domestication, Mexico, has been well‐studied. It provides a unique opportunity to explore the degree to which ranges of different domestication classes diverged and how these ranges might be altered by climate change. To this end, we created ecological niche models for four domestication classes (wild, semiwild, landrace, modern cultivar) based on present climate and future climate scenarios for 2050, 2070, and 2090. Considering present environment, we found substantial overlap in the geographic niches of all the domestication classes. Yet, environmental and geographic aspects of the current ranges did vary among classes. Wild and commercial varieties could grow in desert conditions, while landraces could not. With projections into the future, habitat was lost asymmetrically, with wild, semiwild, and landraces at greater risk of territorial declines than modern cultivars. Further, we identified areas where future suitability overlap between landraces and wilds is expected to be lost. While range expansion is widely associated with domestication, we found little support of a constant niche expansion (either in environmental or geographical space) throughout the domestication gradient in chile peppers in Mexico. Instead, particular domestication transitions resulted in loss, followed by capturing or recapturing environmental or geographic space. The differences in environmental characterization among domestication gradient classes and their future potential range shifts increase the need for conservation efforts to preserve landraces and semiwild genotypes.

## Graphical abstract

We ask how the process of domestication may have modified chile pepper's ecological niche and distribution. We use PCAs and Maxent modeling on different extant domestication levels (wild, semiwild, landrace, and commercial) across *C. annuum*'s domestication center in Mexico. We explore the potential changes in their distribution under future climate scenarios highlighting vulnerable areas per category and areas of potential overlap loss between categories.

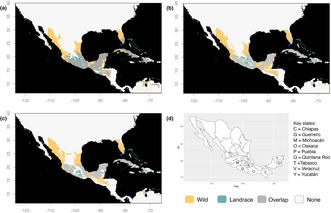

## 1 INTRODUCTION

Domestication drastically transformed plant species, including their interactions with the environment and other organisms, as well as their geographic distributions (Hufford et al., 2019; Purugganan & Fuller, 2009). These evolutionary changes over the course of domestication have occurred due to natural and human‐mediated evolution, partly stimulated by modifications of the growing environment (Eriksson, 2013; Kantar et al., 2017). The results of this evolution can be observed in comparisons of domesticated cultivars and their wild relatives, which help us acquire a deeper understanding of the mechanisms and outcomes of the domestication process (Martín‐Robles et al., 2019; Meyer & Purugganan, 2013; Pace et al., 2015; Swanson‐Wagner et al., 2012). While wild populations are expected to be adapted to abiotic and biotic conditions typical of natural ecosystems, cultivated varieties should reflect, in part, adaptations to human intervention (Milla et al., 2015). Nuanced comparisons between wild and semiwild (i.e., weedy) forms of crop progenitors (e.g., Kane & Rieseberg, 2008) and between landraces and commercial varieties (e.g., Mercer & Perales, 2019) are also helpful to clarify the continuum of differentiation represented within a domestication gradient. Domestication gradients are especially evident in centers of crop origin (i.e., the location where a species underwent domestication) where the continual presence of wild populations can result in continued incorporation of wild individuals into the domestication process (Casas et al., 2007).

It is well understood at a global level that domesticated species are not restricted to the spatial range of their progenitor species. Some domesticated crops such as maize, wheat, and common bean have shown extreme geographic range expansions outside their centers of origin (reviewed in Cortinovis et al., 2020). New environmental conditions encountered by maize have promoted adaptation (Ducrocq et al., 2008; Hufford et al., 2013; Takuno et al., 2015). However, the effect of domestication gradients on geographic distributions within the center of origin (rather than beyond it) is less well‐studied and highly relevant in terms of comprehending the process of adaptation to human cultivation, both original and ongoing.

One way to approach the shifts in geographic distribution over the course of domestication is with ecological niche models (ENM). ENMs seek to describe the multidimensional niche space of a species (Peterson & Soberón, 2012; Soberón & Nakamura, 2009). For every point in a multidimensional niche space, there are one or more corresponding points (Colwell & Rangel, 2009) in physical space (Hutchinson, 1957). Such partial reciprocity has allowed for ENMs to be projected onto physical space to create maps. To this end, species distribution models (SDM) have been successfully used to elucidate the geographic distribution of plants in natural (De Jesús Sánchez González et al., 2018; Khoury et al., 2020) and cultivated systems (Ramirez‐Villegas et al., 2020).

Comparisons between the SDMs of multiple related wild species have shown that new environments may be colonized over evolutionary time. For instance phylogenetically younger wild squash species have been found to transit to wetter environments with respect to older ones (Castellanos‐Morales et al., 2018). While few ENM/SDM studies have compared both wild and domesticated species, exceptions include studies of the Mesoamerican jocote plum (Miller & Knouft, 2006) and teosinte/maize (Hufford et al., 2012). The latter study reports that wild teosintes show stable projections into the past (i.e., no change in distribution compared to current day), whereas the niche projections of primitive maize landraces show evidence of recent expansion (Hufford et al., 2012). When studying wild and domesticated varieties, it is possible that divergence of their niche was propelled by natural and artificial selection. Thus, methods that help visualize environmental clustering such as principal component analyses (PCA) of environmental factors (e.g., Nakazato et al., 2010) and those designed to quantify divergence such as niche equivalency tests and background similarity tests (Warren et al., 2008) are ideal for comparisons of wild and cultivated models.

The use of SDMs can allow for assessment of the future effects of climate change on species distributions (Aguirre‐Liguori et al., 2021; IPCC, 2014). Rapid changes in projected distributions of a taxa might indicate high risk of climate stress that could force their populations to either migrate, adapt to new local conditions, or become extinct (Aitken et al., 2008; Jump & Peñuelas, 2005; Parmesan, 2006). Similar processes may govern crops, though natural gene flow could be complemented by human‐assisted migration (Mercer & Perales, 2010; Pironon et al., 2019). For maize landraces in Mexico, reduction of suitable areas under climate change scenarios was less harsh than for their wild relatives, teosinte and *Tripsacum*. Nevertheless, differential responses were identified per maize race, with vulnerable taxa, as well as new suitable areas, being detected (Ureta et al., 2012). Although distributions of distinct species or taxa within species are expected to shift in different ways, few studies have tackled this issue along a domestication gradient.

Chile pepper (*Capsicum annuum* L.) is an excellent species for studying shifts in environmental niche through the process of its domestication in its center of origin as a domesticated crop. Of the 43 wild chile pepper species, five have been successfully domesticated. *Capsicum annuum* var. *annuum* was domesticated in Mexico (Barboza et al., 2022; Carrizo García et al., 2016; Pickersgill, 1971)—a center of origin hotspot (Vavlilov, 1926) with a long and rich history of chile pepper use (Aguilar‐Rincón et al., 2011). Domesticated chile peppers (*C. annuum* var. *annuum*) differ from wild forms (*C. annuum* var. *glabriusculum*) in a number of traits, such as loss of fruit abscission at maturity; loss of bird dispersal; fewer, larger, and pendant fruits with more synchronous maturation; greater color/shape variability; loss of seed dormancy and nurse plant association; annualization of life cycles; reduction in cross pollination; and reduction in lateral branch number (Luna‐Ruiz et al., 2018; Paran & Van Der Knaap, 2007). Archeological findings date domesticated‐type *C. annuum* use to approximately 5600–6400 years BP, according to colocalization with dated maize and squash macroremains and dated starch fossils from other *Capsicum* species (Long et al., 1989; Mangelsdorf et al., 1965; Perry et al., 2007; Smith, 1967, 1997). Using archeological, geographic, paleobiolinguistic, and genetic evidence, Kraft et al. (2014) concluded that central east Mexico and/or northeastern Mexico were the most likely centers of origin for cultivated *C. annuum*; they could not rule out multiple domestication events, as suggested by others (Aguilar‐Meléndez et al., 2009).

In Mexico, *C. annuum* offers a unique testing ground for the influence of the domestication process on niche evolution. Populations of wild, semiwild (also called let‐standing; e.g., González‐Jara et al., 2011), landraces, and commercial varieties coexist throughout the country, and all four types are actively used by humans. In wild chile peppers, long‐distance dispersal is mediated by birds and establishment is associated with nurse‐plants' understory (Carlo & Tewksbury, 2014). Dispersal of chile pepper landraces is expected to be partly dependent on ancient and modern farmers that have migrated with their crops or exchanged seeds (Orozco‐Ramírez et al., 2016) but see (Aguilar‐Meléndez et al., 2009). Small‐holder management practices influence landrace survival in tropical regions (Perfecto & Vandermeer, 2008). Smallholders tend to cultivate rain‐fed fields, milpas, and backyards and tolerate wild morphotypes (i.e., let‐standing/semiwild types). Landrace plant varieties tend to contain high genetic variability, partly because they are the product of evolutionary interactions with agroecosystems, where natural and human‐mediated selection are at play (Cortinovis et al., 2020). Mexico is the second largest producer globally of chile peppers (second to China; FAO, 2019) and leads the world in exports. Throughout the 2003–2016 period, chile pepper production is estimated to have accounted for 3.2 million annual tons and 3.5% of the national agricultural gross domestic product (GDP), with five varieties leading national production (SAGARPA, 2017). Importantly, many of these commercial varieties are grown in industrial agricultural settings with access to irrigation.

A better understanding of the shift in geographic distributions and environmental space resulting from domestication in Mexican *C. annuum* is lacking. Thus, we employed an environmental niche modeling analysis for the complete *C. annuum* domestication gradient—from wild and semiwild to landrace and commercial cultivars—sampled in Mexico. To this end, we described environmental envelopes per category using PCA analyses and employed a correlative maximum entropy niche modeling method (Maxent) to generate and compare their SDMs. We performed these analyses based on current, as well as future climatic scenarios.

Our objectives were to:

1. Discern differences in the geographic distributions of classes along a domestication gradient.
2. Clarify the environmental factors that climatically differentiate these classes.
3. Compare projected niche suitability of geographic distributions under current climatic conditions, as well as multiple near‐future climate change scenarios, for each class along the domestication gradient.

We hypothesize that there is an expansion of the environmental niche and geographic distribution along the domestication gradient, indicative of human modification of the niche. We also hypothesize that climate change will affect natural populations more severely since they lack the buffering effect derived from human management. These analyses will enhance our ability to understand the ways that the ongoing process of domestication has shaped the ecogeographic qualities of plant species and identify regions at risk for changes in chile pepper distribution as climate continues to shift.

## 2 METHODS

### 2.1 Occurrence point classification system

Occurrence points for *C. annuum* were classified as wild, semiwild, landrace, or commercial. Our wild category included *C. annuum* var. *glabriusculum* (perennial plants with small erect fruits, small flowers) and was defined to occur in natural habitats and exhibit wild phenotypic traits without any sign of chile pepper domestication syndrome. Semiwild (*C. annuum* var. *glabriusculum*, mainly) were characterized as plants morphologically corresponding to *C. annuum* var. *glabriusculum* that occurred in human‐managed spaces such as backyards, small family plots, or *milpa* agroecosystems, where they grow spontaneously as *arvenses* (from Latin word *arvum*: plowed, that is, associated with plowed land) and are tolerated/consumed yet retain a wild‐like phenotype (Aguilar‐Meléndez et al., 2009; Pérez‐Martínez et al., 2022). We acknowledge that, in a few semiwild occurrences, we could not assure the spontaneity of establishment in cultivated/managed land or the completely wild phenotype. Nevertheless, we always directly observed a lack of domestication syndrome in these plants, so maintained them as semiwild in our dataset. Landraces (*C. annuum* var *annuum*, mainly) are identified as such by farmers and communities. They are typically grown with traditional agricultural methods; they tend to be more common in certain cultural regions and associated with specific uses by local communities. Commercials (*C. annuum* var *annuum*) represent cohesive and standardized varieties that are usually grown at large scales through high‐input technology‐dependent agriculture. We recognize that all our domestication classes are at risk of a certain amount of ambiguity/misclassification. Nevertheless, we kept only those data points for which we had sufficient information to classify them.

### 2.2 Data collection and sampling

Our primary source of occurrence points was obtained as the result of numerous field trips throughout Mexico (2013–2015 and 2018–2019) during which 1154 samples, including all four domestication categories were collected. Wild points were subsequently enriched with occurrences reported for Mexico in (Kraft et al., 2014) and (Khoury et al., 2020) as well as public databases: CONABIO (National Commission for the Knowledge and Use of Biodiversity) through SNIB‐REMIB (National Information System on Biodiversity‐World Network of Information on Biodiversity) up to 2014. Additional vouchers from MEXU (UNAM) herbarium accessions were also included. Finally, a curated database of wild *C. annuum* var. *glabriusculum* compiled by CONABIO and analyzed by (Goettsch et al., 2021) was added. For the background tests (see Appendix S1c) the wild class was enriched with Central American data points (Table SA3). Semiwild collection points were complemented with coordinates reported as semiwild from (Aguilar‐Meléndez et al., 2009; González‐Jara et al., 2011). Our semiwild collection raw points encompassed 193 localities that lacked domestication syndrome phenotypes and were thus clearly morphological *C. annuum* var. *glabriusculum* plus 7 localities where individuals displayed traits that were not strictly wild (having slightly elongated fruits or having pendulous fruits). For cultivated varieties, in addition to our collection points, proxies of occurrence points were estimated taking information from agricultural areas within municipalities reported by (SAGARPA, 2018) to produce chile pepper through irrigated and nonirrigated cultivation. The landrace subset included collection landrace points, plus nonirrigated polygon points from SAGARPA. The commercial subset was built with collection commercial points plus both irrigated and nonirrigated polygon points from SAGARPA. To obtain the proxy points, we performed an intersection between the municipality polygons and the agricultural land‐use polygons obtained from INEGI land‐use layers Series VI (2017). This intersection produced smaller polygons where the conditions for crop production are adequate, while excluding areas with natural vegetation. For both irrigated and nonirrigated categories, random points were generated within the retrieved polygons and used as coordinate proxies of true occurrences. Finally, two broad domestication categories were conceived, wild sensu *lato* (wild *sl*), displayed wild‐type phenotypes thus including wild and semiwild subcategories, whereas the cultivated group included landraces and commercial subcategories plus SNIB‐REMIB CONABIO accessions for cultivated chile peppers that could not be classified as either landrace or commercial. The complete data comprised 6160 occurrence points (Figure S1, see data availability table for the breakdown by category, note that these were further pruned and thinned to a set of 5682 occurrences spanning all groupings and categories Table S1).

Occurrence data were processed and formatted for each domestication group. For all data point coordinates, we extracted their corresponding values for 19 bioclimatic variables from layers averaged across 1970–2000, retrieved from WorldClim (Fick & Hijmans, 2017) at 2.5 arc‐min resolution (approximately 5 km). The 17 soil variables were obtained from ISRIC (Hengl et al., 2017) at 2.5 arc‐min, and slope/aspect values were also obtained from WorldClim.

### 2.3 Environmental niche analyses through principal components

**FIGURE 1:**
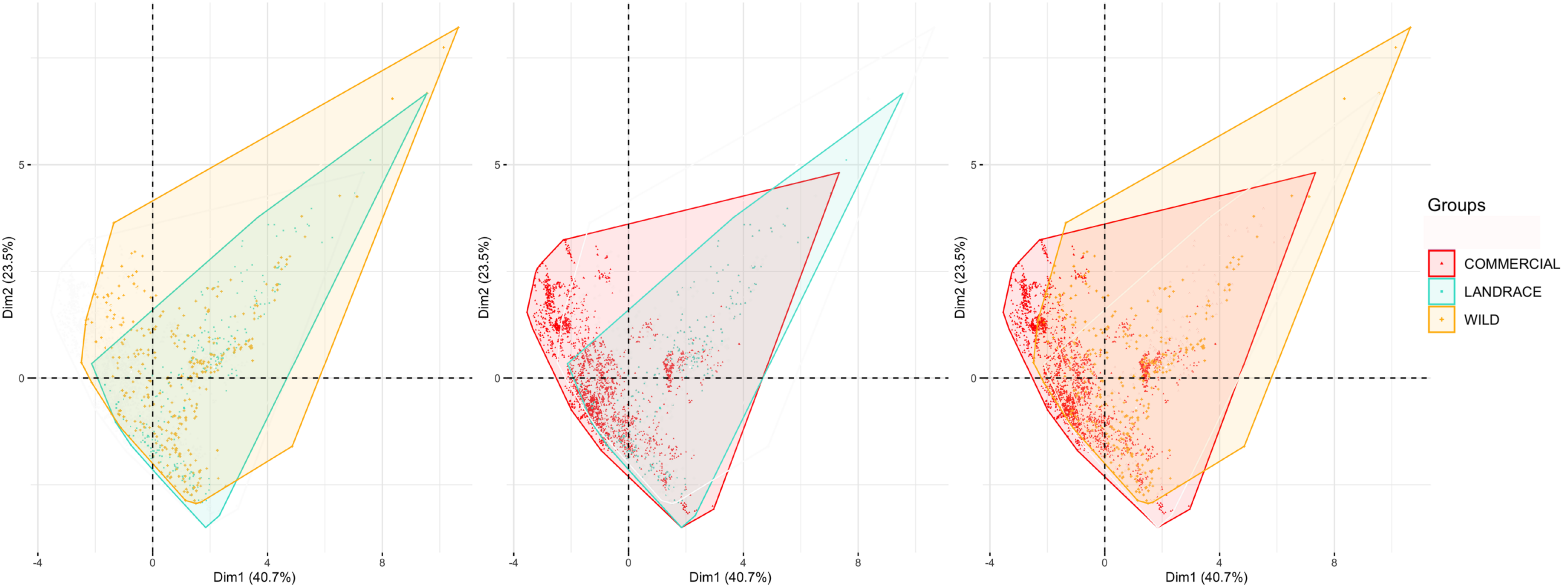
PCA projections on PC1 and PC2 axes for pairwise comparisons of *Capsicum annuum* domestication class in Mexico. Minimum convex polygons are traced for each class. Percent area overlap per domestication class pair for left panel: wild (0.67) landrace (0.88) both (0.76); center panel: landrace (0.87) commercial (0.70) both (0.78); right panel: wild (0.76) commercial (0.80) both (0.78).

**FIGURE 2:**
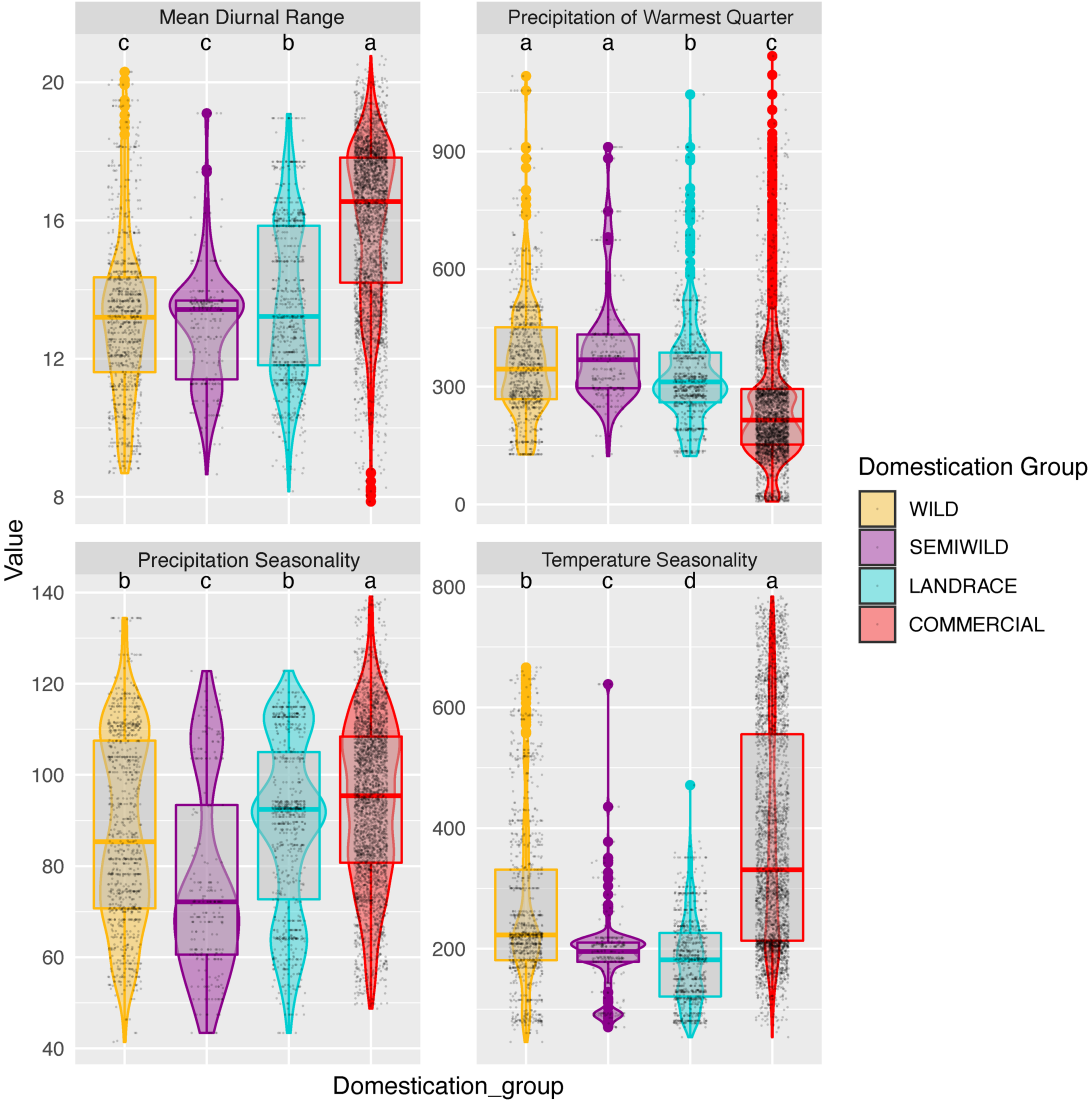
Dispersion of values for four contrasting variables between *Capsicum annuum* domestication classes in Mexico. Boxes within violin plots mark the median and 25/75 percentiles. Significant paired comparisons generated the letter classification labels above each distribution (nonoverlapping CI obtained by 1000 bootstrap resampling). For mean diurnal temperature range and precipitation of the warmest quarter wilds are not significantly different from semiwilds nor was precipitation seasonality between wilds and landraces.

Principal component analysis (PCA) is commonly used to explore environmental niches, as the components of the environment niche are made up of many different variables (Di Cola et al., 2017; Robertson et al., 2001). We selected a subset of variables as described in Appendix S1a. Briefly, we produced variable combinations with low correlations and selected biologically relevant variables according to expert knowledge. We conducted PCAs with “FactoMineR” v.2.3 (Husson et al., 2016) on 9 selected bioclim variables for the whole dataset. These BIOS variables were as follows: 2—Mean diurnal temperature range, 3—Isothermality, 4—Temperature seasonality, 5—Maximum temperature of the warmest month, 9—Mean temperature of the driest quarter, 14—Precipitation of the driest month, 15—Precipitation seasonality, 18—Precipitation of the warmest quarter and 19—Precipitation of the coldest quarter (Table S2). Pairwise comparisons of both the broad and stepwise domestication categories were then performed for the first two principal components. Minimum convex hulls were traced for each category (Figure 1; Figure S2); their areas, the overlap of areas, and the percentage of overlapping area for each category were calculated (Figure S3, Table S3). To account for data point clustering and uneven sampling, we also calculated the percentage of points from each category found in the overlapping area (Table S3). Violin plots were generated for each variable in each domestication category to compare dispersion features, 95% confidence intervals were calculated by resampling from the empirical distribution 1000 times and looking at 2.5% and 97.5% percentiles of the resampled distribution (Figure 2; Figure S4).

### 2.4 Environmental niche modeling

For each domestication category in *C. annuum*, we modeled the environmental niche and performed geographic projections of niche suitability with the maximum entropy algorithm Maxent (Phillips et al., 2006). We selected Maxent because it is ideal for presence‐only datasets and employs a correlative method that has high predictive accuracy (Elith et al., 2011; Merow et al., 2013; Phillips et al., 2006). Environmental layers of the previously selected variables at 2.5 arc‐min resolution were employed for training and testing of the models. They were cropped to a minimum convex polygon, comprising all data points together (thinned at 5 km), and buffered at three degrees. For further details on data preparation and model tuning, see Appendix S1b. The ODMAP (Overview, Data, Model, Assessment and Prediction) reporting protocol (Zurell et al., 2020) is available for this study's distribution models in the Data Availability section. All analyses were performed in R v.4.0.2. (R Foundation for Statistical Computing, 2020) except otherwise described.

MaxEnt runs were performed on thinned datasets with the following specifications. Feature class LQHP was selected for all datasets. Regularization multiplier was set to three for wild, wild *s.l*., semiwild, and landrace datasets and one for commercial and cultivated datasets (Table SA2 and Figure SA3). We decided to use a slightly more stringent regularization multiplier for commercial and cultivated datasets to account for the possibility that human management among the dataset may result in liberal model projections. Clamping was used to account for the artificial sampling barrier we established by including only points within Mexico, a common approach when there are known restrictions on distributions (Stohlgren et al., 2011). We used 10,000 background points and 30% random test points, with 500 maximum iterations. Ten replicates were run using cross validation, and jackknife tests were requested. Logistic output format projections for the custom‐box clipped layers were calculated and the median and SD of the ten replicates were plotted (Figure S5a). Background predictions were logged for downstream true skill statistic (TSS; Allouche et al., 2006) calculations for each mean model (Table SA2). Receiver operating characteristic (ROC) curves and their mean areas under the curve (AUC) were inspected for the ten‐replicate averaged models to ensure consistency among replicates. We compared MaxEnt's output tables for average permutation importance of variables among domestication categories through stacked barplots (Figure S6). Although variable percent contribution explains how much a variable contributed to a model run, permutation importance measures how changing the variable's values affects AUC, and thus its importance for the final model (Songer et al., 2012).

We used “ENMTools” v.1.0.5 (Warren et al., 2010) to calculate three pairwise niche similarity tests between domestication categories: Schoener's *D*, Hellinger's *I* (normalized by Warren) and Spearman rank correlation. These statistics are intended to quantify niche overlap and are calculated from probability distributions assigned to reticulate geographic space. Schoener's *D* assumes that the probability of occurrence in a given cell is related to resource use and thus species local density, whereas Helinger's *I* is free from such assumption (Warren et al., 2008). We assessed spatial bias of our different sampling strategies by generating a spatial bias file (Fourcade et al., 2014) buffered at one degree for each dataset and cropped each environmental raster accordingly. MaxEnt models using these files produced overprojections, indicating decently spread sampling effort throughout study areas. To further complement the approach, and in view of our sampling biases among datasets, we decided to measure ecological niche overlap between wild‐landrace and wild‐cultivated pairs in environmental space (Broennimann et al., 2012), taking random distributions of variables from the predicted distribution to calculate *D* and *I*. To better compare the resulting MaxEnt suitability matrices on geographic space, we transformed each median map into binary color codes by calculating a specific threshold for each dataset. See Appendix S1d for details.

### 2.5 Niche projection for future climate scenarios

Projecting the niche of each domestication category under future climate scenarios allowed us to determine how they might be differently affected and how potential geographical shifts could modify the amount and location of overlapping regions among categories.

To do so, each of the ten replicate models obtained per dataset was independently projected onto future climates (Fick & Hijmans, 2017) CMIP6 (Coordinated Model Intercomparison Project Phase 6; Eyring et al., 2016) using terminal commands (using the same layers with the same extent and resolution as present‐day models; Appendix S1b). The future layers included scenario averages for temporal intervals of years 2041–2060, 2061–2080 and 2081–2100, under two shared socioeconomic pathways (SSPs): 2 (“middle of the road”) with representative concentration pathway (RCP) of 4.5, and 5 (“fossil‐fueled development”) with RCP of 8.5. For each of the afore‐mentioned combinations, the eight general circulation models (GCMs) that were available for CMIP6 were run (BCC‐CSM2‐MR, CNRM‐CM6‐1, CNRM‐ESM2‐1, CanESM5, IPSL‐CM6A‐LR, MIROC‐ES2L, MIROC6, and MRI‐ESM2‐0). GCMs simulate the relationships between atmosphere, oceans, land, and ice, to try to project how CO_2_ would impact temperature and how temperature affects the other climatic variables (precipitation, cloud cover, wind, etc.). Mean, median, and standard deviation ASCII maps were obtained for each set of ten replicate future projections per configuration in R (data not shown).

Binary maps were obtained for projections of niche suitability under future climate scenarios to allow an easier comparison of differences and overlaps. Thresholds on future projections per domestication category were obtained from the present time models of each dataset and applied to each year‐SSP‐GCM model median map. Within each domestication category, pixel overlap metrics (loss, gain, and shift) were calculated for each future projection as compared to their present projections, for each GCM, as well as their intersection. In addition, equivalent calculations were obtained for pairwise overlap of the domestication classes (Table S4). To better visualize future scenarios results, we used the intersection of the eight GCMs and compared this to present‐day projections and future overlaps between domestication categories as described in Appendix S1d (see Figure SA4 for the sum and intersection of all GCMs per year‐SSP scenario). In this stringent way, from the threshold‐retained pixels for each model, we took only the pixels that were supported by all eight GCMs for a given year‐SSP scenario.

ENM sources of uncertainty are manifold, and studies reviewing niche model projections' transferability in time identify (1) the type of future scenario selected and (2) the migratory potential of the organism among the main issues (Sequeira et al., 2018; Yates et al., 2018). Some urge caution when nonanalog combinations of conditions are expected (Fitzpatrick & Hargrove, 2009; Zurell et al., 2012). For this reason, Qiao et al. (2019) suggest that environmental similarity be calculated to assess uncertainty in model transferability. Thus, we calculated multivariate environmental similarity surfaces (MESS; Elith et al., 2011) between our present and future projections and their layers (Appendix S1e, Figures S9 and S10).

## 3 RESULTS

### 3.1 Environmental niche analyses through principal components

We built a final Mexican chile pepper database comprising 6160 occurrence points spatially thinned to comprise 5682 coordinates, these were further classified and clustered into 302 wild, 86 semiwild, 277 landrace, 2178 commercial, 367 wild‐sl, and 2472 cultivated groups of occurrences (Table S1). In the PCA analyses on the four fine‐grained categories (wilds, semiwild, landraces, and commercials), first and second principal components (PC1 and PC2) accounted for 40.7% and 23.5% of the variance respectively (Figure 1; Figure S2). Concentrating on the quadrants where pairwise minimum convex polygons mainly differed, we found that PC1 loadings displayed strong negative correlation with the mean diurnal range and temperature seasonality whereas PC2 showed high positive correlation with temperature seasonality and precipitation of the coldest quarter (Table S2). Landrace PCA polygon area was mostly contained within the wild polygon (Figure 1‐left panel); wild and landrace polygons overlapped by 76%, with 88% of landrace area shared with wild and 67% of the wild area shared with landraces (rounded figures). Hull point density overlap estimates were 99 and 89% for landrace and wilds, respectively (Table S3). The wild data points that fell outside the landrace polygon corresponded to a few hot and dry northern regions (Figure S3a). For the landrace and commercial pair, we observed that very few landrace data points from very humid regions were not encompassed by the commercial variety hull envelope. Areas exclusive to the commercials expanded in the negative PC1 and positive PC2 quadrant, corresponding to some of the country's northern desert regions (landrace and commercial polygons overlapped by 77% and their hull point density overlapped by 72%, Figure 1‐center panel, Figure S3b). When comparing the extremes of the domestication gradient (wilds vs commercials), we found that commercial varieties have greatly recolonized the hot and dry conditions suitable for wilds (but not landraces), including those found in the inland northwestern deserts (Figure 1‐right panel; Figure S3c).

In exploring the environmental space encompassed in current distributions, we identified significant pairwise comparisons among domestication classes of the environmental variables that contrasted most prominently for the occurrence points (95% CI, Figure SA3). For example, the average precipitation of the warmest quarter gradually diminished from wild and semiwild to landraces to commercials (Figure 2). Mean diurnal range was greater for commercials than landraces, which were in turn, greater than the wild and semiwild classes. Moreover, precipitation seasonality increased from semiwilds to wilds and landraces to commercials (however, wilds and landraces CI overlapped for precipitation seasonality) (Figure 2). Landraces were found in the lowest ranges of temperature seasonality, while commercials showed considerable dispersion with the highest median value (Figure 2).

### 3.2 Environmental niche modeling

**FIGURE 3:**
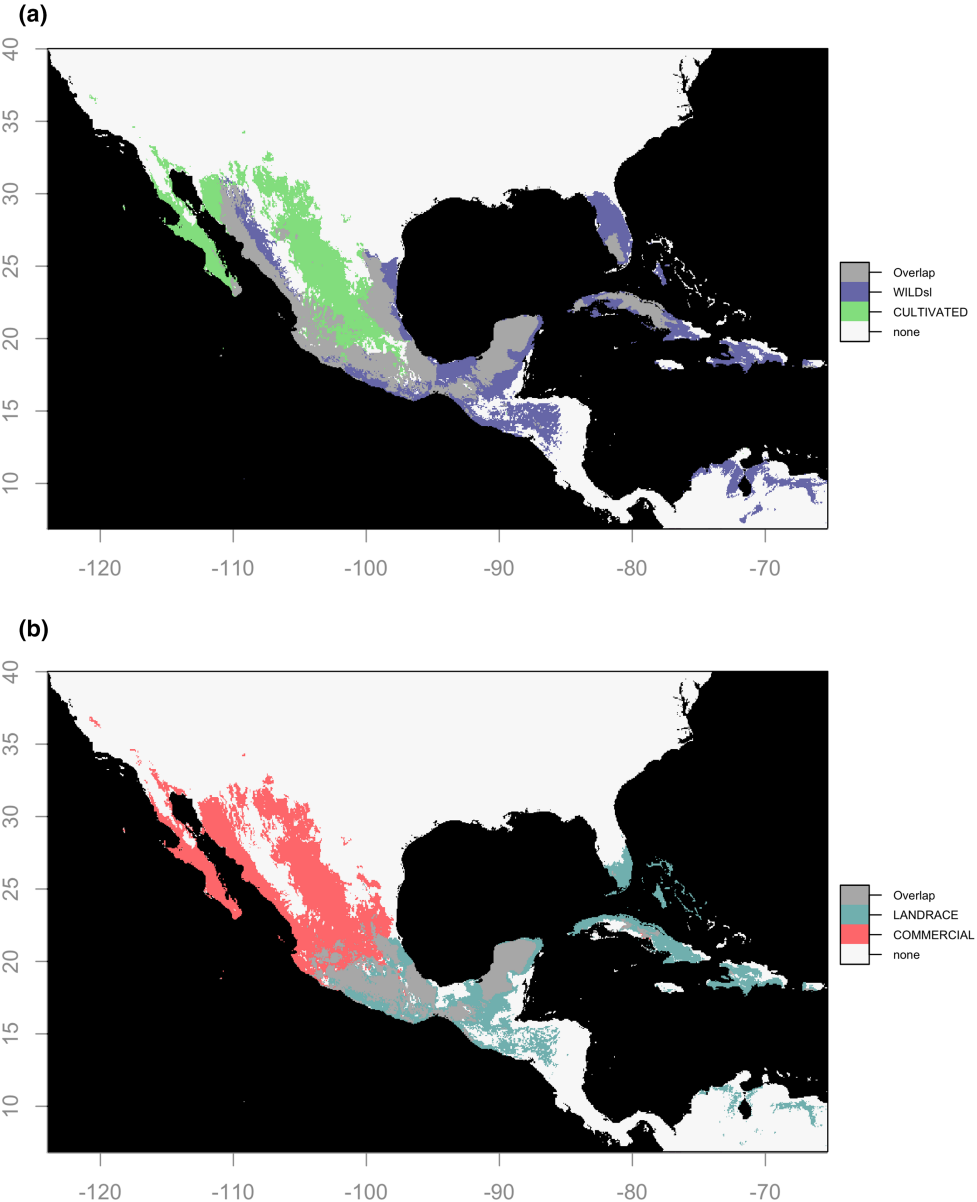
Overlap of suitability maps for *Capsicum annuum* niche projections in a Mexico‐centered rectangle. Median from 10 replicates of logistic *maxent* outputs. Colored areas correspond to pixels above the 10th percentile threshold of training points for each domestication class. Gray areas show projection overlap between niches. (a) Wild sensu *lato* (wilds + semiwilds) and cultivated (landraces + commercial varieties + varieties that are cultivated but could not be assigned to landrace or commercial subgroupings). (b) Overlap of suitability maps for *C. annuum* landrace and commercial niche projections.

**FIGURE 4:**
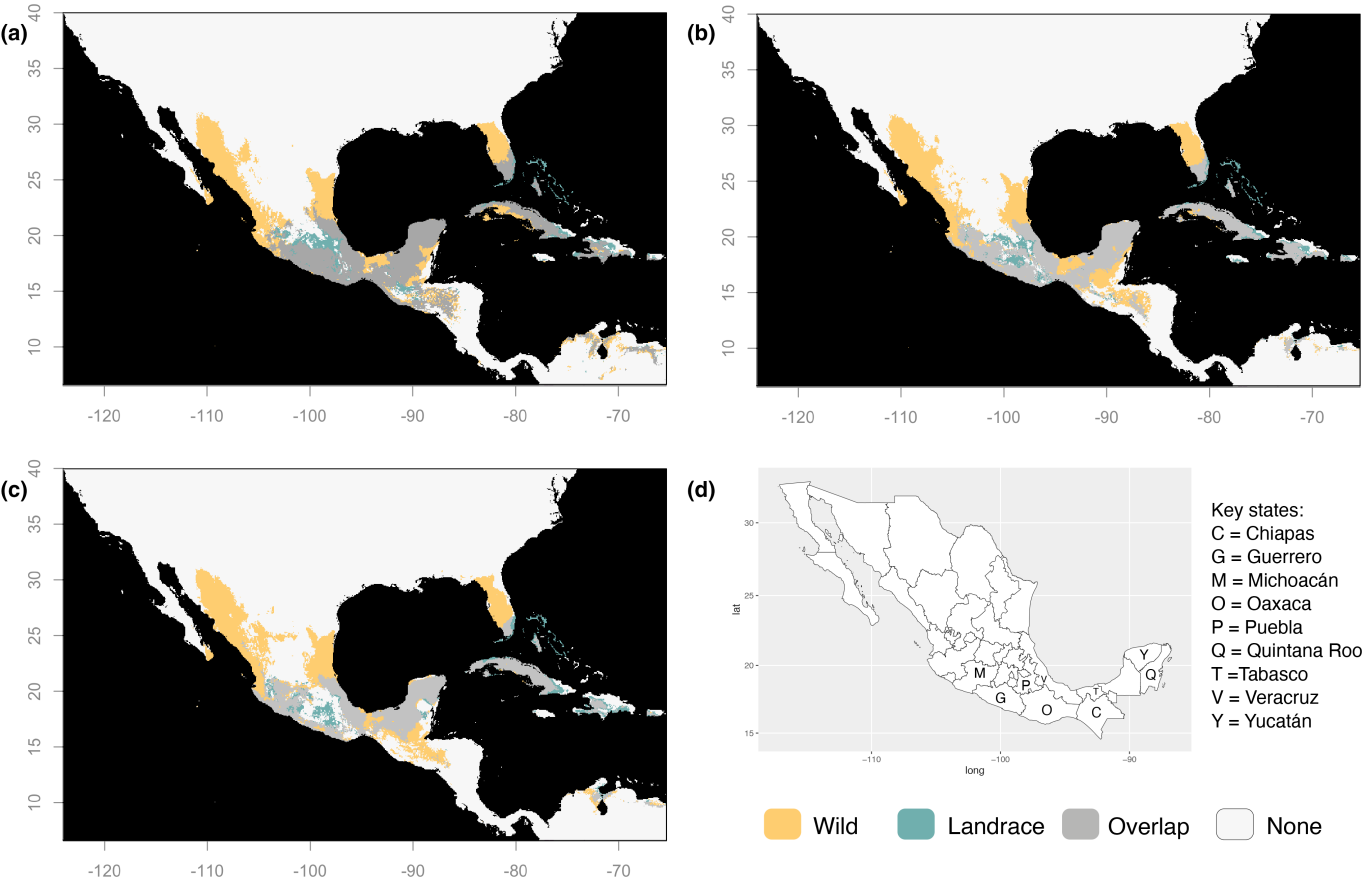
Overlap of suitability maps for *Capsicum annuum* wild and landrace niche projections in a Mexico‐centered rectangle for present conditions (a), future 2090 conditions under SSP 2–4.5 (b) and future 2090 conditions under SSP 5–8.5 (c). Median from 10 replicates of logistic *maxent* outputs. Colored areas correspond to pixels above the 10th percentile threshold of training points for each domestication class. Gray areas show projection overlap between niches. For each domestication class, future area was obtained as the shared area among all eight GCM models, with the same 10th percentile threshold from training points of the present niche model used to plot binary maps each future projection and calculate overlap among GCMs. Mexico political map with states referred to in the discussion are labeled with their initial (d).

From the maxent model projected overlap in the current geographic distribution of our broad wild‐sl and cultivated categories, it was evident that domestication categories differed geographically, although there was a 52.9% overlap between wild‐sl and cultivated distributions (Figure 3a). Suitable areas for cultivated chiles extended into dry climate and desert regions, while wild‐sl suitability could be found in highly humid regions, as well as along most coasts. At fine grain domestication categories, landrace and wild suitable areas mostly overlapped in central to southeastern Mexico; however, there were exceptions in the swamp‐marsh sections of Tabasco state and the south‐eastern section of Quintana Roo state, where only wilds found suitable conditions (Figure 4a). Noteworthy were two regions exclusively suitable for landraces and not wilds: the Trans‐Mexican Volcanic Belt (TMVB) and Guatemalan Sierras, both distinguished by their high altitude. Landraces distributions were not larger than those of wilds (Table SA4). When comparing landrace and commercial classes, commercials found high suitability in northern desert regions where landraces lacked suitable areas (Figure 3b). Some areas in southern Mexico were suitable for landraces, but not commercials, such as the tropical coasts of the states of Guerrero, Oaxaca, and Quintana Roo, as well as some humid regions of the states of Chiapas and Tabasco. In these same areas, the distribution of landraces tended to overlap with wilds (Figure 4a).

When we analyzed variable permutation importance, we observed that wild and wild‐sl geographic distributions were strongly predicted by the precipitation of the warmest quarter, whereas semiwilds and landraces were more influenced by temperature seasonality. The importance of temperature seasonality was then greatly reduced for the commercial and cultivated categories (Figure S6). For these four domestication categories, the described variables also showed a high contribution to each model through the jackknife test, as did temperature seasonality for wilds and wild‐sl (Table S5). Precipitation of the driest month showed considerable permutation importance for landraces. All of these variables had strong contributions and loadings in the PCA (Table S2), where in addition, mean diurnal range and precipitation of the coldest quarter also figured.

Pairwise niche similarity tests indicated that among the four fine grained category pairs, landrace and semiwild were the most similar (*D* = 0.79, *I* = 0.95) and commercial and landrace were the least similar (*D* = 0.49, *I* = 0.75; Table S6). Landraces and wilds displayed intermediate values of similarity (*D* = 0.68, *I* = 0.89) that were higher than the commercials and wilds comparison. Further analyses through background tests on wild‐landrace and wild‐commercial pairs clarified that the landrace environmental niche was indeed nested within the wild niche but not vice versa, and that the commercial and wild niches are less similar than random expectations, thus significantly divergent (Figure S7).

### 3.3 Niche projection for future climate scenarios

**FIGURE 5:**
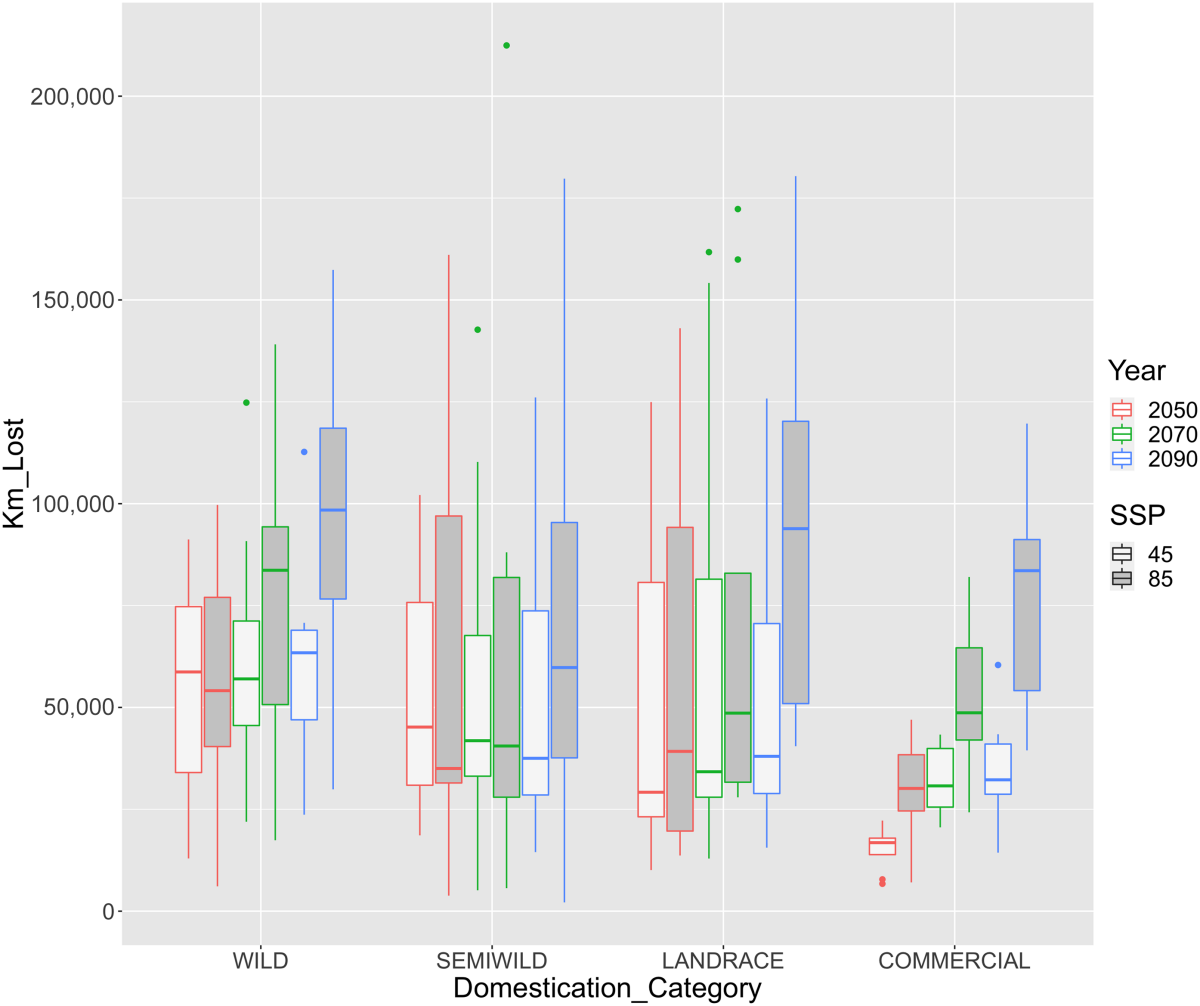
*Capsicum annuum* lost area by future niche projection within each domestication class with respect to their niche projections at present. Boxplots represent dispersion in the amount of area lost according to each of the eight general circulation models employed. Boxes are outlined with color codes for future projection years and filled according to SSP 2–4.5 and SSP 5–8.5 (rcp 45 and 85, respectively). The same 10th percentile threshold of training points at present was used to plot binary maps for each domestication classes' future projections and calculate overlap.

To elucidate future conservation needs and the potential for maintaining the evolutionary processes ongoing in chile pepper, we examined the niche projections of each domestication category under a spectrum of future climate scenarios (i.e., different mitigation strategies and GCMs). For each domestication class, we visualized the amount of lost area for all year and SSP combinations (Figure 5; Figure S8, Table S7). There was substantial dispersion across the eight GCMs for each class and year combination. In general, we observed that the amount of original area lost increased over time and at a faster rate for SSP 5–8.5, for all domestication categories, except for semiwild (Figure 5). For a given year, results by SSP scenario showed variation in area loss among GCMs, yet in general, the wild category was projected to experience the highest area loss (Figure 5).

**FIGURE 6:**
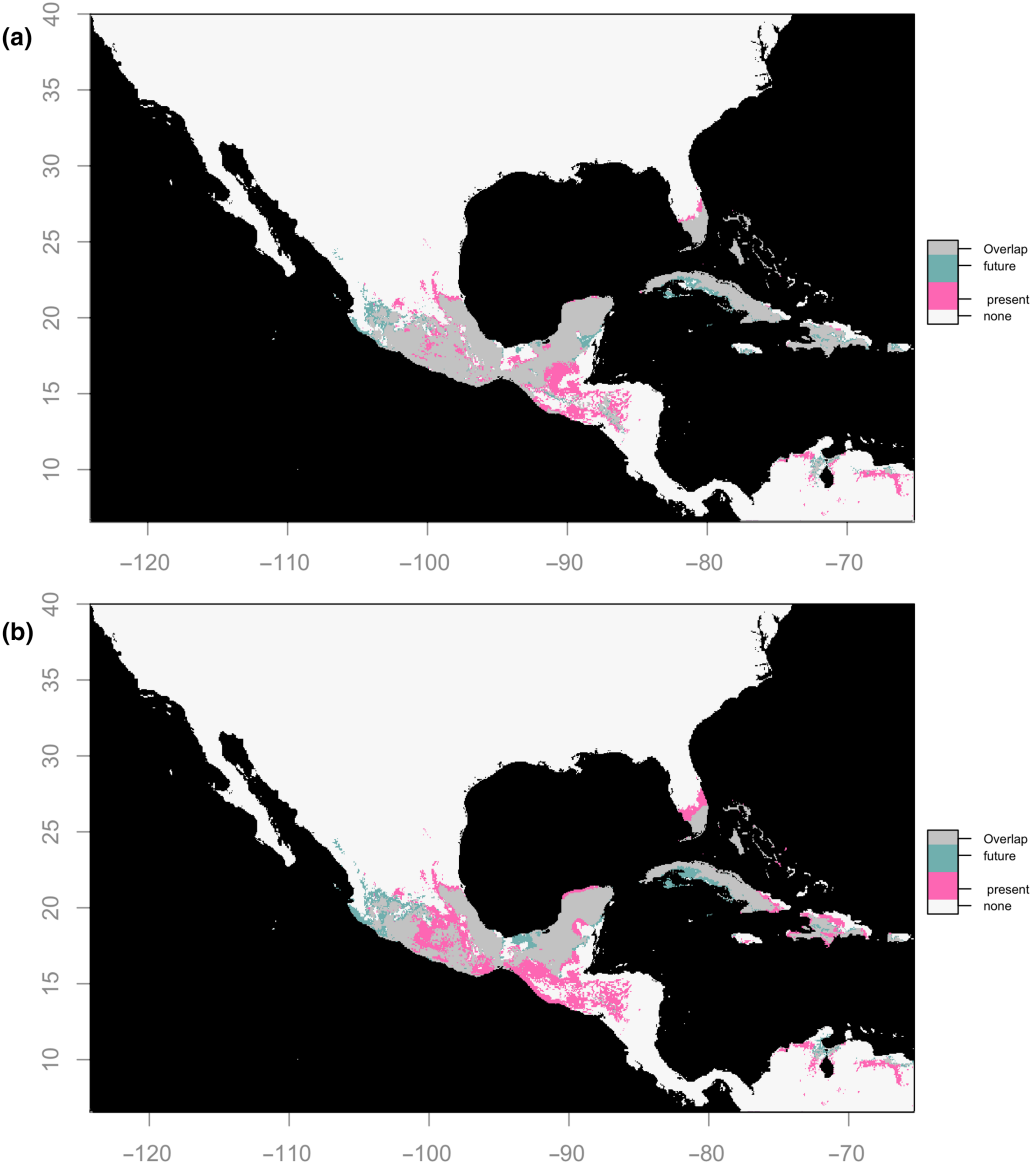
Overlap between present and future projections (year 2090) in a Mexico‐centered rectangle for *C. annuum* landrace niche under (a) SSP 2–4.5 and (b) SSP 5–8.5. Future area was obtained as the shared area among all eight GCM models, with the same 10th percentile threshold from training points of the present niche model used to plot binary maps each future projection and observe overlap among GCMs.

To identify geographic areas under potential climate change threat for each domestication category, we used the intersection of all eight GCMs in each year and SSP scenario combination and visualized the geographic overlap of present projections with future ones. Some areas typically suitable for landraces are expected to no longer be suitable (i.e., are at risk of being lost) by the year 2090 (Figure 6). Under SSP 2–4.5, landraces are at risk in Guatemalan regions (Figure 6a; proliferation of pink area); under SSP 5–8.5 they become at risk at high elevations—both in the Trans‐Mexican Volcanic Belt (TMVB) and Guatemalan sierras—as well as along the low elevation area along the Pacific coast of the Isthmus of Tehuantepec (Figures 6a,b; proliferation of pink area).

We took notice of potentially vulnerable landraces that we had observed as present in these areas at risk of becoming unsuitable, i.e.,“lost” areas (Table S8). Landraces at risk included the following: in Campeche, Maax‐ik from backyards and Tabaquero from milpas; in Chiapas, Chile de Árbol, Chilito, and Miraparriba backyard landraces in Chiapas; and in Oaxaca, Bolita, Paradito, Chilgole, Guajillo, Jalapeño, Mirasol, Pijita, and Tusta backyard landraces, as well as milpa‐grown Huacle, Taviche, and Tusta and plantation‐grown Costeño Rojo and Chile de Agua. Maax‐ik is a special case because it can be found as semiwild and wild too, hopefully buffering the threat on this landrace. Additionally, locally unique landraces at risk included Querétaro's backyard Chile Criollo, Tabasco's forest‐thriving Garbanzo and the Yucatán Peninsula milpa's Chile Dulce and Xcatic.

When assessing the change in area of overlap between wilds and landraces at present vs future (e.g., 2090) projections, we found that the sierras of Guerrero and Oaxaca were particularly prone to loss of overlap due to reductions in both wild and landrace suitability (Figure 4; loss of gray area). Overlap in Guatemala was also projected to decline in the future due only to reductions in landrace suitability only.

We worked on temporal transfer of model projections, so it was important for us to assess if the resulting vulnerable areas corresponded to non‐analog climate combinations. To this end, we contrasted our maps of future area loss and future overlap loss among classes with MESS maps generated for each year‐SSP‐GCM combination (Figure S9; novel combinations: red shading). For the MESS projections to 2090, novel climate combinations were scarce (SSP 5–8.5) or generally absent (SSP 2–4.5) in the regions with landrace area loss or overlap loss between landraces and wilds. When MESS was estimated for landrace niche model for 2090 only, novel combinations included GCM models IPSL‐CM6A‐LR and MRI‐ESM2‐0, for SSP 5–8.5, and only MRI‐ESM2‐0 for SSP 2–4.5 (Figure S10b). The same was true for wilds with the addition of two small patches in Tabasco and Michoacán states for 5–8.5 BCC‐CSM2‐MR.

## 4 DISCUSSION

With our extensive sampling of wild, semiwild, landrace, and commercial chile pepper in the center of crop origin for *C. annuum*, we clarified the current climate envelope and geographic distribution of each domestication class. Wild and commercially cultivated plants were found within diverse environments including hot and dry conditions. By contrast, landraces were mostly distributed within the environmental space of the wilds, excluding drier environments, but occupying humid regions not available to commercials. Landraces were also found at higher elevations where wilds were notably excluded. Thus, although considerable overlap exists between the environmental envelopes of the domestication classes, we found that domestication, improvement, and management have resulted in a distinct utilization of the environment. We found some environmental variables to gradually increase or decrease along the domestication gradient. Precipitation of the warmest quarter diminished with domestication and improvement and mean diurnal range increased. By contrast, temperature seasonality was highest for wild and commercial types and lower for intermediate domestication classes. Furthermore, our niche model projections provided clues as to how niche evolution changed the geographic space suitable for chile pepper in its center of origin. We found a contraction domestication and spread and presumably a secondary expansion or shift of the chile pepper niche with improvement and more intensive management. Finally, future projections suggest that the greatest shrinkage of current area should occur where wilds grow, while landraces may be most vulnerable in high elevation environments. As a consequence, some regions of overlap between wilds and landraces may be lost in the future, affecting future gene flow and evolution in *C. annuum*.

### 4.1 Human management has shaped the ecological niche of cultivated pepper

Landraces appeared to require a certain degree of climatic stability (lowest temperature seasonality and highest isothermality values; Figure 2; Figure SA3). Commercials, by contrast, grow where exposed to high precipitation seasonality. Precipitation of the driest month had high permutation importance for landraces, presumably attributable to the fact that most landrace points came from rain‐fed management regimes. Thus, although literature has suggested that small‐holder practices—especially polyculture—may stabilize the environment crops experience (Altieri et al., 2015), we suggest that management practices typical of landraces may be less able to buffer certain environmental conditions than those of high input agriculture. The presence of commercial chile peppers in dry areas on the suitability maps (Figure 3b) and their low PCA values for the precipitation of the warmest quarter (Figure 2) could be explained by the use of artificial irrigation. Although we cannot definitively deduce that the commercial niche “evolved” per se since we did not include irrigation as an explanatory variable and lack genetic data, it is worth remembering that we partially accounted for ways that management may mitigate environmental constraints by increasing the strictness of commercial and cultivated models using a regularization multiplier of a third the size of that employed for the other domestication classes.

Commercial varieties and wilds constitute extreme ends of the domestication gradient. Thus, we expected that their environmental niche models and distributions would show the strongest divergence. By contrast, our niche similarity measures were lowest for the landrace‐commercial pair (*D* = 0.49, *I* = 0.75, Table S6), both of which are cultivated types. This surprising result may be due to divergence in their histories. Landraces are the product of a long cultural process of domestication, spread, and diversification within a labor‐intensive management system, while commercials, which were bred from landraces, experienced recent formal breeding for success within high input, technologically advanced agricultural systems. It therefore seems possible that the nature of the divergence between wilds and landraces may have ultimately made this crop further amenable to other production systems (i.e., intensive commercial management), a change which was ultimately propelled with commercial breeding efforts. Further, the domestication process allowed for the expansion of the commercial niche by reclaiming areas typical of wilds, but not exploited by landraces (presumably by means of artificial irrigation) (Figure 1; Figure S5). Nevertheless, our background tests indicated that, although commercials and wilds both occupy dry areas, their suitable conditions are in fact highly differentiated, while landraces were effectively nested within wilds. Moreover, the genetic evolution we assume to be relevant for the wild‐landrace transition may or may not be echoed in the landrace‐commercial transition. For commercials, it is possible that recent artificial irrigation could explain a fair part of their suitability in low precipitation environments and the resulting geographic distribution in dry areas.

Some species, such as maize and plum, show a gradual expansion of their environmental niche and its geographic projections along their domestication gradient (Calfee et al., 2021; Locqueville et al., 2022; Miller & Knouft, 2006; Purugganan, 2022). By contrast, our results indicate that chile pepper domestication may have involved environmental subsampling of the wild climatic and geographic distributions such that landrace distribution is now mostly nested within the wild one. Commercials, however, expanded beyond landraces and partially returned to wild areas. Had we run our analyses on a worldwide sample of *C. annuum*, we might have expected that the expansion of commercial varieties would be more extreme as can be seen for sunflower (Mehrabi et al., 2019). For instance, a study on the global expansion history of pepper using genetic and passport data indicate that Africa has been a reservoir of ancient Mesoamerican varieties, Eastern Europe holds unique *C. annuum* diversity and East Asia has possibly contributed an additional secondary diversification center (Tripodi et al., 2021). Diffuse or multiple domestication events may complicate the gradual expansion hypothesis. Studies to date have not compellingly concluded a single chile pepper domestication event (Aguilar‐Meléndez et al., 2009; Kraft et al., 2014) and wild chile peppers are distributed throughout the country (Khoury et al., 2020). Thus, the lack of environmental and geographical expansion along the domestication gradient of *C. annuum* may be due to the unresolved complexity and possibly multiple domestication origin.

### 4.2 Domestication allowed for expansion into higher elevations

Traditional agriculture, while greatly responsible for landrace preservation and ongoing evolution (Altieri et al., 2015; Jardón Barbolla & Benítez Keinrad, 2016), does not seem to have drastically augmented the amount of area suitable for chile pepper landrace production when compared to wilds. Landrace geographic projections overlap with those of wilds in highly humid regions where initial domestication likely occurred. Nevertheless, wild and landrace suitabilities do not overlap in the highlands (e.g., the TMVB and Guatemalan sierras), and ecogeographic region is suitable only for landraces (Figure 4a). In other emblematic crops, the acquisition of additional adaptations following their initial domestication was a fundamental for colonization of higher elevations (Calfee et al., 2021). In maize, this was achieved through adaptive introgression from their high elevation‐dwelling wild relative (Hufford et al., 2013). In chile pepper, besides a resequencing study suggesting that phylogenetically old cultivars may carry adaptive material for high elevations (Cao et al., 2022), this highland migration and its required genetic adaptations are unexplored. However, we propose that the ecogeographic shift into higher elevations was a key event in chile pepper domestication. It is unlikely that the two wild pepper species found at high elevations, *C. lanceolatum* and *C. rhomboideum* (found in the Guatemala and Chiapas highlands), contributed necessary adaptations through introgression since each has a base chromosome number of *x* = 13, a characteristic exclusive to the Andean clade to which they belong, not *x* = 12 as in *C. annuum*, which belongs to the Annuum clade (Carrizo García et al., 2022; Scaldaferro et al., 2013; Scaldaferro & Barboza, 2023; Tong & Bosland, 2003). While the Andean clade's chromosomal characteristic may relate to adaptation to high elevations (Scaldaferro & Barboza, 2023), the phylogenetic position of these Andean species (Carrizo García et al., 2022) indicates that they diverged from all other *Capsicum* clades in the middle Miocene. Thus, their distant relation to *C. annuum* and further hinders their playing a role in *C. annuum* elevational expansion.

While commercial chile pepper showed considerable geographic expansion relative to other domestication classes, it remained excluded from certain highly humid lowland areas (e.g., Lower Huasteca in the state of Veracruz, the Riviera Maya in the state of Quintana Roo, and the state of Chiapas) where wild and landrace suitable areas overlap (Figures 3b and 4a). These regions are notably dominated by subsistence production systems (De Clerck & Negreros‐Castillo, 2000) farmed by small holders who have maintained genetic diversity in landraces (Altieri et al., 2015; Dobler‐Morales et al., 2020). There may be both biological and social reasons for industrial production being excluded from these areas. These humid regions are prone to pests such as the silverleaf whitefly (*Bemisia tabaci*, Aleyrodidae), which shows some resistance to the natural chemical defenses of cultivated peppers (Ballina‐Gomez et al., 2013; Latournerie‐Moreno et al., 2015), and fungal pathogens often associated with monocultures in humid zones. These regions may also lack the social infrastructure or land requirements to support industrial farming operations, limiting their expansion.

### 4.3 Climate change may threaten unique landraces and reduce the adaptive capacity of crops

Under “fossil‐fueled development” 5–8.5 SSP scenario with no CO_2_ emission mitigation policies (using the highest Representative Concentration Pathway‐RCP8.5), all fine‐grained domestication categories, except semiwild, saw a reduction in the amount of suitable area that overlapped present‐day coverage as climate change progressed over time. While commercial and wild distributions were both large under current conditions, there was a larger shift in the future suitable area of the wilds (Figure S8). Yet we are skeptical that such strong migrations will in fact occur for two reasons. For wilds, although avian dispersal could potentially aid the migration, it is unclear that the distance and rates will suffice, given the speed of climate change and the reliance on nurse shrubs for their establishment in available natural settings. For commercial varieties, we do not assume their projected future distributions to be realistic in areas that lack irrigation. Thus, their migration would necessitate changes in the farming systems, which would require nationwide coordination and public policies. For these reasons, we focus the rest of our discussion on the results of future scenarios on areas at risk.

Landraces often are locally adapted to specific environmental and management conditions and are conserved in situ (Galluzzi et al., 2010; Mercer & Perales, 2010) in part because farmers value their substantial adaptive genetic diversity (Chen et al., 2017). Landraces can act as genetic reservoirs, providing opportunities for adaptive gene flow (Mercer & Perales, 2010), as described with improved cultivars in *Cucurbita* (Martínez‐González et al., 2021). For *C. annuum* landraces in 2090, we found a strong impact of suitable area loss in Guatemala for both 2–4.5 and 5–8.5 scenarios. Additionally, the nonmitigated scenario (SSP = 5–8.5) also resulted in an important rise in landrace vulnerability for highlands and the Isthmus Pacific coast (Figure 6). Coastal areas of Veracruz, Guerrero, parts of Oaxaca, as well as Quintana Roo and Tabasco are all expected to maintain suitability for landraces. However, a portion of these (particularly the last two) might become inhospitable in the future due to rising sea levels. ENM projections in future climate scenarios benefit from considering the effects of unequal shifts of biotic interaction partners (e.g., Carrasco et al., 2020; Mehrabi et al., 2019). Adding biotic partner layers could deepen our results.

Landraces currently grown in the areas that we predicted will cease to be suitable, might require special attention for in situ conservation in the future. We found 21 such landraces, 12 of which were found in the state of Oaxaca (Table S8). The strong representation of these in the state of Oaxaca may be due to the location of the expected shift and/or reflect denser sampling of greater diversity in that state. Fortunately, vulnerable landraces in each state were accompanied by alternative populations of the same landrace that our results show are expected to be maintained under climate change. Among vulnerable landraces detected, Chile Criollo, Tabaquero, Garbanzo, and Costeño Rojo were the ones expected to be under threat in the near future (starting in 2050). The first three have restricted distributions, whereas Costeño Rojo is popular in the larger Costa Chica region (Oaxaca and Guerrero coast) where it is grown in milpas or plantations and used by Mixtec and Afro‐descendant peoples (Muñoz‐Zurita, 2015). The wild‐resembling Garbanzo found in forests might be a valuable gene flow bridge between wild and cultivated germplasm (Castañón‐Nájera et al., 2008). The historical and cultural value of some of these landraces is linked with native peoples, such as Oaxaca's Taviche which is endemic to the Mixtepec region and Tusta's association with the Zapotec and Chatino peoples from the Loixcha region (Sánchez‐Cortés, 2021; Santiago‐Luna et al., 2015). This highlights the importance of in situ conservation strategies within relevant historical cultural context.

Our models suggest that climate change can be expected to diminish the geographical overlap between wild and cultivated peppers. We found that wilds may be lost in the mountains of Guerrero and Oaxaca where they currently overlap landraces, whereas landraces may withdraw from areas in Guatemala where they is currently overlap wilds; both wilds and landraces may retract from Puebla and southern Chiapas (Figure 4). These losses of wild‐landrace overlap may have consequences for the evolutionary processes ongoing in this center of origin. As discussed above, many crops have been reported to evolve adaptations to new climates through wild to landrace introgression (reviewed in Janzen et al., 2019), a process that may be hampered if wild and crop geographical overlap is strongly diminished (Zhang et al., 2014). In chile pepper, cultivated and wild forms of *C. annuum* can easily hybridize (Eshbaugh, 2012; Pérez‐Martínez et al., 2022). Often, wild chile peppers establish in milpas and backyards, providing opportunities for wild‐to‐landrace gene flow (González‐Jara et al., 2011; Guzmán et al., 2005; Pérez‐Martínez et al., 2022). Small‐holder plots that steward this evolutionary process in situ should be encouraged in regions where wild and landrace distributions overlap.

## AUTHOR CONTRIBUTIONS

**Natalia E. Martínez‐Ainsworth:** Conceptualization (equal); formal analysis (lead); writing – original draft (lead); writing – review and editing (equal). **Hannah Scheppler:** Formal analysis (supporting); writing – review and editing (equal). **Alejandra Moreno‐Letelier:** Conceptualization (equal); formal analysis (equal); writing – review and editing (supporting). **Vivian Bernau:** Conceptualization (equal); writing – review and editing (equal). **Michael B. Kantar:** Conceptualization (lead); writing – review and editing (equal). **Kristin L. Mercer:** Conceptualization (equal); funding acquisition (equal); writing – review and editing (equal). **Lev Jardón‐Barbolla:** Conceptualization (equal); funding acquisition (equal); writing – review and editing (equal).

## CONFLICT OF INTEREST STATEMENT

Authors declare that they have no conflict of interest, including commercial or intellectual property interest in wild, local, or any germplasm.

### OPEN RESEARCH BADGES

This article has earned Open Data and Open Materials badges. Data and materials are available at https://doi.org/10.5061/dryad.c2fqz61d9.

## Supporting information


Appendix S1

## Data Availability

Occurrence points by domestication category, the complete ODMAP protocol report, and R‐markdown code for all analyses herein included are available at the following Dryad link: https://doi.org/10.5061/dryad.c2fqz61d9.
