# Supplementary material for: Fluctuation of ecological niches and geographic range shifts along chile pepper's domestication gradient
Source: Ecol Evol. 2023 Nov 28;13(11):e10731. doi: 10.1002/ece3.10731 (PMC10682905; doi:10.1002/ece3.10731)
Supplement: Supplementary file 1 — Appendix S1 [file ECE3-13-e10731-s001.zip › Appendix1_SuppFig_SA4.pdf]

Appendix 1 Supp. figure SA4

Future projections **sum** GCMs 2090 SSP: 2_45

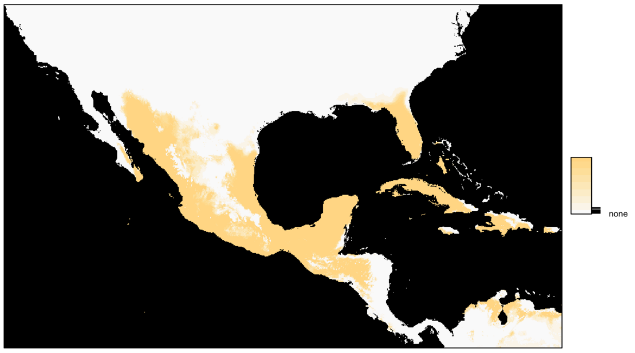

WILD

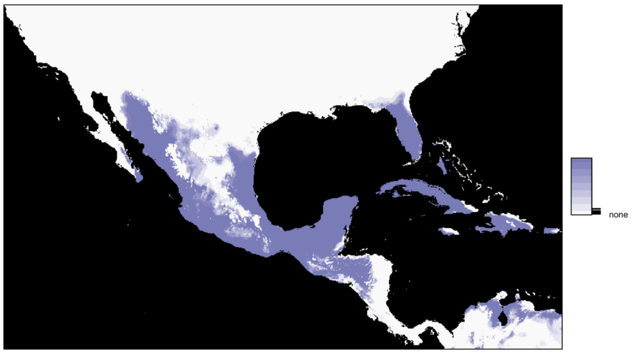

WILD_SL

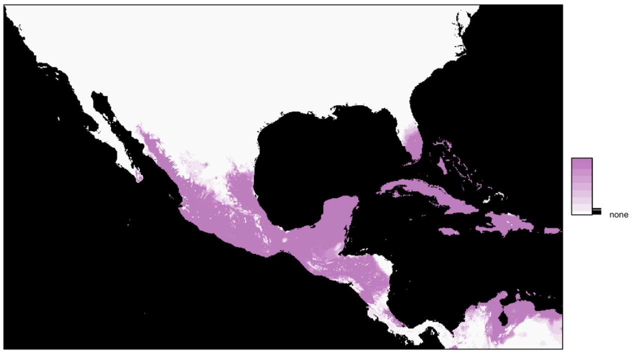

SEMIWILD

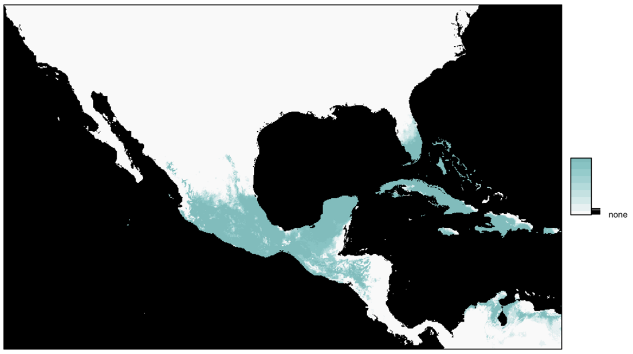

LANDRACE

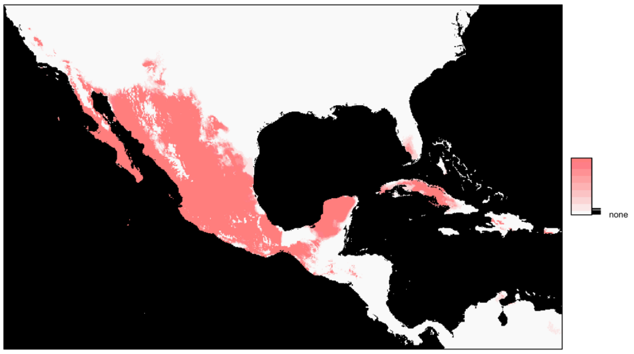

COMMERCIAL

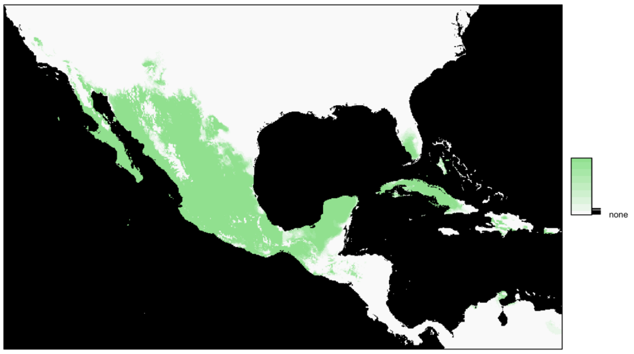

CULTIVATED

Future projections **sum** GCMs 2090 SSP: 5_85

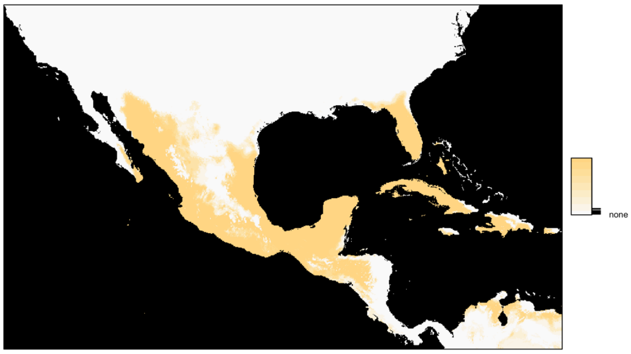

WILD

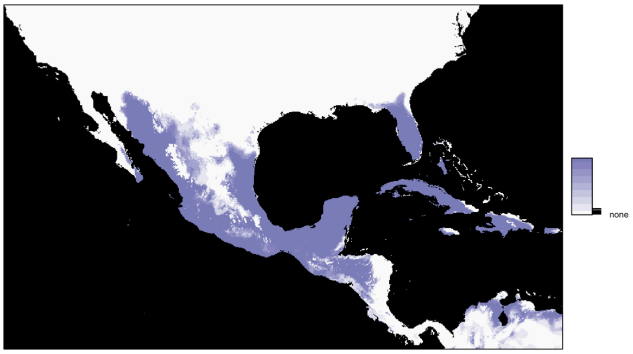

WILD_SL

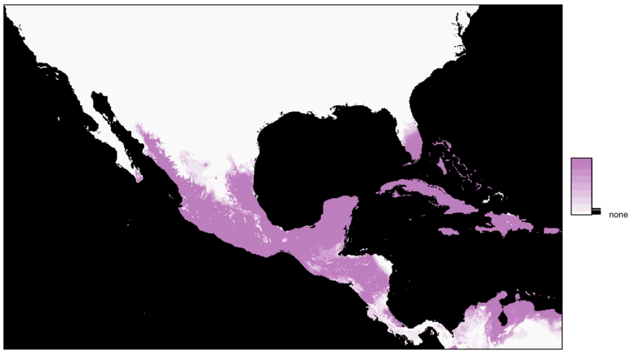

SEMIWILD

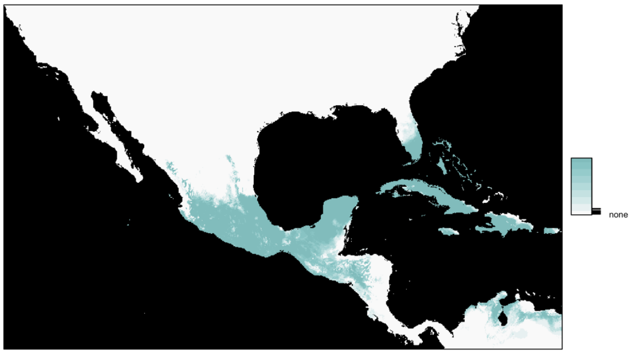

LANDRACE

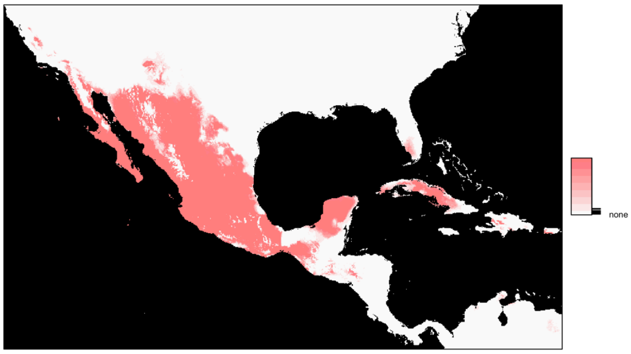

COMMERCIAL

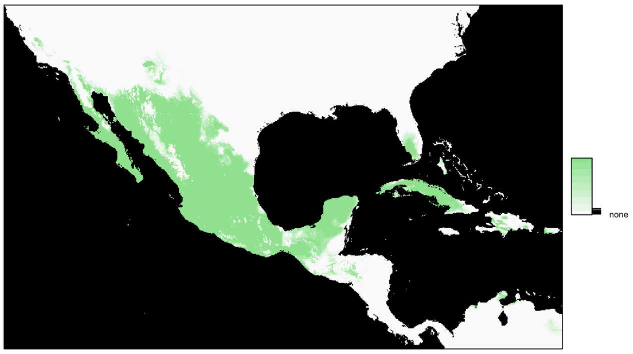

CULTIVATED

Future projections **intersections** GCMs 2090 SSP: 2_45

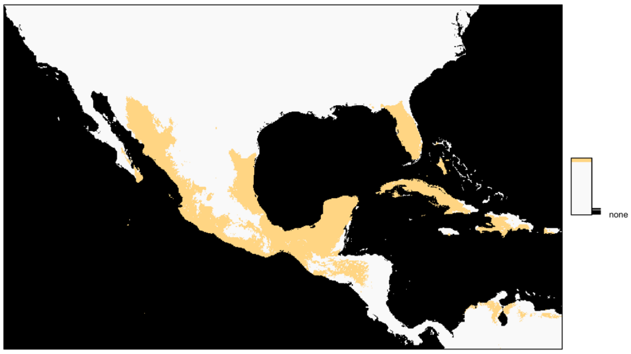

WILD

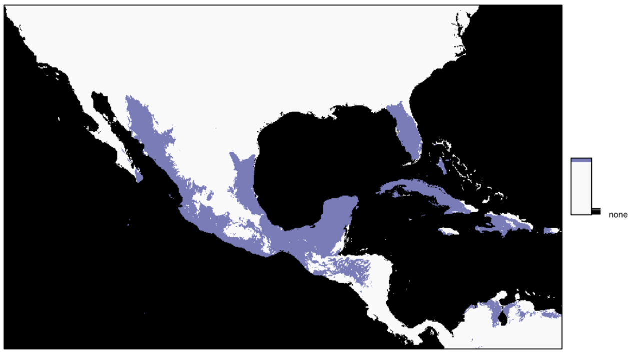

WILD_SL

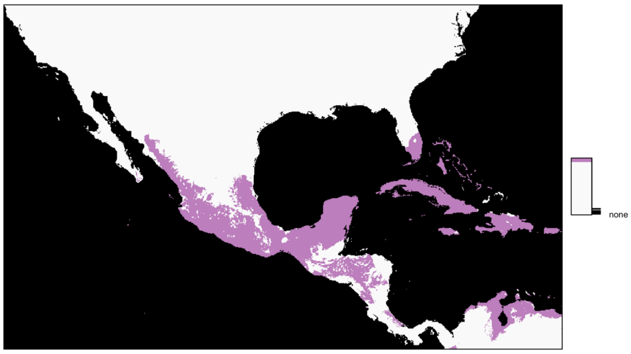

SEMIWILD

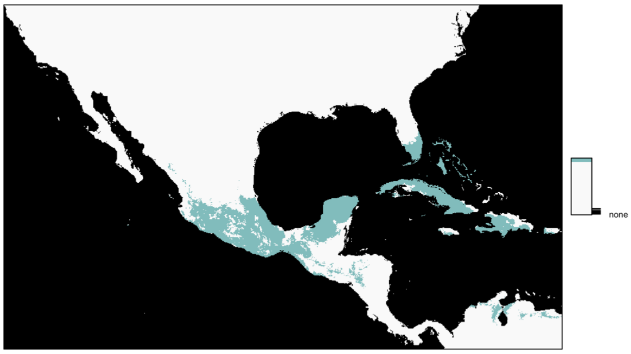

LANDRACE

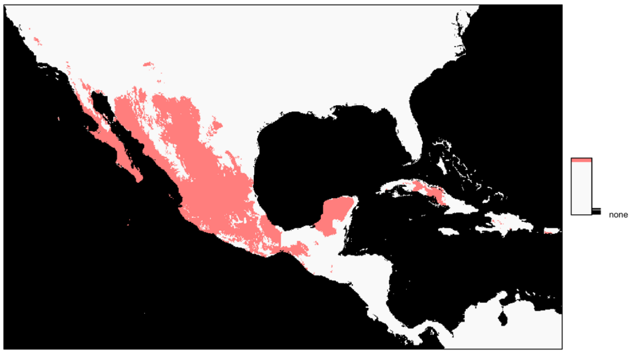

COMMERCIAL

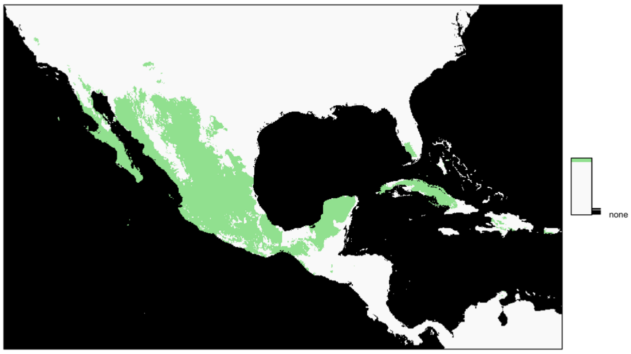

CULTIVATED

Future projections **intersections** GCMs 2090 SSP: 5_85

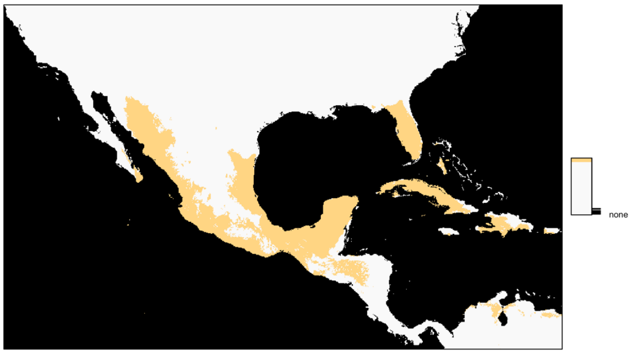

WILD

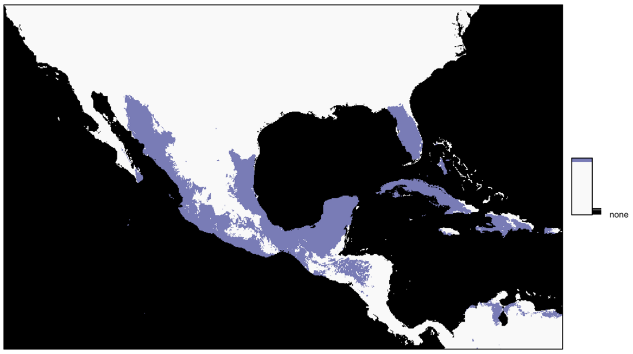

WILD_SL

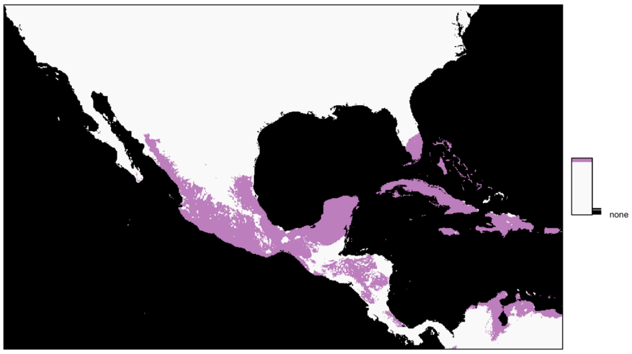

SEMIWILD

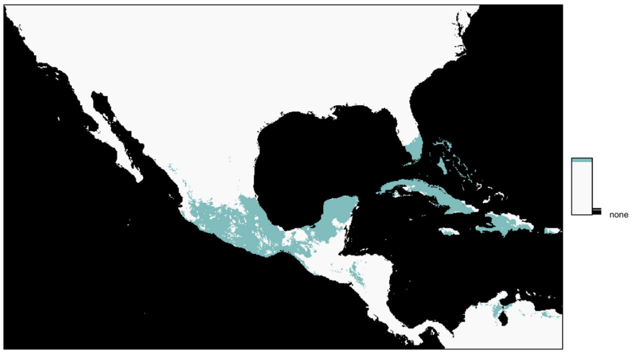

LANDRACE

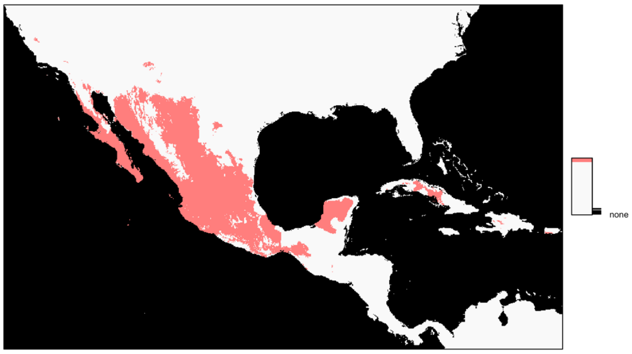

COMMERCIAL

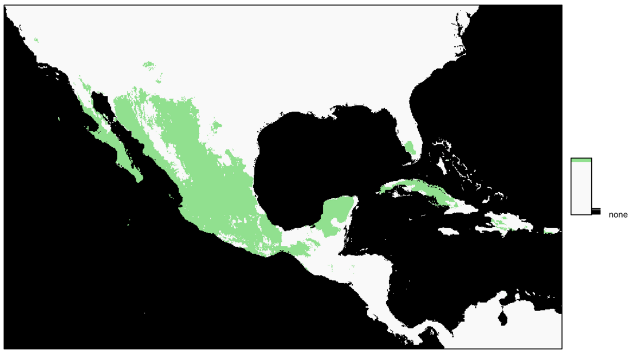

CULTIVATED
