# Supplementary material for: Fluctuation of ecological niches and geographic range shifts along chile pepper's domestication gradient
Source: Ecol Evol. 2023 Nov 28;13(11):e10731. doi: 10.1002/ece3.10731 (PMC10682905; doi:10.1002/ece3.10731)
Supplement: Supplementary file 1 — Appendix S1 [file ECE3-13-e10731-s001.zip › Appendix1_SuppFig_SA6.pdf]

Appendix 1 Supp. figure SA6

Pairwise domesticarton categories **future** projections for GCM intersections at 2050, SSP: 2_45

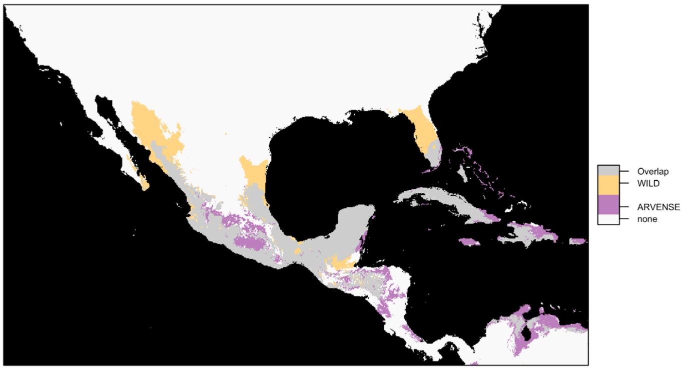

WILD *vs* SEMIWILD

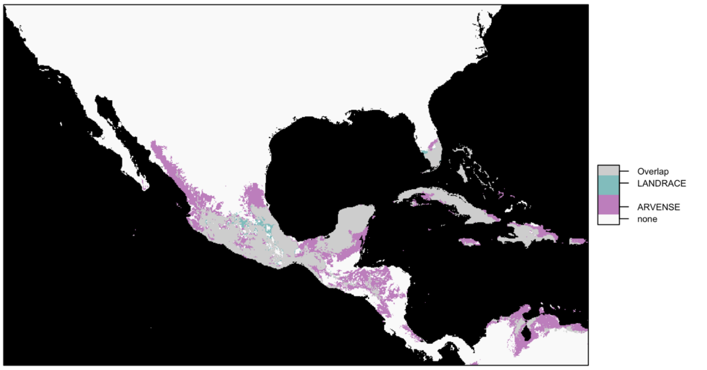

SEMIWILD *vs* LANDRACE

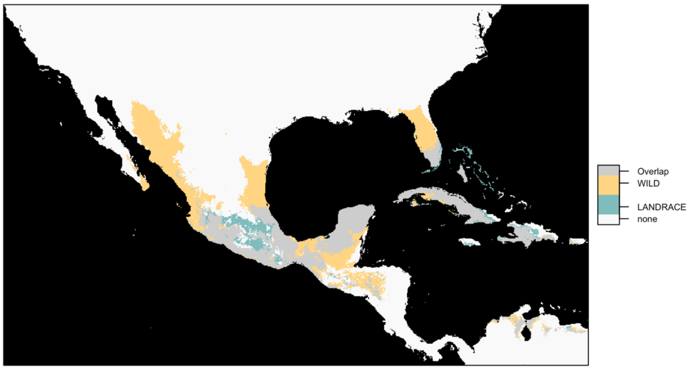

WILD *vs* LANDRACE

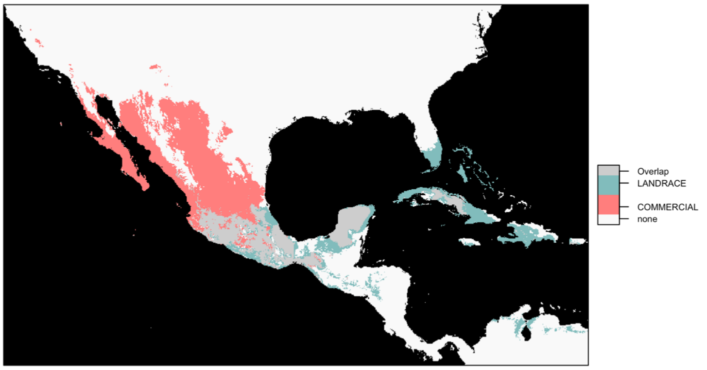

LANDRACE *vs* COMMERCIAL

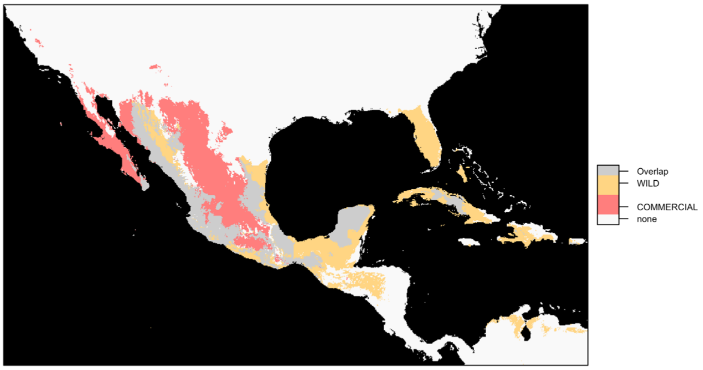

WILD *vs* COMMERCIAL

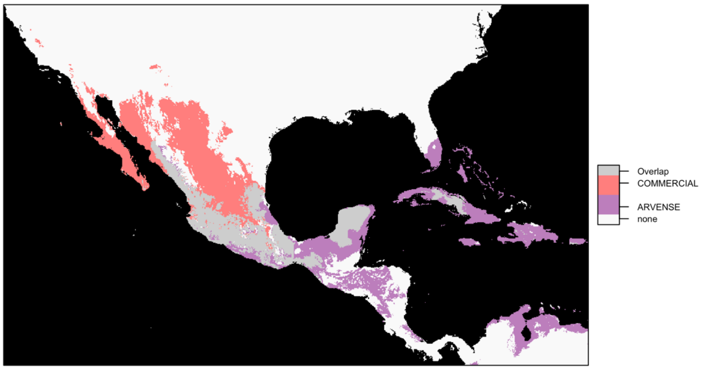

SEMIWILD *vs* COMMERCIAL

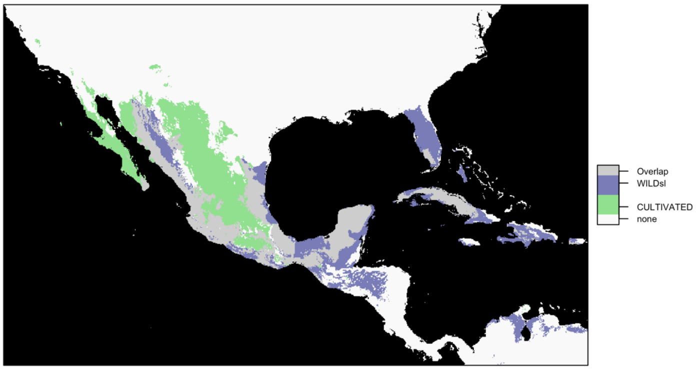

WILDsl *vs* CULTIVATED

Pairwise domesticarton categories future projections for GCM intersections at 2050, SSP: 4_85

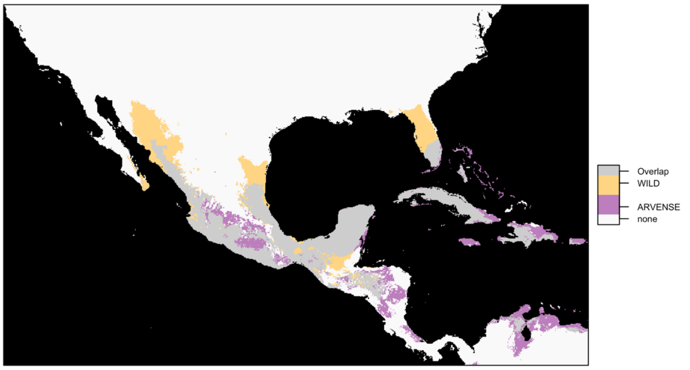

WILD *vs* SEMIWILD

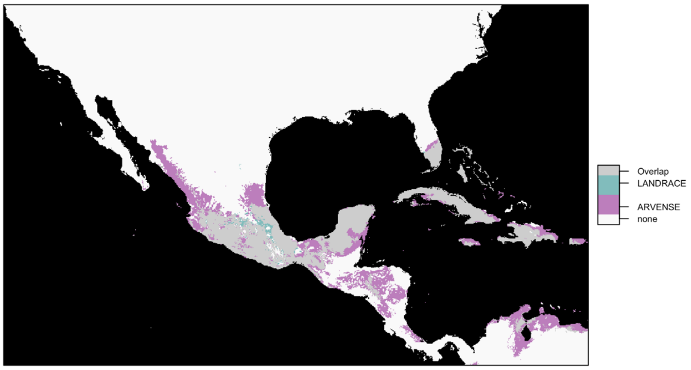

SEMIWILD *vs* LANDRACE

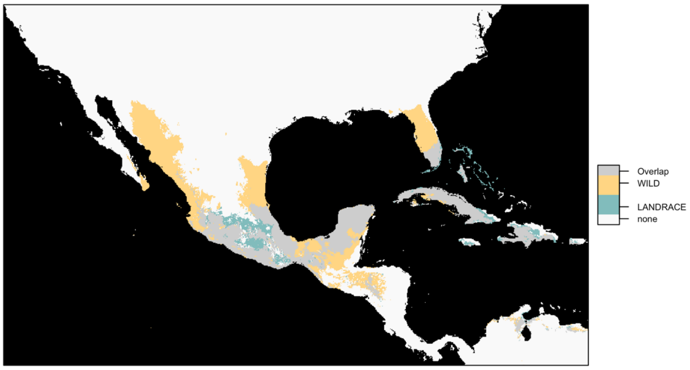

WILD *vs* LANDRACE

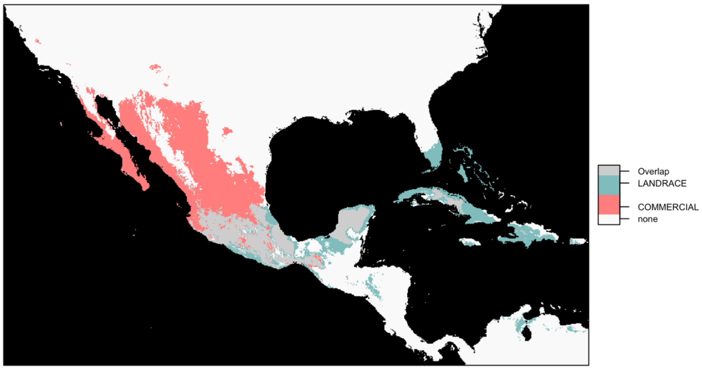

LANDRACE *vs* COMMERCIAL

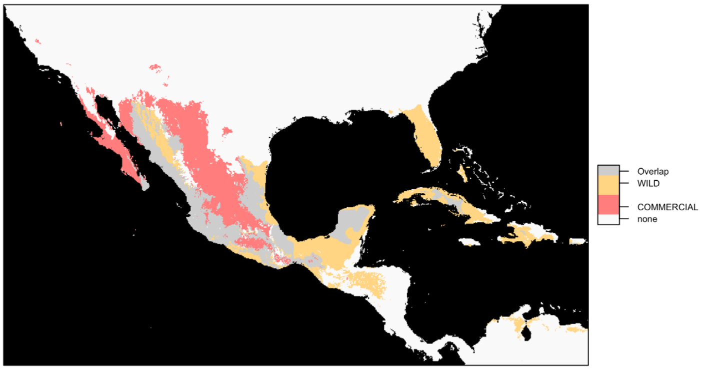

WILD *vs* COMMERCIAL

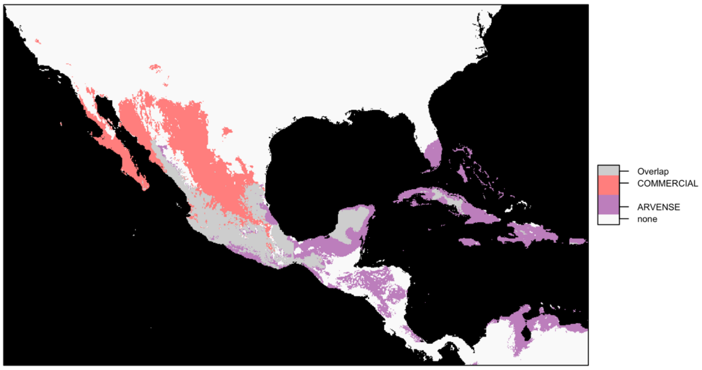

SEMIWILD *vs* COMMERCIAL

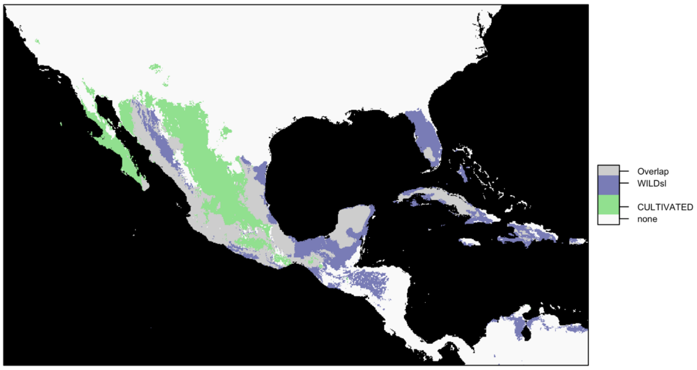

WILDsl *vs* CULTIVATED

Pairwise domesticarton categories **future** projections for GCM intersections at 2070, SSP: 2_45

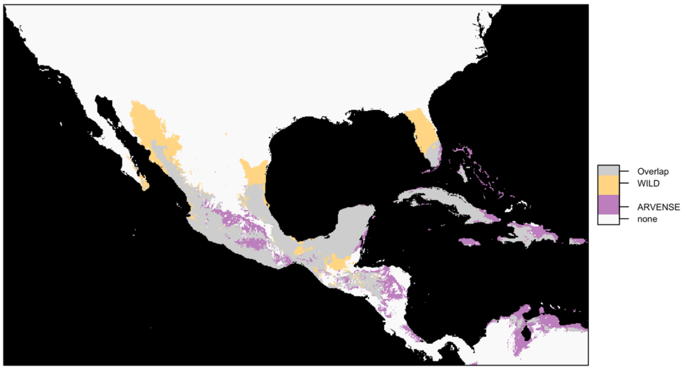

WILD *vs* SEMIWILD

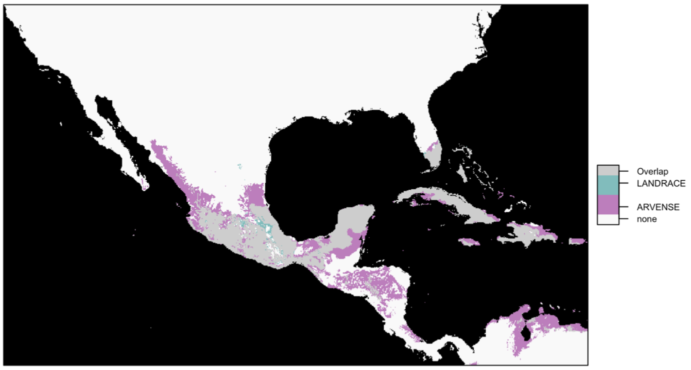

SEMIWILD *vs* LANDRACE

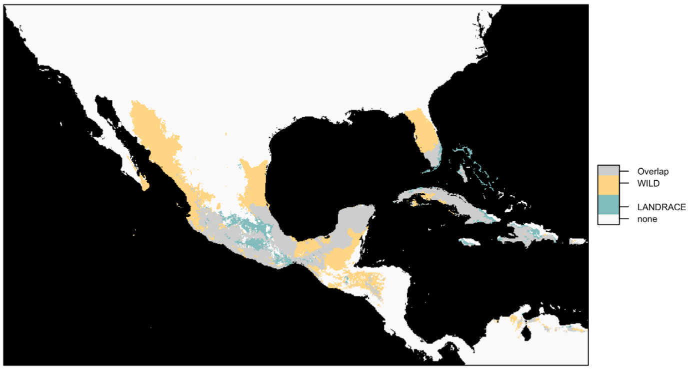

WILD *vs* LANDRACE

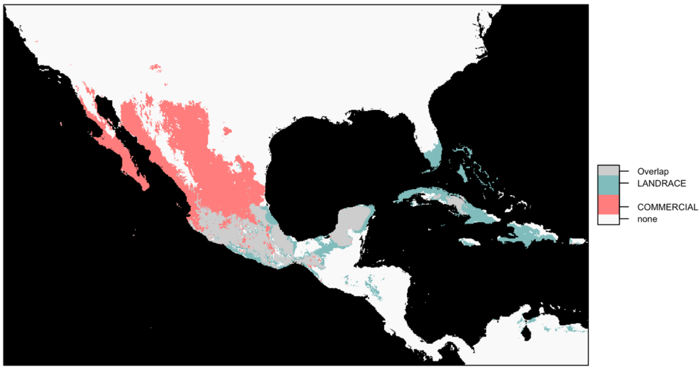

LANDRACE *vs* COMMERCIAL

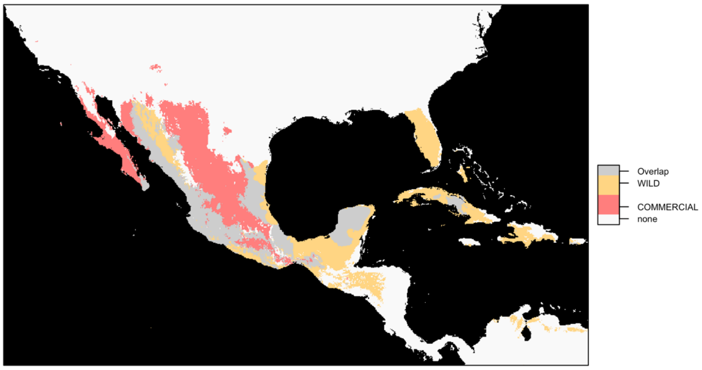

WILD *vs* COMMERCIAL

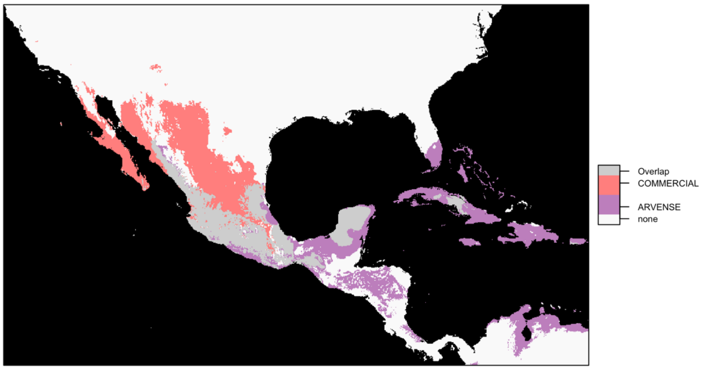

SEMIWILD *vs* COMMERCIAL

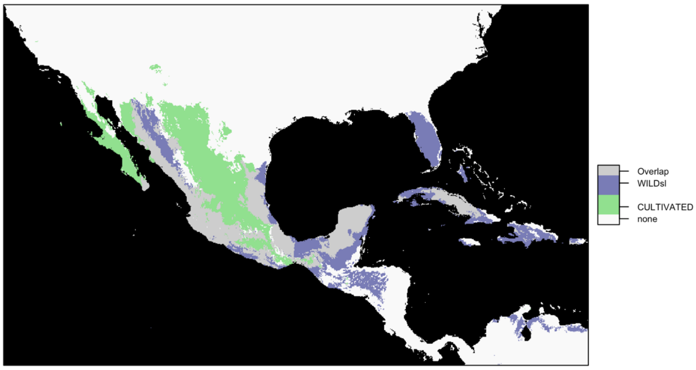

WILDsl *vs* CULTIVATED

Pairwise domesticarton categories **future** projections for GCM intersections at 2070, SSP: 4_85

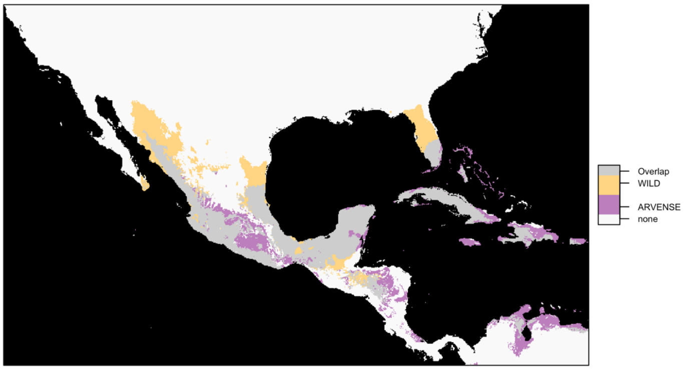

WILD *vs* SEMIWILD

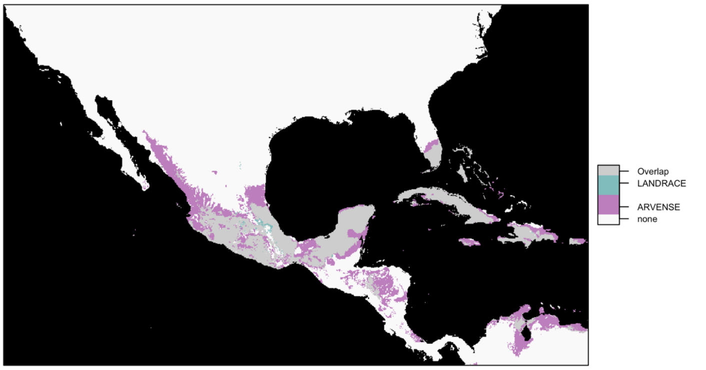

SEMIWILD *vs* LANDRACE

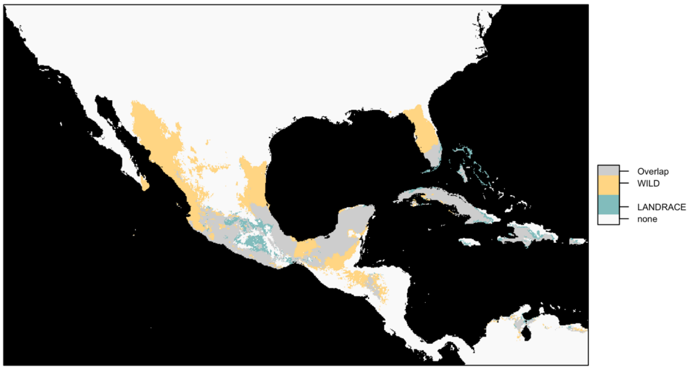

WILD *vs* LANDRACE

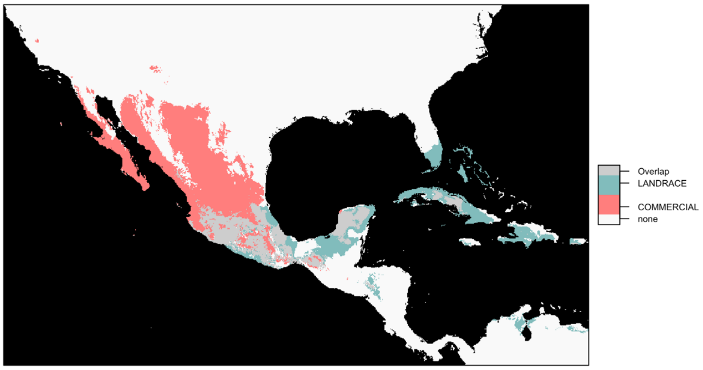

LANDRACE *vs* COMMERCIAL

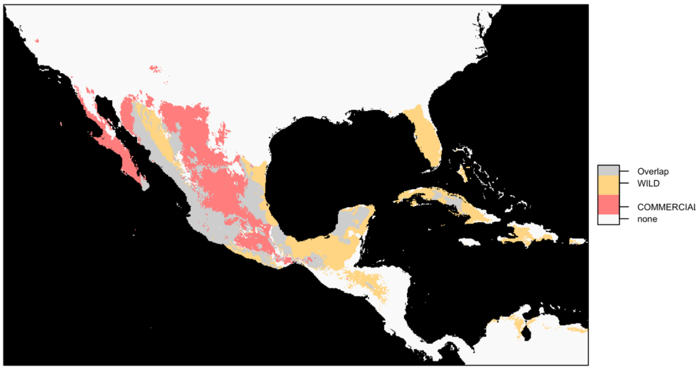

WILD *vs* COMMERCIAL

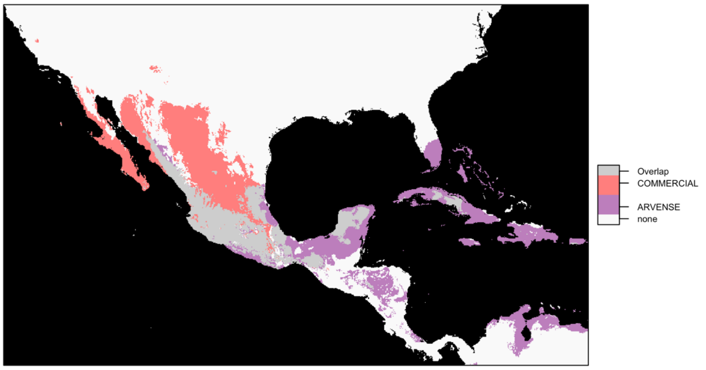

SEMIWILD *vs* COMMERCIAL

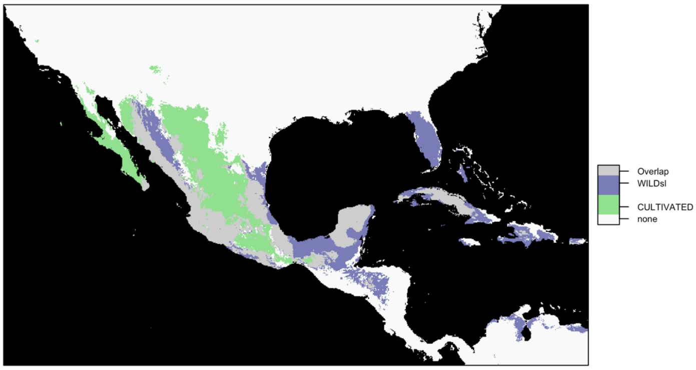

WILDsl *vs* CULTIVATED

Pairwise domesticarton categories **future** projections for GCM intersections at 2090, SSP: 2_45

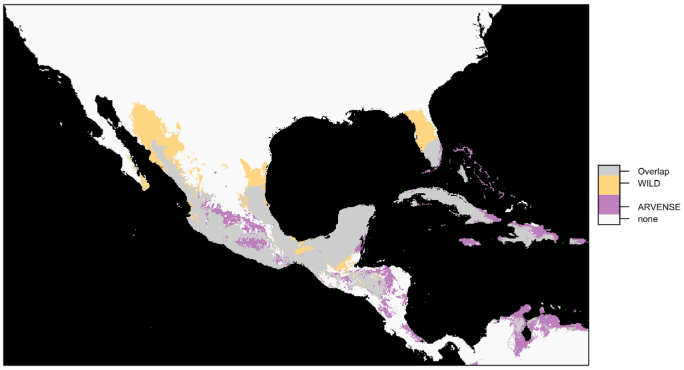

WILD *vs* SEMIWILD

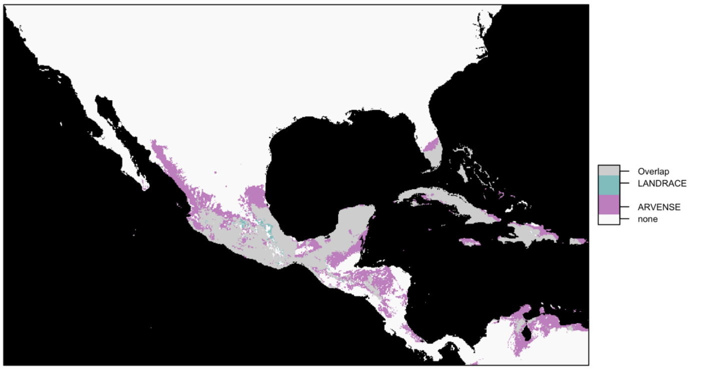

SEMIWILD *vs* LANDRACE

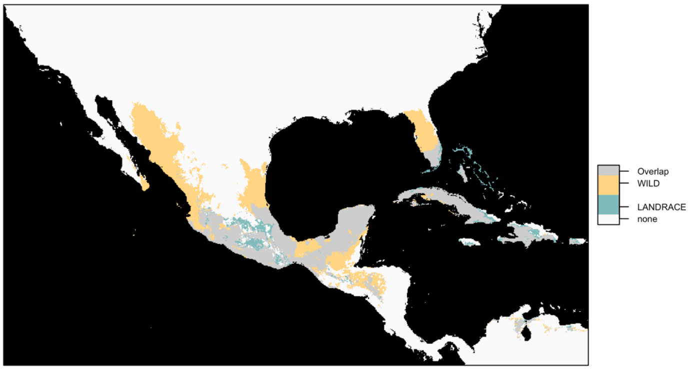

WILD *vs* LANDRACE

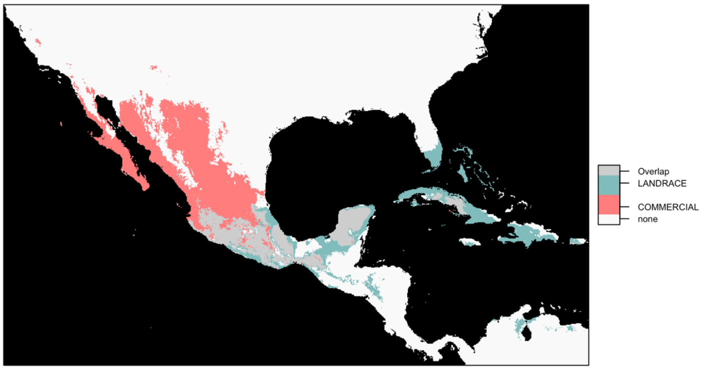

LANDRACE *vs* COMMERCIAL

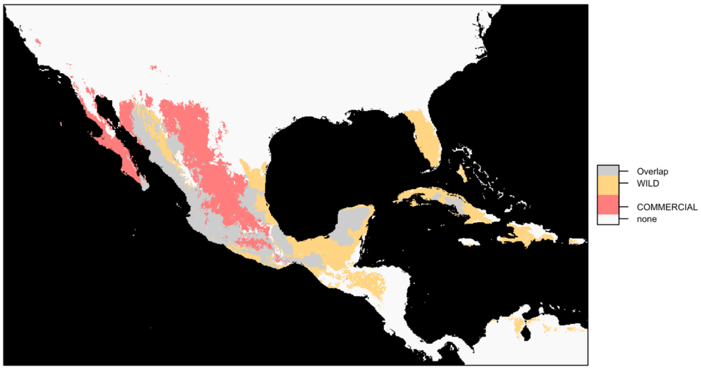

WILD *vs* COMMERCIAL

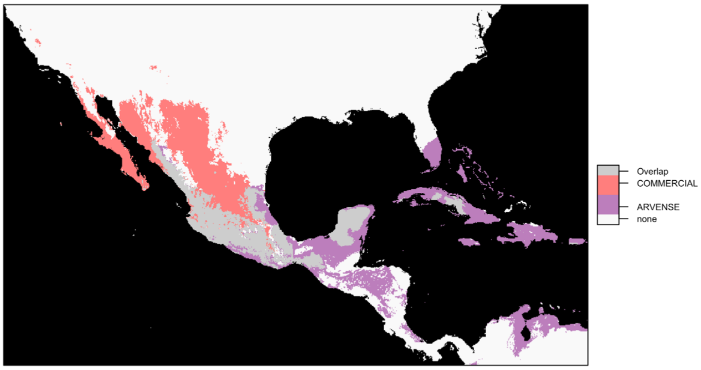

SEMIWILD *vs* COMMERCIAL

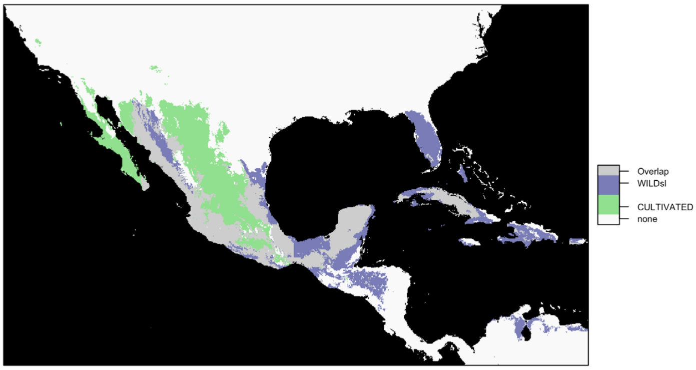

WILDsl *vs* CULTIVATED

Pairwise domesticarton categories **future** projections for GCM intersections at 2090, SSP: 4_85

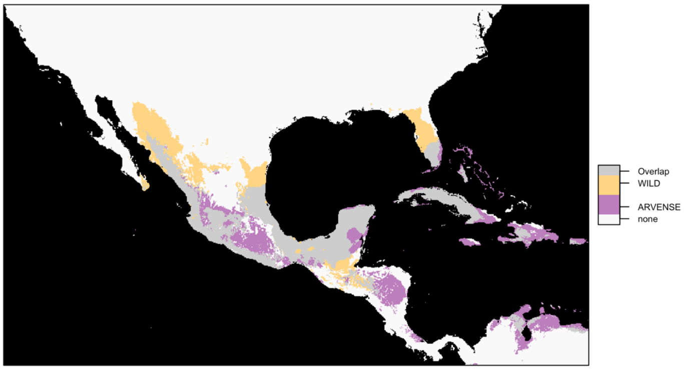

WILD *vs* SEMIWILD

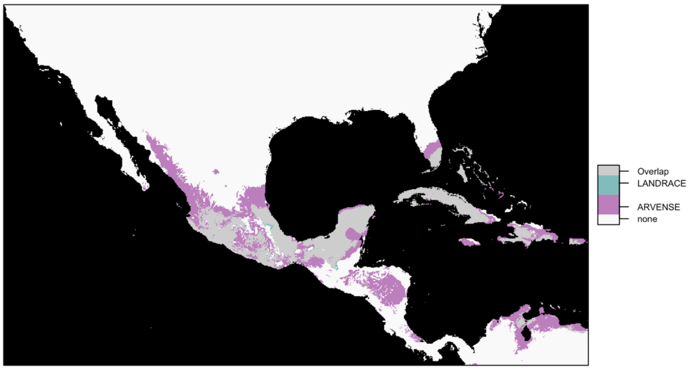

SEMIWILD *vs* LANDRACE

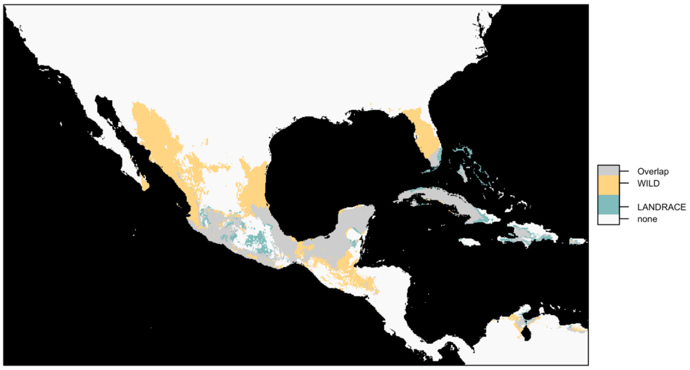

WILD *vs* LANDRACE

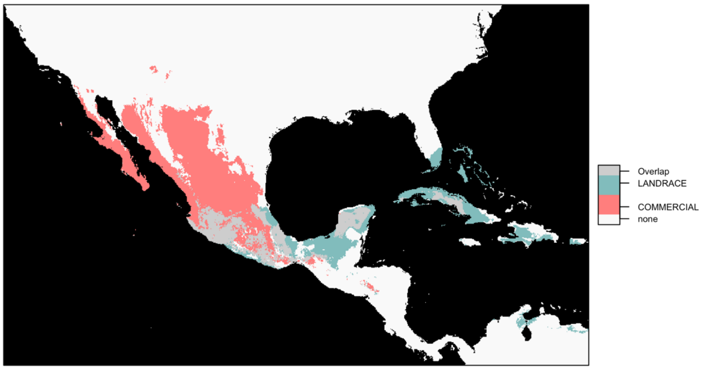

LANDRACE *vs* COMMERCIAL

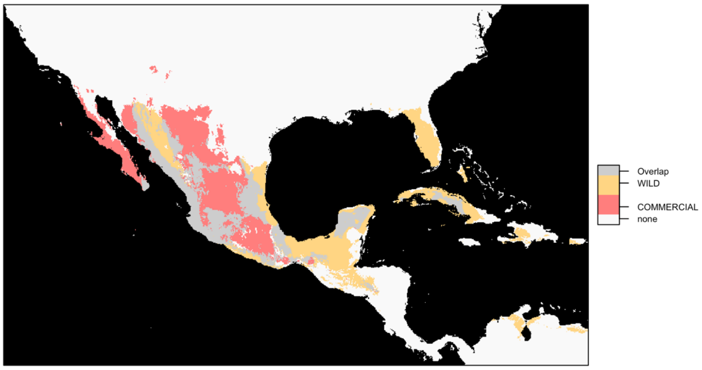

WILD *vs* COMMERCIAL

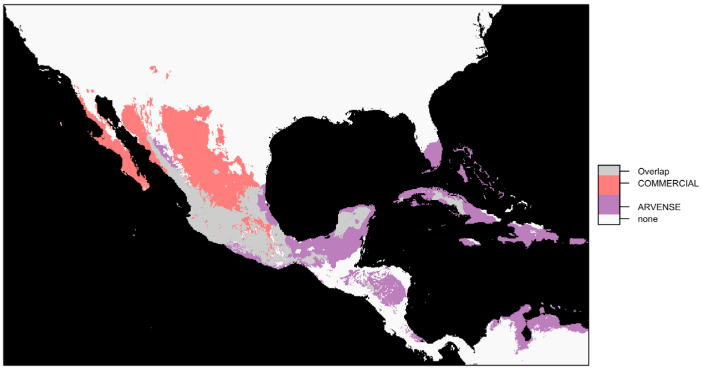

SEMIWILD *vs* COMMERCIAL

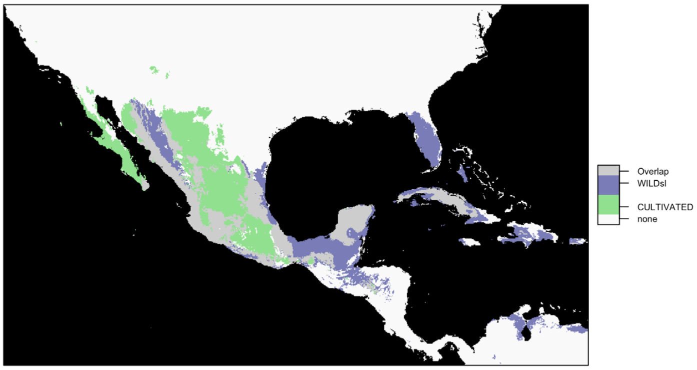

WILDsl *vs* CULTIVATED
