# Supplementary material for: Fluctuation of ecological niches and geographic range shifts along chile pepper's domestication gradient
Source: Ecol Evol. 2023 Nov 28;13(11):e10731. doi: 10.1002/ece3.10731 (PMC10682905; doi:10.1002/ece3.10731)
Supplement: Supplementary file 1 — Appendix S1 [file ECE3-13-e10731-s001.zip › Appendix1_SuppTable_SA2.docx]

**Supplementary Appendix table SA2**

| Type | settings | train.AUC | avg.test.AUC | var.test.AUC | avg.diff.AUC | var.diff.AUC | avg.test.orMTP | var.test.orMTP | avg.test.or10pct | var.test.or10pct | AICc | delta.AICc | w.AIC | parameters | TSS |
| --- | --- | --- | --- | --- | --- | --- | --- | --- | --- | --- | --- | --- | --- | --- | --- |
|  |  |  |  |  |  |  |  |  |  |  |  |  |  |  |  |
| WILD | L_0.5 | 0.786 | 0.778 | 0.002 | 0.018 | 0.001 | 0.007 | 0.000 | 0.123 | 0.010 | 6878.70 | 120.23 | 0.000 | 9 |  |
| WILD | LQ_0.5 | 0.822 | 0.809 | 0.000 | 0.015 | 0.001 | 0.007 | 0.000 | 0.124 | 0.003 | 6765.16 | 6.69 | 0.034 | 16 |  |
| WILD | LQH_0.5 | 0.860 | 0.815 | 0.000 | 0.047 | 0.000 | 0.014 | 0.000 | 0.146 | 0.002 | 7769.08 | 1010.61 | 0.000 | 78 |  |
| WILD | LQHP_0.5 | 0.865 | 0.828 | 0.000 | 0.038 | 0.001 | 0.013 | 0.000 | 0.147 | 0.002 | 7774.80 | 1016.33 | 0.000 | 84 |  |
| WILD | L_1 | 0.786 | 0.778 | 0.002 | 0.017 | 0.001 | 0.007 | 0.000 | 0.119 | 0.009 | 6879.52 | 121.06 | 0.000 | 9 |  |
| WILD | LQ_1 | 0.819 | 0.806 | 0.001 | 0.016 | 0.001 | 0.018 | 0.000 | 0.125 | 0.005 | 6775.27 | 16.80 | 0.000 | 15 |  |
| WILD | LQH_1 | 0.854 | 0.829 | 0.000 | 0.027 | 0.000 | 0.006 | 0.000 | 0.116 | 0.003 | 6925.17 | 166.70 | 0.000 | 102 |  |
| WILD | LQHP_1 | 0.863 | 0.839 | 0.000 | 0.027 | 0.000 | 0.009 | 0.000 | 0.142 | 0.002 | 6881.59 | 123.13 | 0.000 | 102 |  |
| WILD | L_1.5 | 0.786 | 0.777 | 0.002 | 0.017 | 0.001 | 0.007 | 0.000 | 0.122 | 0.009 | 6880.79 | 122.33 | 0.000 | 9 |  |
| WILD | LQ_1.5 | 0.814 | 0.801 | 0.001 | 0.016 | 0.001 | 0.011 | 0.000 | 0.122 | 0.004 | 6793.05 | 34.59 | 0.000 | 14 |  |
| WILD | LQH_1.5 | 0.848 | 0.827 | 0.000 | 0.023 | 0.000 | 0.006 | 0.000 | 0.115 | 0.003 | 6848.42 | 89.96 | 0.000 | 77 |  |
| WILD | LQHP_1.5 | 0.858 | 0.836 | 0.000 | 0.023 | 0.000 | 0.006 | 0.000 | 0.120 | 0.001 | 6809.18 | 50.71 | 0.000 | 77 |  |
| WILD | L_2 | 0.786 | 0.777 | 0.002 | 0.017 | 0.001 | 0.007 | 0.000 | 0.122 | 0.009 | 6882.55 | 124.08 | 0.000 | 9 |  |
| WILD | LQ_2 | 0.809 | 0.795 | 0.001 | 0.017 | 0.001 | 0.014 | 0.000 | 0.126 | 0.006 | 6807.00 | 48.54 | 0.000 | 12 |  |
| WILD | LQH_2 | 0.844 | 0.823 | 0.000 | 0.020 | 0.000 | 0.004 | 0.000 | 0.119 | 0.003 | 6785.58 | 27.11 | 0.000 | 51 |  |
| WILD | LQHP_2 | 0.852 | 0.831 | 0.000 | 0.020 | 0.001 | 0.006 | 0.000 | 0.114 | 0.002 | 6787.75 | 29.28 | 0.000 | 63 |  |
| WILD | L_2.5 | 0.785 | 0.777 | 0.002 | 0.017 | 0.001 | 0.007 | 0.000 | 0.122 | 0.009 | 6884.96 | 126.49 | 0.000 | 9 |  |
| WILD | LQ_2.5 | 0.802 | 0.790 | 0.001 | 0.017 | 0.001 | 0.011 | 0.000 | 0.133 | 0.006 | 6824.22 | 65.75 | 0.000 | 11 |  |
| WILD | LQH_2.5 | 0.839 | 0.819 | 0.000 | 0.018 | 0.001 | 0.004 | 0.000 | 0.110 | 0.003 | 6776.38 | 17.91 | 0.000 | 42 |  |
| WILD | LQHP_2.5 | 0.846 | 0.826 | 0.000 | 0.017 | 0.001 | 0.006 | 0.000 | 0.114 | 0.002 | 6772.67 | 14.20 | 0.001 | 50 |  |
| WILD | L_3 | 0.784 | 0.776 | 0.001 | 0.017 | 0.001 | 0.004 | 0.000 | 0.135 | 0.010 | 6887.63 | 129.16 | 0.000 | 9 |  |
| WILD | LQ_3 | 0.799 | 0.787 | 0.001 | 0.017 | 0.001 | 0.011 | 0.000 | 0.123 | 0.004 | 6829.95 | 71.48 | 0.000 | 10 |  |
| WILD | LQH_3 | 0.833 | 0.815 | 0.001 | 0.016 | 0.001 | 0.004 | 0.000 | 0.106 | 0.003 | 6779.15 | 20.69 | 0.000 | 36 |  |
| **WILD** | **LQHP_3** | **0.839** | **0.821** | **0.001** | **0.017** | **0.001** | **0.006** | **0.000** | **0.107** | **0.003** | **6758.47** | **0.00** | **0.965** | **35** | **0.626** |
| WILD | L_3.5 | 0.784 | 0.776 | 0.001 | 0.017 | 0.001 | 0.006 | 0.000 | 0.134 | 0.010 | 6890.91 | 132.44 | 0.000 | 9 |  |
| WILD | LQ_3.5 | 0.797 | 0.786 | 0.001 | 0.018 | 0.001 | 0.007 | 0.000 | 0.117 | 0.004 | 6836.13 | 77.67 | 0.000 | 10 |  |
| WILD | LQH_3.5 | 0.828 | 0.811 | 0.001 | 0.016 | 0.001 | 0.004 | 0.000 | 0.109 | 0.003 | 6783.05 | 24.58 | 0.000 | 31 |  |
| WILD | LQHP_3.5 | 0.832 | 0.816 | 0.001 | 0.017 | 0.001 | 0.006 | 0.000 | 0.109 | 0.003 | 6779.52 | 21.05 | 0.000 | 35 |  |
|  |  |  |  |  |  |  |  |  |  |  |  |  |  |  |  |
| WILD-SL | L_0.5 | 0.790 | 0.783 | 0.002 | 0.015 | 0.001 | 0.005 | 0.000 | 0.115 | 0.007 | 8328.98 | 133.68 | 0.000 | 9 |  |
| WILD-SL | LQ_0.5 | 0.826 | 0.816 | 0.001 | 0.015 | 0.001 | 0.010 | 0.000 | 0.127 | 0.001 | 8208.89 | 13.59 | 0.001 | 18 |  |
| WILD-SL | LQH_0.5 | 0.859 | 0.831 | 0.000 | 0.032 | 0.000 | 0.013 | 0.000 | 0.133 | 0.001 | 9085.51 | 890.21 | 0.000 | 61 |  |
| WILD-SL | LQHP_0.5 | 0.871 | 0.840 | 0.000 | 0.031 | 0.000 | 0.011 | 0.000 | 0.139 | 0.000 | 8892.19 | 696.89 | 0.000 | 122 |  |
| WILD-SL | L_1 | 0.789 | 0.783 | 0.002 | 0.015 | 0.001 | 0.005 | 0.000 | 0.118 | 0.007 | 8330.18 | 134.88 | 0.000 | 9 |  |
| WILD-SL | LQ_1 | 0.821 | 0.812 | 0.001 | 0.015 | 0.002 | 0.010 | 0.000 | 0.117 | 0.003 | 8216.93 | 21.63 | 0.000 | 15 |  |
| WILD-SL | LQH_1 | 0.855 | 0.834 | 0.001 | 0.023 | 0.001 | 0.005 | 0.000 | 0.130 | 0.002 | 8375.95 | 180.65 | 0.000 | 115 |  |
| WILD-SL | LQHP_1 | 0.864 | 0.843 | 0.001 | 0.023 | 0.001 | 0.005 | 0.000 | 0.135 | 0.002 | 8280.55 | 85.25 | 0.000 | 104 |  |
| WILD-SL | L_1.5 | 0.789 | 0.783 | 0.002 | 0.015 | 0.001 | 0.005 | 0.000 | 0.115 | 0.007 | 8331.98 | 136.68 | 0.000 | 9 |  |
| WILD-SL | LQ_1.5 | 0.816 | 0.807 | 0.002 | 0.015 | 0.002 | 0.010 | 0.000 | 0.111 | 0.003 | 8236.32 | 41.02 | 0.000 | 15 |  |
| WILD-SL | LQH_1.5 | 0.851 | 0.831 | 0.001 | 0.020 | 0.001 | 0.005 | 0.000 | 0.117 | 0.002 | 8282.73 | 87.43 | 0.000 | 84 |  |
| WILD-SL | LQHP_1.5 | 0.859 | 0.840 | 0.001 | 0.020 | 0.001 | 0.005 | 0.000 | 0.118 | 0.003 | 8217.68 | 22.38 | 0.000 | 78 |  |
| WILD-SL | L_2 | 0.789 | 0.782 | 0.002 | 0.015 | 0.001 | 0.005 | 0.000 | 0.113 | 0.007 | 8334.37 | 139.07 | 0.000 | 9 |  |
| WILD-SL | LQ_2 | 0.810 | 0.802 | 0.002 | 0.015 | 0.002 | 0.008 | 0.000 | 0.113 | 0.004 | 8254.96 | 59.66 | 0.000 | 13 |  |
| WILD-SL | LQH_2 | 0.845 | 0.829 | 0.001 | 0.018 | 0.001 | 0.005 | 0.000 | 0.117 | 0.002 | 8291.05 | 95.75 | 0.000 | 79 |  |
| WILD-SL | LQHP_2 | 0.853 | 0.837 | 0.001 | 0.018 | 0.001 | 0.010 | 0.000 | 0.103 | 0.001 | 8243.47 | 48.17 | 0.000 | 77 |  |
| WILD-SL | L_2.5 | 0.788 | 0.782 | 0.002 | 0.015 | 0.001 | 0.005 | 0.000 | 0.115 | 0.007 | 8337.22 | 141.92 | 0.000 | 9 |  |
| WILD-SL | LQ_2.5 | 0.804 | 0.796 | 0.002 | 0.015 | 0.002 | 0.008 | 0.000 | 0.109 | 0.004 | 8272.26 | 76.96 | 0.000 | 12 |  |
| WILD-SL | LQH_2.5 | 0.841 | 0.827 | 0.001 | 0.016 | 0.001 | 0.005 | 0.000 | 0.119 | 0.002 | 8247.19 | 51.89 | 0.000 | 59 |  |
| WILD-SL | LQHP_2.5 | 0.848 | 0.833 | 0.001 | 0.017 | 0.001 | 0.010 | 0.000 | 0.108 | 0.001 | 8218.64 | 23.34 | 0.000 | 60 |  |
| WILD-SL | L_3 | 0.787 | 0.781 | 0.002 | 0.014 | 0.001 | 0.005 | 0.000 | 0.118 | 0.006 | 8338.42 | 143.12 | 0.000 | 8 |  |
| WILD-SL | LQ_3 | 0.801 | 0.793 | 0.002 | 0.015 | 0.002 | 0.008 | 0.000 | 0.106 | 0.003 | 8279.46 | 84.16 | 0.000 | 10 |  |
| WILD-SL | LQH_3 | 0.837 | 0.824 | 0.001 | 0.016 | 0.001 | 0.005 | 0.000 | 0.108 | 0.002 | 8242.50 | 47.20 | 0.000 | 51 |  |
| **WILD-SL** | **LQHP_3** | **0.841** | **0.830** | **0.001** | **0.017** | **0.001** | **0.008** | **0.000** | **0.103** | **0.001** | **8195.30** | **0.00** | **0.788** | **41** | **0.588** |
| WILD-SL | L_3.5 | 0.786 | 0.781 | 0.001 | 0.014 | 0.001 | 0.005 | 0.000 | 0.124 | 0.008 | 8341.35 | 146.05 | 0.000 | 8 |  |
| WILD-SL | LQ_3.5 | 0.799 | 0.792 | 0.002 | 0.015 | 0.002 | 0.005 | 0.000 | 0.109 | 0.004 | 8288.13 | 92.83 | 0.000 | 10 |  |
| WILD-SL | LQH_3.5 | 0.831 | 0.820 | 0.001 | 0.015 | 0.001 | 0.005 | 0.000 | 0.106 | 0.002 | 8229.11 | 33.81 | 0.000 | 39 |  |
| WILD-SL | LQHP_3.5 | 0.837 | 0.828 | 0.001 | 0.016 | 0.001 | 0.008 | 0.000 | 0.103 | 0.002 | 8197.93 | 2.63 | 0.211 | 35 |  |
|  |  |  |  |  |  |  |  |  |  |  |  |  |  |  |  |
| SEMIWILD | L_0.5 | 0.867 | 0.856 | 0.002 | 0.022 | 0.001 | 0.012 | 0.001 | 0.122 | 0.009 | 1892.10 | 17.75 | 0.000 | 9 |  |
| SEMIWILD | LQ_0.5 | 0.885 | 0.867 | 0.001 | 0.022 | 0.000 | 0.012 | 0.001 | 0.214 | 0.004 | 1882.77 | 8.42 | 0.007 | 15 |  |
| SEMIWILD | LQH_0.5 | 0.915 | 0.875 | 0.001 | 0.043 | 0.001 | 0.012 | 0.001 | 0.205 | 0.005 | NA | NA | NA | 105 |  |
| SEMIWILD | LQHP_0.5 | 0.918 | 0.872 | 0.000 | 0.048 | 0.000 | 0.012 | 0.001 | 0.270 | 0.021 | NA | NA | NA | 103 |  |
| SEMIWILD | L_1 | 0.867 | 0.857 | 0.001 | 0.021 | 0.001 | 0.012 | 0.001 | 0.134 | 0.010 | 1892.50 | 18.15 | 0.000 | 9 |  |
| SEMIWILD | LQ_1 | 0.883 | 0.863 | 0.001 | 0.022 | 0.001 | 0.012 | 0.001 | 0.153 | 0.007 | 1878.10 | 3.75 | 0.075 | 12 |  |
| SEMIWILD | LQH_1 | 0.901 | 0.873 | 0.001 | 0.031 | 0.001 | 0.012 | 0.001 | 0.172 | 0.005 | 2099.76 | 225.40 | 0.000 | 53 |  |
| SEMIWILD | LQHP_1 | 0.905 | 0.870 | 0.001 | 0.036 | 0.001 | 0.012 | 0.001 | 0.231 | 0.010 | 2177.60 | 303.24 | 0.000 | 58 |  |
| SEMIWILD | L_1.5 | 0.867 | 0.857 | 0.001 | 0.020 | 0.001 | 0.012 | 0.001 | 0.145 | 0.013 | 1890.60 | 16.25 | 0.000 | 8 |  |
| SEMIWILD | LQ_1.5 | 0.879 | 0.858 | 0.001 | 0.023 | 0.001 | 0.012 | 0.001 | 0.187 | 0.016 | 1879.72 | 5.37 | 0.033 | 11 |  |
| SEMIWILD | LQH_1.5 | 0.893 | 0.867 | 0.001 | 0.026 | 0.001 | 0.012 | 0.001 | 0.182 | 0.008 | 1895.45 | 21.09 | 0.000 | 24 |  |
| SEMIWILD | LQHP_1.5 | 0.895 | 0.869 | 0.001 | 0.026 | 0.001 | 0.012 | 0.001 | 0.167 | 0.007 | 1914.35 | 40.00 | 0.000 | 29 |  |
| SEMIWILD | L_2 | 0.867 | 0.856 | 0.001 | 0.019 | 0.001 | 0.012 | 0.001 | 0.155 | 0.010 | 1888.84 | 14.48 | 0.000 | 7 |  |
| SEMIWILD | LQ_2 | 0.876 | 0.856 | 0.001 | 0.022 | 0.001 | 0.012 | 0.001 | 0.164 | 0.007 | 1881.39 | 7.04 | 0.014 | 10 |  |
| SEMIWILD | LQH_2 | 0.888 | 0.863 | 0.001 | 0.025 | 0.001 | 0.012 | 0.001 | 0.182 | 0.008 | 1876.24 | 1.89 | 0.190 | 16 |  |
| SEMIWILD | LQHP_2 | 0.892 | 0.865 | 0.001 | 0.022 | 0.001 | 0.012 | 0.001 | 0.141 | 0.004 | 1880.35 | 6.00 | 0.024 | 19 |  |
| SEMIWILD | L_2.5 | 0.867 | 0.855 | 0.001 | 0.018 | 0.001 | 0.012 | 0.001 | 0.140 | 0.012 | 1889.51 | 15.16 | 0.000 | 7 |  |
| SEMIWILD | LQ_2.5 | 0.874 | 0.854 | 0.001 | 0.022 | 0.001 | 0.012 | 0.001 | 0.176 | 0.011 | 1885.19 | 10.83 | 0.002 | 10 |  |
| SEMIWILD | LQH_2.5 | 0.884 | 0.860 | 0.001 | 0.023 | 0.001 | 0.012 | 0.001 | 0.171 | 0.006 | 1874.35 | 0.00 | 0.487 | 13 |  |
| SEMIWILD | LQHP_2.5 | 0.888 | 0.863 | 0.001 | 0.022 | 0.001 | 0.012 | 0.001 | 0.141 | 0.004 | 1878.59 | 4.24 | 0.058 | 16 |  |
| SEMIWILD | L_3 | 0.866 | 0.853 | 0.001 | 0.017 | 0.001 | 0.012 | 0.001 | 0.137 | 0.021 | 1890.34 | 15.98 | 0.000 | 7 |  |
| SEMIWILD | LQ_3 | 0.870 | 0.851 | 0.001 | 0.021 | 0.001 | 0.012 | 0.001 | 0.160 | 0.034 | 1889.64 | 15.29 | 0.000 | 10 |  |
| SEMIWILD | LQH_3 | 0.882 | 0.858 | 0.001 | 0.023 | 0.001 | 0.012 | 0.001 | 0.194 | 0.007 | 1883.29 | 8.94 | 0.006 | 14 |  |
| **SEMIWILD** | **LQHP_3** | **0.886** | **0.859** | **0.001** | **0.022** | **0.001** | **0.012** | **0.001** | **0.153** | **0.005** | **1881.67** | **7.32** | **0.013** | **15** | **0.649** |
| SEMIWILD | L_3.5 | 0.865 | 0.850 | 0.001 | 0.017 | 0.001 | 0.012 | 0.001 | 0.137 | 0.021 | 1891.28 | 16.93 | 0.000 | 7 |  |
| SEMIWILD | LQ_3.5 | 0.869 | 0.850 | 0.001 | 0.021 | 0.001 | 0.012 | 0.001 | 0.160 | 0.034 | 1889.08 | 14.72 | 0.000 | 9 |  |
| SEMIWILD | LQH_3.5 | 0.879 | 0.857 | 0.001 | 0.022 | 0.001 | 0.012 | 0.001 | 0.150 | 0.007 | 1881.50 | 7.15 | 0.014 | 11 |  |
| SEMIWILD | LQHP_3.5 | 0.883 | 0.857 | 0.001 | 0.022 | 0.001 | 0.012 | 0.001 | 0.143 | 0.007 | 1878.10 | 3.74 | 0.075 | 12 |  |
|  |  |  |  |  |  |  |  |  |  |  |  |  |  |  |  |
| LANDRACE | L_0.5 | 0.894 | 0.892 | 0.000 | 0.006 | 0.000 | 0.006 | 0.000 | 0.100 | 0.003 | 5926.41 | 44.97 | 0.000 | 9 |  |
| LANDRACE | LQ_0.5 | 0.904 | 0.900 | 0.000 | 0.007 | 0.000 | 0.003 | 0.000 | 0.103 | 0.004 | 5881.43 | 0.00 | 0.546 | 17 |  |
| LANDRACE | LQH_0.5 | 0.922 | 0.903 | 0.000 | 0.021 | 0.000 | 0.006 | 0.000 | 0.140 | 0.003 | 6666.10 | 784.67 | 0.000 | 102 |  |
| LANDRACE | LQHP_0.5 | 0.923 | 0.904 | 0.000 | 0.022 | 0.000 | 0.006 | 0.000 | 0.170 | 0.001 | 6553.49 | 672.05 | 0.000 | 78 |  |
| LANDRACE | L_1 | 0.894 | 0.892 | 0.000 | 0.006 | 0.000 | 0.006 | 0.000 | 0.100 | 0.003 | 5928.18 | 46.75 | 0.000 | 9 |  |
| LANDRACE | LQ_1 | 0.897 | 0.895 | 0.000 | 0.008 | 0.000 | 0.006 | 0.000 | 0.103 | 0.003 | 5917.66 | 36.23 | 0.000 | 16 |  |
| LANDRACE | LQH_1 | 0.916 | 0.905 | 0.000 | 0.012 | 0.000 | 0.003 | 0.000 | 0.132 | 0.005 | 5990.23 | 108.80 | 0.000 | 78 |  |
| LANDRACE | LQHP_1 | 0.920 | 0.910 | 0.000 | 0.012 | 0.000 | 0.003 | 0.000 | 0.134 | 0.003 | 6070.36 | 188.93 | 0.000 | 84 |  |
| LANDRACE | L_1.5 | 0.893 | 0.892 | 0.000 | 0.006 | 0.000 | 0.006 | 0.000 | 0.100 | 0.003 | 5930.96 | 49.53 | 0.000 | 9 |  |
| LANDRACE | LQ_1.5 | 0.895 | 0.894 | 0.000 | 0.007 | 0.000 | 0.006 | 0.000 | 0.103 | 0.003 | 5922.54 | 41.11 | 0.000 | 12 |  |
| LANDRACE | LQH_1.5 | 0.914 | 0.905 | 0.000 | 0.010 | 0.000 | 0.003 | 0.000 | 0.119 | 0.004 | 5955.92 | 74.49 | 0.000 | 66 |  |
| LANDRACE | LQHP_1.5 | 0.917 | 0.908 | 0.000 | 0.012 | 0.000 | 0.003 | 0.000 | 0.122 | 0.003 | 5972.95 | 91.52 | 0.000 | 70 |  |
| LANDRACE | L_2 | 0.893 | 0.892 | 0.000 | 0.006 | 0.000 | 0.006 | 0.000 | 0.103 | 0.003 | 5934.55 | 53.12 | 0.000 | 9 |  |
| LANDRACE | LQ_2 | 0.894 | 0.893 | 0.000 | 0.006 | 0.000 | 0.006 | 0.000 | 0.107 | 0.003 | 5922.64 | 41.21 | 0.000 | 10 |  |
| LANDRACE | LQH_2 | 0.912 | 0.904 | 0.000 | 0.009 | 0.000 | 0.003 | 0.000 | 0.119 | 0.003 | 5929.63 | 48.19 | 0.000 | 54 |  |
| LANDRACE | LQHP_2 | 0.915 | 0.907 | 0.000 | 0.011 | 0.000 | 0.003 | 0.000 | 0.113 | 0.003 | 5924.22 | 42.78 | 0.000 | 61 |  |
| LANDRACE | L_2.5 | 0.892 | 0.892 | 0.000 | 0.006 | 0.000 | 0.006 | 0.000 | 0.106 | 0.002 | 5936.26 | 54.83 | 0.000 | 8 |  |
| LANDRACE | LQ_2.5 | 0.894 | 0.893 | 0.000 | 0.006 | 0.000 | 0.006 | 0.000 | 0.103 | 0.004 | 5927.84 | 46.41 | 0.000 | 10 |  |
| LANDRACE | LQH_2.5 | 0.911 | 0.902 | 0.000 | 0.009 | 0.000 | 0.003 | 0.000 | 0.114 | 0.004 | 5936.38 | 54.95 | 0.000 | 52 |  |
| LANDRACE | LQHP_2.5 | 0.913 | 0.904 | 0.001 | 0.011 | 0.000 | 0.003 | 0.000 | 0.113 | 0.003 | 5896.26 | 14.82 | 0.000 | 47 |  |
| LANDRACE | L_3 | 0.891 | 0.891 | 0.000 | 0.006 | 0.000 | 0.003 | 0.000 | 0.110 | 0.003 | 5940.48 | 59.04 | 0.000 | 8 |  |
| LANDRACE | LQ_3 | 0.893 | 0.893 | 0.000 | 0.006 | 0.000 | 0.006 | 0.000 | 0.113 | 0.003 | 5929.73 | 48.30 | 0.000 | 9 |  |
| LANDRACE | LQH_3 | 0.909 | 0.901 | 0.000 | 0.009 | 0.000 | 0.003 | 0.000 | 0.117 | 0.004 | 5941.49 | 60.06 | 0.000 | 49 |  |
| **LANDRACE** | **LQHP_3** | **0.911** | **0.902** | **0.001** | **0.011** | **0.000** | **0.003** | **0.000** | **0.113** | **0.003** | **5881.82** | **0.39** | **0.450** | **37** | **0.642** |
| LANDRACE | L_3.5 | 0.891 | 0.891 | 0.000 | 0.006 | 0.000 | 0.003 | 0.000 | 0.110 | 0.003 | 5941.86 | 60.42 | 0.000 | 7 |  |
| LANDRACE | LQ_3.5 | 0.893 | 0.892 | 0.000 | 0.006 | 0.000 | 0.006 | 0.000 | 0.113 | 0.003 | 5933.55 | 52.12 | 0.000 | 9 |  |
| LANDRACE | LQH_3.5 | 0.908 | 0.898 | 0.000 | 0.009 | 0.000 | 0.003 | 0.000 | 0.119 | 0.004 | 5949.07 | 67.64 | 0.000 | 46 |  |
| LANDRACE | LQHP_3.5 | 0.908 | 0.901 | 0.001 | 0.011 | 0.000 | 0.003 | 0.000 | 0.113 | 0.003 | 5891.03 | 9.60 | 0.004 | 35 |  |
|  |  |  |  |  |  |  |  |  |  |  |  |  |  |  |  |
| COMMERCIAL | L_0.5 | 0.771 | 0.770 | 0.000 | 0.004 | 0.000 | 0.000 | 0.000 | 0.100 | 0.001 | 50085.99 | 1076.93 | 0.000 | 9 |  |
| COMMERCIAL | LQ_0.5 | 0.782 | 0.780 | 0.000 | 0.005 | 0.000 | 0.001 | 0.000 | 0.105 | 0.001 | 49684.41 | 675.35 | 0.000 | 15 |  |
| COMMERCIAL | LQH_0.5 | 0.827 | 0.820 | 0.000 | 0.009 | 0.000 | 0.001 | 0.000 | 0.110 | 0.000 | 49108.90 | 99.84 | 0.000 | 212 |  |
| COMMERCIAL | LQHP_0.5 | 0.830 | 0.824 | 0.000 | 0.009 | 0.000 | 0.000 | 0.000 | 0.108 | 0.000 | 49009.06 | 0.00 | 1.000 | 203 |  |
| COMMERCIAL | L_1 | 0.770 | 0.769 | 0.000 | 0.004 | 0.000 | 0.000 | 0.000 | 0.099 | 0.001 | 50101.88 | 1092.82 | 0.000 | 9 |  |
| COMMERCIAL | LQ_1 | 0.780 | 0.779 | 0.000 | 0.005 | 0.000 | 0.001 | 0.000 | 0.103 | 0.001 | 49747.27 | 738.21 | 0.000 | 13 |  |
| COMMERCIAL | LQH_1 | 0.822 | 0.817 | 0.000 | 0.008 | 0.000 | 0.001 | 0.000 | 0.102 | 0.000 | 49121.55 | 112.48 | 0.000 | 163 |  |
| **COMMERCIAL** | **LQHP_1** | **0.823** | **0.818** | **0.000** | **0.008** | **0.000** | **0.001** | **0.000** | **0.105** | **0.000** | **49044.36** | **35.30** | **0.000** | **142** | **0.608** |
| COMMERCIAL | L_1.5 | 0.769 | 0.768 | 0.000 | 0.003 | 0.000 | 0.000 | 0.000 | 0.101 | 0.001 | 50123.53 | 1114.47 | 0.000 | 8 |  |
| COMMERCIAL | LQ_1.5 | 0.778 | 0.776 | 0.000 | 0.004 | 0.000 | 0.000 | 0.000 | 0.103 | 0.000 | 49821.47 | 812.40 | 0.000 | 11 |  |
| COMMERCIAL | LQH_1.5 | 0.819 | 0.814 | 0.000 | 0.008 | 0.000 | 0.001 | 0.000 | 0.105 | 0.000 | 49124.88 | 115.81 | 0.000 | 127 |  |
| COMMERCIAL | LQHP_1.5 | 0.820 | 0.816 | 0.000 | 0.007 | 0.000 | 0.000 | 0.000 | 0.105 | 0.000 | 49071.38 | 62.32 | 0.000 | 115 |  |
| COMMERCIAL | L_2 | 0.768 | 0.767 | 0.000 | 0.003 | 0.000 | 0.000 | 0.000 | 0.098 | 0.001 | 50140.49 | 1131.43 | 0.000 | 7 |  |
| COMMERCIAL | LQ_2 | 0.775 | 0.774 | 0.000 | 0.004 | 0.000 | 0.000 | 0.000 | 0.100 | 0.000 | 49913.37 | 904.31 | 0.000 | 10 |  |
| COMMERCIAL | LQH_2 | 0.817 | 0.812 | 0.000 | 0.007 | 0.000 | 0.001 | 0.000 | 0.104 | 0.000 | 49183.64 | 174.58 | 0.000 | 121 |  |
| COMMERCIAL | LQHP_2 | 0.818 | 0.813 | 0.000 | 0.007 | 0.000 | 0.000 | 0.000 | 0.106 | 0.000 | 49116.46 | 107.39 | 0.000 | 99 |  |
| COMMERCIAL | L_2.5 | 0.766 | 0.765 | 0.000 | 0.003 | 0.000 | 0.000 | 0.000 | 0.099 | 0.001 | 50159.86 | 1150.79 | 0.000 | 7 |  |
| COMMERCIAL | LQ_2.5 | 0.772 | 0.770 | 0.000 | 0.004 | 0.000 | 0.000 | 0.000 | 0.102 | 0.000 | 50000.07 | 991.00 | 0.000 | 10 |  |
| COMMERCIAL | LQH_2.5 | 0.814 | 0.809 | 0.000 | 0.007 | 0.000 | 0.000 | 0.000 | 0.105 | 0.000 | 49224.24 | 215.18 | 0.000 | 106 |  |
| COMMERCIAL | LQHP_2.5 | 0.814 | 0.810 | 0.000 | 0.007 | 0.000 | 0.000 | 0.000 | 0.104 | 0.000 | 49167.17 | 158.11 | 0.000 | 84 |  |
| COMMERCIAL | L_3 | 0.764 | 0.763 | 0.000 | 0.003 | 0.000 | 0.000 | 0.000 | 0.103 | 0.001 | 50182.80 | 1173.73 | 0.000 | 7 |  |
| COMMERCIAL | LQ_3 | 0.770 | 0.768 | 0.000 | 0.003 | 0.000 | 0.000 | 0.000 | 0.102 | 0.000 | 50048.73 | 1039.67 | 0.000 | 9 |  |
| COMMERCIAL | LQH_3 | 0.810 | 0.806 | 0.000 | 0.006 | 0.000 | 0.001 | 0.000 | 0.106 | 0.000 | 49291.25 | 282.18 | 0.000 | 99 |  |
| COMMERCIAL | LQHP_3 | 0.811 | 0.807 | 0.000 | 0.006 | 0.000 | 0.000 | 0.000 | 0.106 | 0.000 | 49238.98 | 229.92 | 0.000 | 83 |  |
| COMMERCIAL | L_3.5 | 0.762 | 0.761 | 0.000 | 0.003 | 0.000 | 0.000 | 0.000 | 0.097 | 0.001 | 50209.33 | 1200.27 | 0.000 | 7 |  |
| COMMERCIAL | LQ_3.5 | 0.767 | 0.766 | 0.000 | 0.003 | 0.000 | 0.000 | 0.000 | 0.102 | 0.000 | 50099.65 | 1090.59 | 0.000 | 9 |  |
| COMMERCIAL | LQH_3.5 | 0.807 | 0.803 | 0.000 | 0.006 | 0.000 | 0.001 | 0.000 | 0.106 | 0.000 | 49367.76 | 358.69 | 0.000 | 103 |  |
| COMMERCIAL | LQHP_3.5 | 0.808 | 0.805 | 0.000 | 0.006 | 0.000 | 0.000 | 0.000 | 0.105 | 0.000 | 49277.56 | 268.50 | 0.000 | 69 |  |
|  |  |  |  |  |  |  |  |  |  |  |  |  |  |  |  |
| CULTIVATED | L_0.5 | 0.755 | 0.753 | 0.000 | 0.003 | 0.000 | 0.000 | 0.000 | 0.099 | 0.001 | 57084.90 | 1201.90 | 0.000 | 9 |  |
| CULTIVATED | LQ_0.5 | 0.766 | 0.764 | 0.000 | 0.003 | 0.000 | 0.000 | 0.000 | 0.102 | 0.000 | 56676.80 | 793.79 | 0.000 | 16 |  |
| CULTIVATED | LQH_0.5 | 0.812 | 0.805 | 0.000 | 0.007 | 0.000 | 0.001 | 0.000 | 0.106 | 0.000 | 55993.48 | 110.47 | 0.000 | 184 |  |
| CULTIVATED | LQHP_0.5 | 0.815 | 0.809 | 0.000 | 0.007 | 0.000 | 0.001 | 0.000 | 0.107 | 0.000 | 55883.01 | 0.00 | 1.000 | 169 |  |
| CULTIVATED | L_1 | 0.754 | 0.752 | 0.000 | 0.003 | 0.000 | 0.000 | 0.000 | 0.102 | 0.000 | 57104.05 | 1221.04 | 0.000 | 9 |  |
| CULTIVATED | LQ_1 | 0.764 | 0.763 | 0.000 | 0.003 | 0.000 | 0.000 | 0.000 | 0.103 | 0.000 | 56725.87 | 842.86 | 0.000 | 12 |  |
| CULTIVATED | LQH_1 | 0.808 | 0.802 | 0.000 | 0.006 | 0.000 | 0.001 | 0.000 | 0.105 | 0.000 | 56044.41 | 161.40 | 0.000 | 156 |  |
| **CULTIVATED** | **LQHP_1** | **0.811** | **0.805** | **0.000** | **0.006** | **0.000** | **0.001** | **0.000** | **0.109** | **0.000** | **55909.64** | **26.63** | **0.000** | **132** | **0.559** |
| CULTIVATED | L_1.5 | 0.753 | 0.751 | 0.000 | 0.003 | 0.000 | 0.000 | 0.000 | 0.101 | 0.000 | 57121.81 | 1238.80 | 0.000 | 8 |  |
| CULTIVATED | LQ_1.5 | 0.763 | 0.761 | 0.000 | 0.003 | 0.000 | 0.000 | 0.000 | 0.104 | 0.000 | 56797.60 | 914.60 | 0.000 | 12 |  |
| CULTIVATED | LQH_1.5 | 0.805 | 0.800 | 0.000 | 0.006 | 0.000 | 0.002 | 0.000 | 0.107 | 0.000 | 56090.55 | 207.54 | 0.000 | 144 |  |
| CULTIVATED | LQHP_1.5 | 0.807 | 0.803 | 0.000 | 0.005 | 0.000 | 0.000 | 0.000 | 0.104 | 0.000 | 56019.68 | 136.67 | 0.000 | 131 |  |
| CULTIVATED | L_2 | 0.751 | 0.749 | 0.000 | 0.002 | 0.000 | 0.000 | 0.000 | 0.102 | 0.000 | 57144.22 | 1261.22 | 0.000 | 8 |  |
| CULTIVATED | LQ_2 | 0.760 | 0.758 | 0.000 | 0.002 | 0.000 | 0.000 | 0.000 | 0.106 | 0.000 | 56874.19 | 991.19 | 0.000 | 10 |  |
| CULTIVATED | LQH_2 | 0.803 | 0.798 | 0.000 | 0.005 | 0.000 | 0.001 | 0.000 | 0.105 | 0.000 | 56130.84 | 247.83 | 0.000 | 129 |  |
| CULTIVATED | LQHP_2 | 0.805 | 0.800 | 0.000 | 0.005 | 0.000 | 0.000 | 0.000 | 0.103 | 0.000 | 56069.80 | 186.79 | 0.000 | 120 |  |
| CULTIVATED | L_2.5 | 0.748 | 0.747 | 0.000 | 0.002 | 0.000 | 0.000 | 0.000 | 0.102 | 0.000 | 57173.90 | 1290.89 | 0.000 | 8 |  |
| CULTIVATED | LQ_2.5 | 0.756 | 0.755 | 0.000 | 0.002 | 0.000 | 0.000 | 0.000 | 0.103 | 0.000 | 56972.38 | 1089.37 | 0.000 | 10 |  |
| CULTIVATED | LQH_2.5 | 0.800 | 0.796 | 0.000 | 0.004 | 0.000 | 0.000 | 0.000 | 0.105 | 0.000 | 56159.27 | 276.27 | 0.000 | 109 |  |
| CULTIVATED | LQHP_2.5 | 0.802 | 0.797 | 0.000 | 0.004 | 0.000 | 0.000 | 0.000 | 0.103 | 0.000 | 56105.92 | 222.92 | 0.000 | 101 |  |
| CULTIVATED | L_3 | 0.746 | 0.745 | 0.000 | 0.002 | 0.000 | 0.000 | 0.000 | 0.102 | 0.000 | 57197.26 | 1314.26 | 0.000 | 7 |  |
| CULTIVATED | LQ_3 | 0.753 | 0.752 | 0.000 | 0.002 | 0.000 | 0.000 | 0.000 | 0.103 | 0.000 | 57037.68 | 1154.68 | 0.000 | 9 |  |
| CULTIVATED | LQH_3 | 0.797 | 0.792 | 0.000 | 0.004 | 0.000 | 0.000 | 0.000 | 0.104 | 0.000 | 56251.81 | 368.81 | 0.000 | 113 |  |
| CULTIVATED | LQHP_3 | 0.799 | 0.794 | 0.000 | 0.004 | 0.000 | 0.000 | 0.000 | 0.103 | 0.000 | 56205.46 | 322.46 | 0.000 | 111 |  |
| CULTIVATED | L_3.5 | 0.745 | 0.744 | 0.000 | 0.002 | 0.000 | 0.000 | 0.000 | 0.103 | 0.000 | 57225.41 | 1342.40 | 0.000 | 7 |  |
| CULTIVATED | LQ_3.5 | 0.751 | 0.750 | 0.000 | 0.002 | 0.000 | 0.000 | 0.000 | 0.100 | 0.000 | 57075.36 | 1192.35 | 0.000 | 9 |  |
| CULTIVATED | LQH_3.5 | 0.793 | 0.789 | 0.000 | 0.003 | 0.000 | 0.000 | 0.000 | 0.103 | 0.000 | 56301.45 | 418.44 | 0.000 | 99 |  |
| CULTIVATED | LQHP_3.5 | 0.796 | 0.791 | 0.000 | 0.004 | 0.000 | 0.000 | 0.000 | 0.101 | 0.000 | 56229.29 | 346.28 | 0.000 | 84 |  |
