# Supplementary material for: Fluctuation of ecological niches and geographic range shifts along chile pepper's domestication gradient
Source: Ecol Evol. 2023 Nov 28;13(11):e10731. doi: 10.1002/ece3.10731 (PMC10682905; doi:10.1002/ece3.10731)
Supplement: Supplementary file 1 — Appendix S1 [file ECE3-13-e10731-s001.zip › Appendix1_SuppTable_SA4.docx]

**Supplementary Appendix table SA4**

| category | pixels |
| --- | --- |
| **SEMIWILD-COMMERCIAL** |  |
| none | 305216 |
| only_SEMIWILD | 46525 |
| only_COMMERCIAL | 39750 |
| overlap | 25618 |
| percent | 37.26 |
| **WILD-LANDRACE** |  |
| none | 337510 |
| only_LANDRACE | 6368 |
| only_WILD | 32503 |
| overlap | 40728 |
| percent | 67.70 |
| **WILD_SL-CULTIVATED** |  |
| none | 310630 |
| only_CULTIVATED | 33115 |
| only_WILDsl | 35069 |
| overlap | 38295 |
| percent | 52.90 |
| **WILD-COMMERCIAL** |  |
| none | 311218 |
| only_COMMERCIAL | 32660 |
| only_WILD | 40523 |
| overlap | 32708 |
| percent | 47.20 |
| **LANDRACE-COMMERCIAL** |  |
| none | 324775 |
| only_COMMERCIAL | 45238 |
| only_LANDRACE | 26966 |
| overlap | 20130 |
| percent | 35.80 |
| **WILD-SEMIWILD** |  |
| none | 325209 |
| only_SEMIWILD | 18669 |
| only_WILD | 19757 |
| overlap | 53474 |
| percent | 73.57 |
| **SEMIWILD-LANDRACE** |  |
| none | 342193 |
| only_SEMIWILD | 27820 |
| only_LANDRACE | 2773 |
| overlap | 44323 |
| percent | 74.34 |
