# Supplementary material for: Fluctuation of ecological niches and geographic range shifts along chile pepper's domestication gradient
Source: Ecol Evol. 2023 Nov 28;13(11):e10731. doi: 10.1002/ece3.10731 (PMC10682905; doi:10.1002/ece3.10731)
Supplement: Supplementary file 1 — Appendix S1 [file ECE3-13-e10731-s001.zip › SuppFig_S4.pdf]

Supp. figure S4

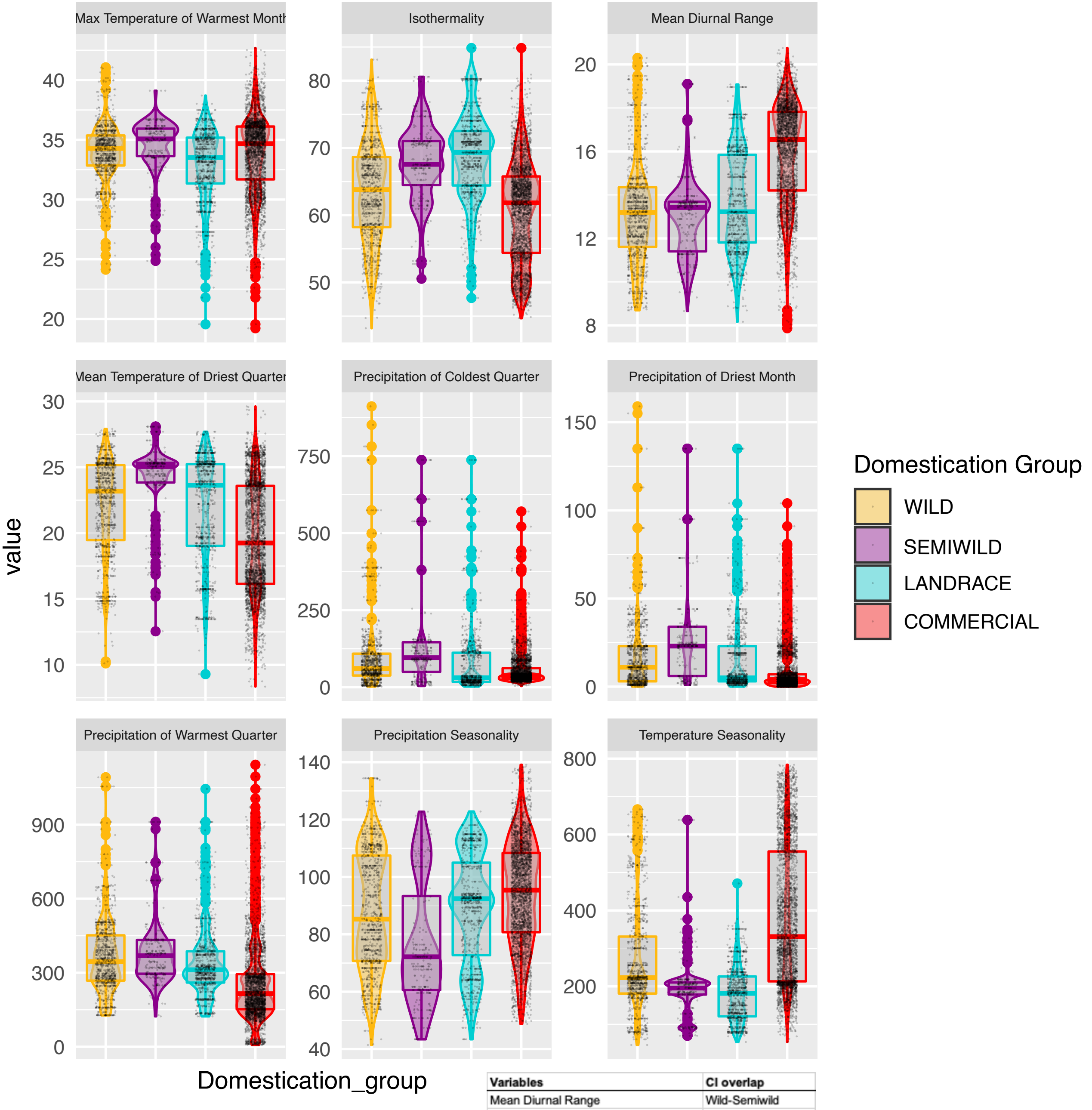

Variables	CI overlap
Mean Diurnal Range	Wild-Semiwild
Isothermality	Semiwild-Landrace
Temperature Seasonality	--
Max Temperature of Warmest Month	Wild-Commercial
Mean Temperature of Driest Quarter	Wild-Landrace
Precipitation of Driest Month	Wild-Landrace
Precipitation Seasonality	Wild-Landrace
Precipitation of Warmest Quarter	Wild-Semiwild
Precipitation of Coldest Quarter	--
