# Supplementary material for: Fluctuation of ecological niches and geographic range shifts along chile pepper's domestication gradient
Source: Ecol Evol. 2023 Nov 28;13(11):e10731. doi: 10.1002/ece3.10731 (PMC10682905; doi:10.1002/ece3.10731)
Supplement: Supplementary file 1 — Appendix S1 [file ECE3-13-e10731-s001.zip › SuppFig_S5.pdf]

Supp. figure S5

a

median

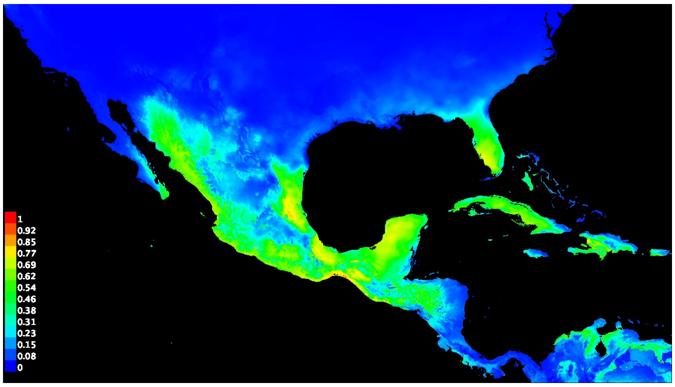

WILD

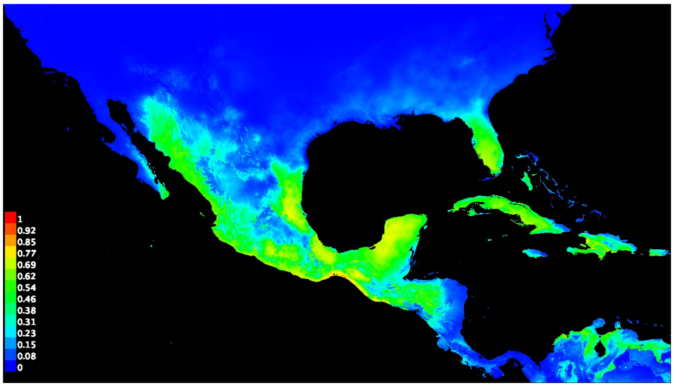

WILD_SL

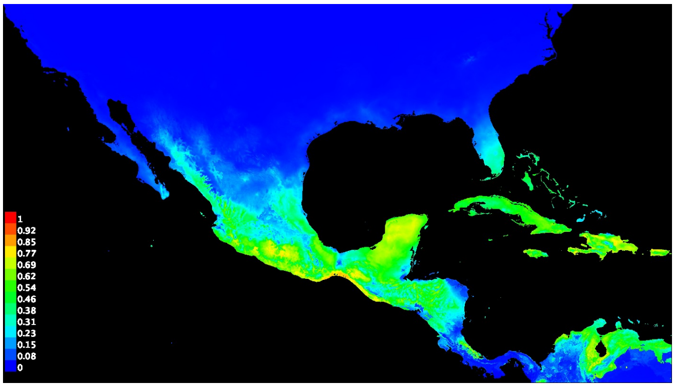

SEMIWILD

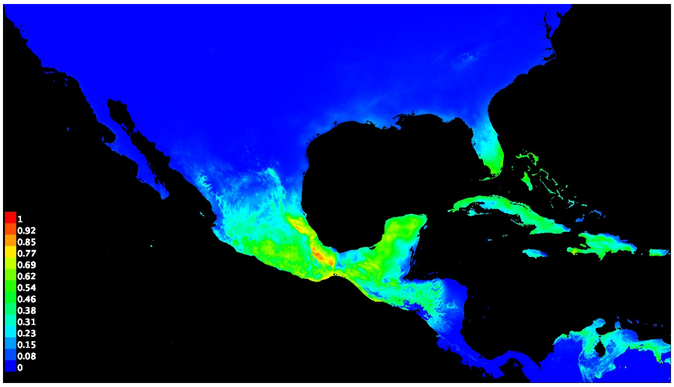

LANDRACE

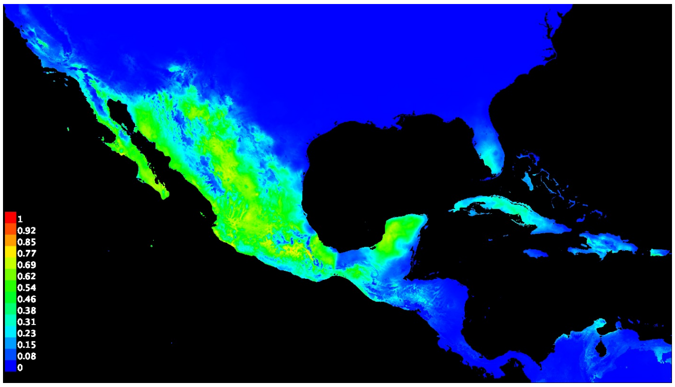

COMMERCIAL

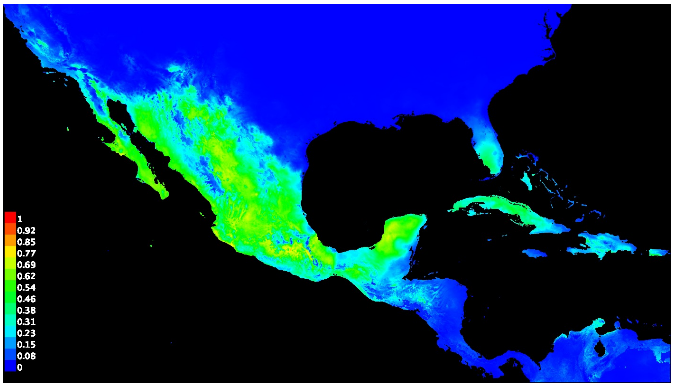

CULTIVATED

b

binary

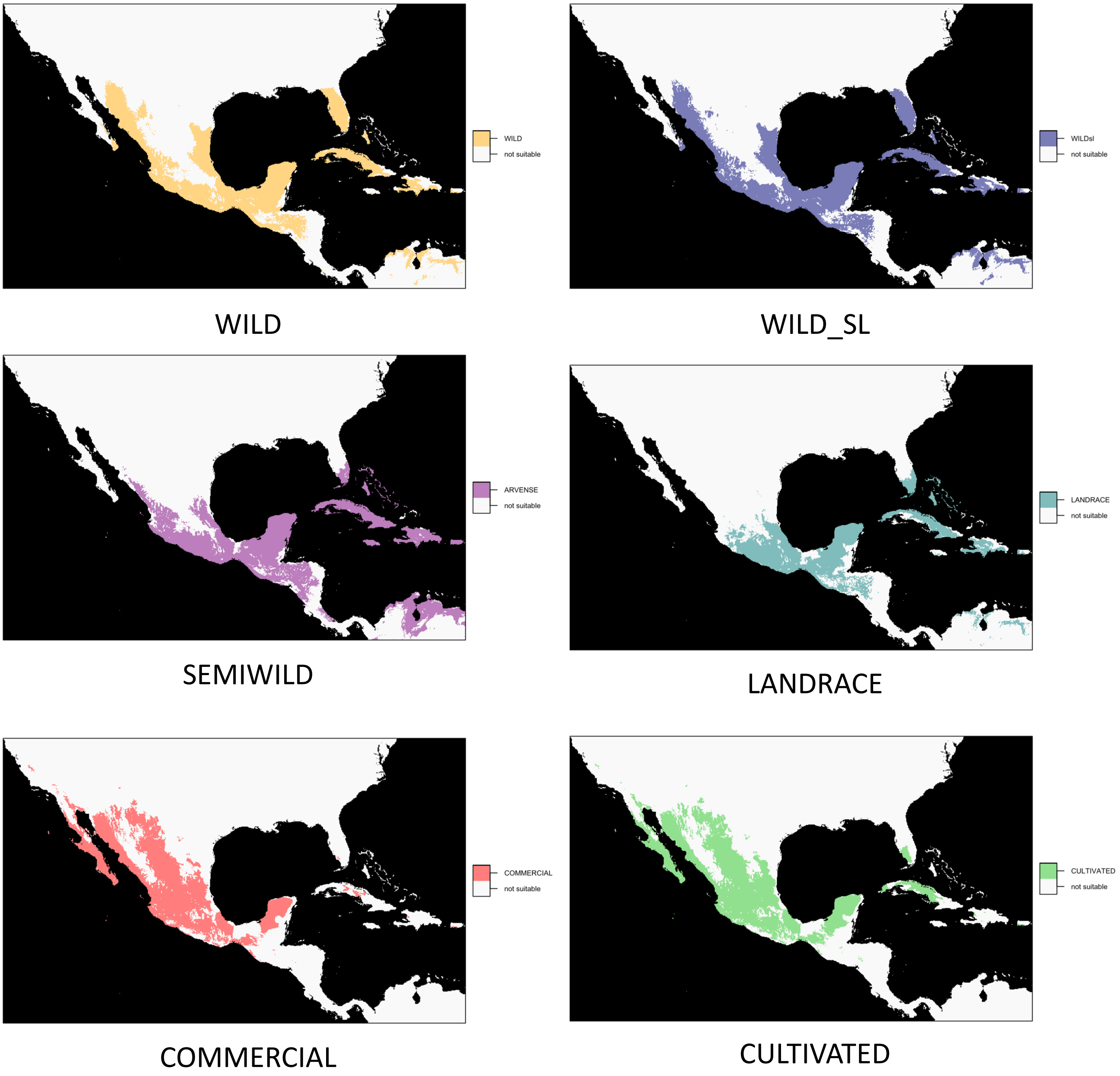
WILD
not suitable
WILD
WILDsl
not suitable
WILD_SL
ARVENSE
not suitable
SEMIWILD
LANDRACE
not suitable
LANDRACE
COMMERCIAL
not suitable
COMMERCIAL
CULTIVATED
not suitable
CULTIVATED

C

binary paired

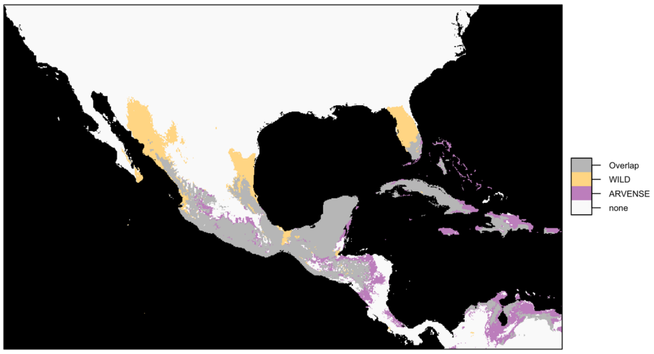

WILD-SEMIWILD

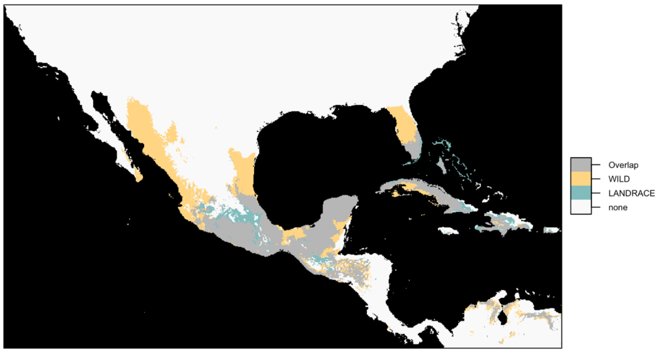

WILD-LANDRACE

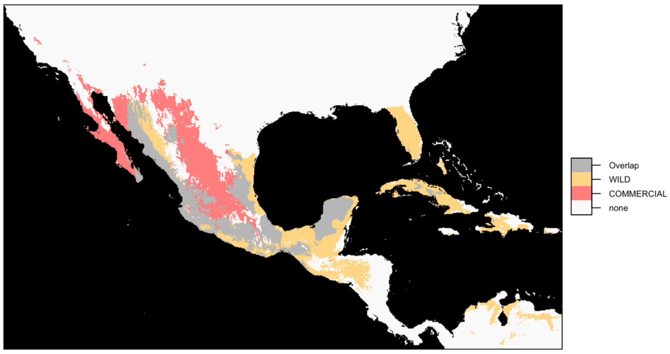

WILD-COMMERCIAL

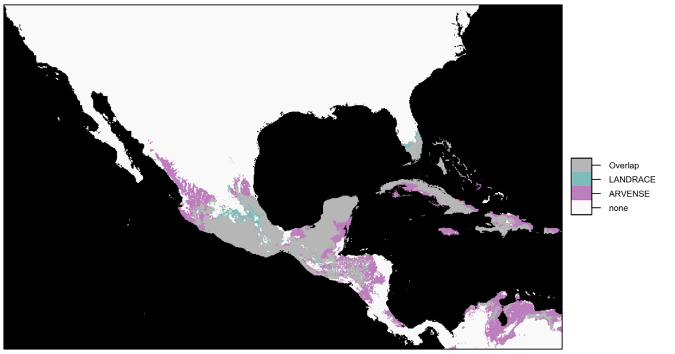

SEMIWILD-LANDRACE

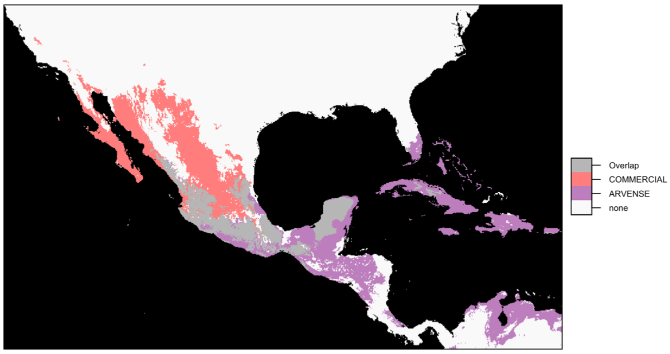

SEMIWILD-COMMERCIAL

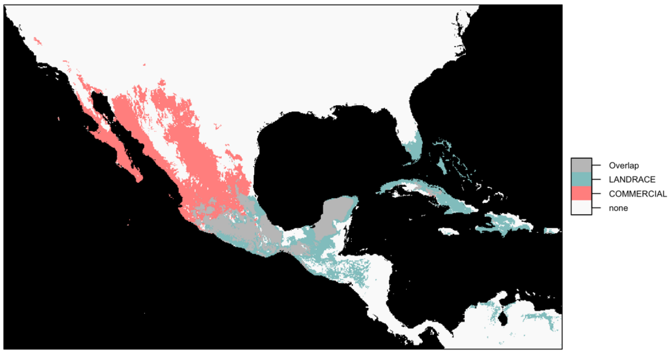

LANDRACE-COMMERICAL

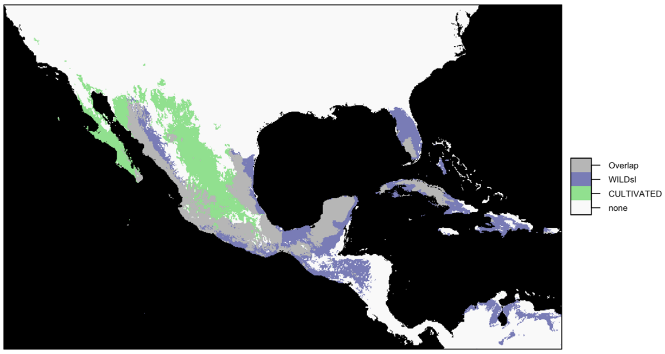

WILD_SL-CULTIVATED
