# Supplementary material for: Fluctuation of ecological niches and geographic range shifts along chile pepper's domestication gradient
Source: Ecol Evol. 2023 Nov 28;13(11):e10731. doi: 10.1002/ece3.10731 (PMC10682905; doi:10.1002/ece3.10731)
Supplement: Supplementary file 1 — Appendix S1 [file ECE3-13-e10731-s001.zip › SuppFig_S9.pdf]

Supp. figure S9

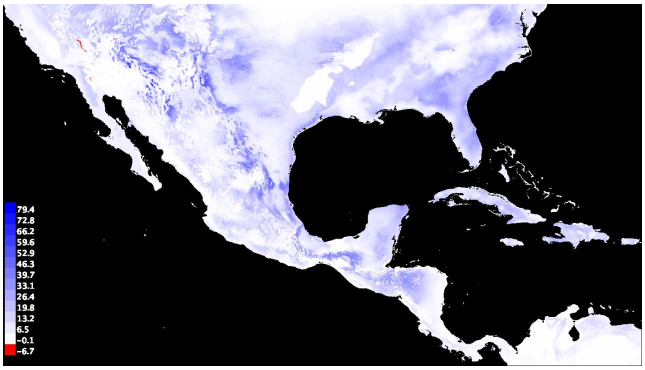

BCC-CSM2-MR

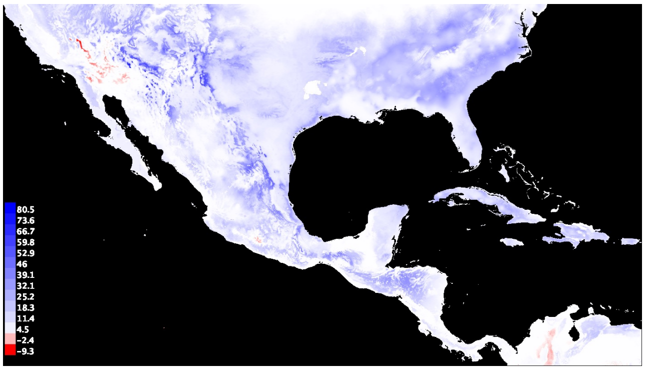

CanESM5

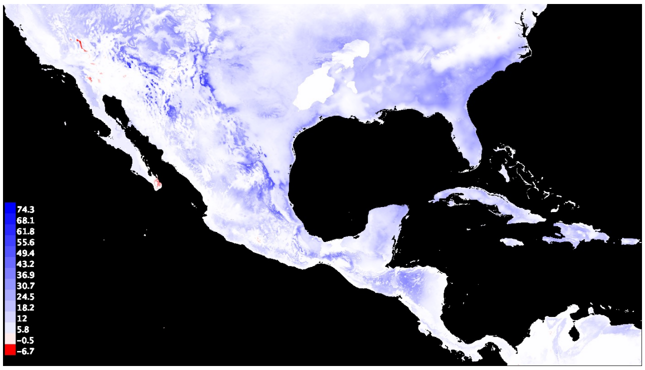

CNRM-CM6-1

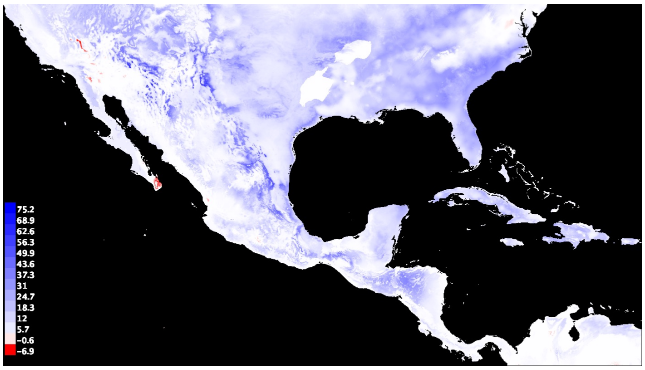

CNRM-ESM2-1

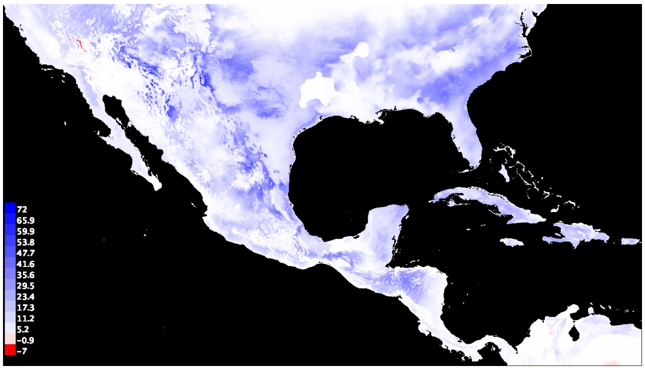

IPSL-CM6A-LR

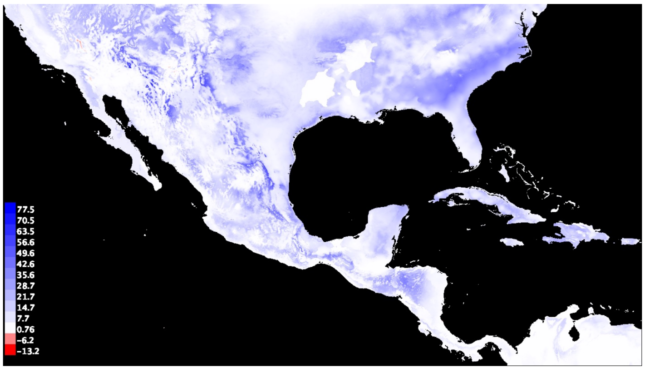

MIROC-ES2L

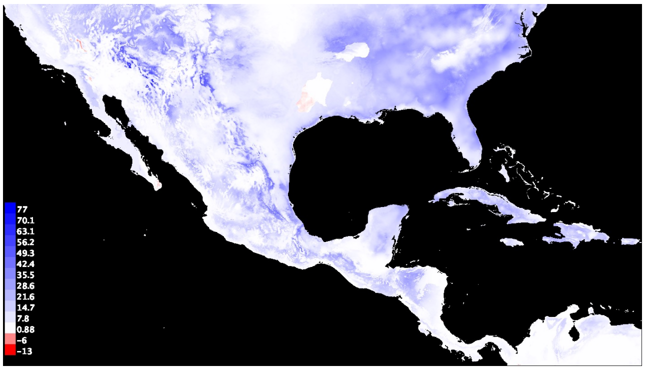

MIROC6

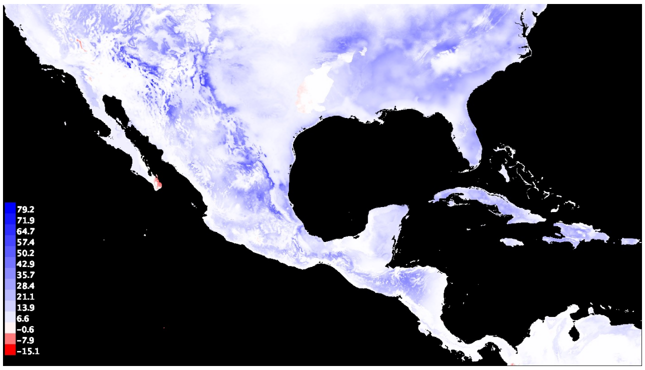

MRI-ESM2-0

2050 SSP=2_45

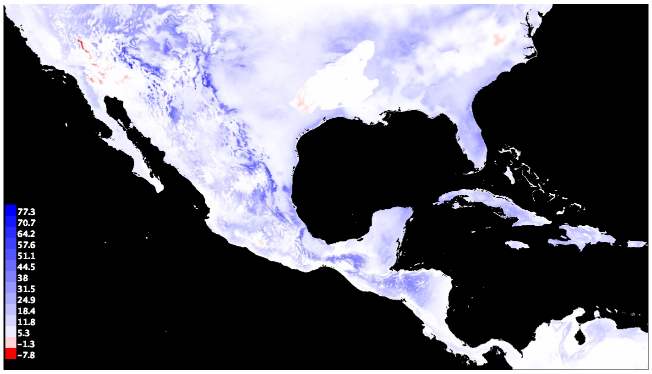

BCC-CSM2-MR

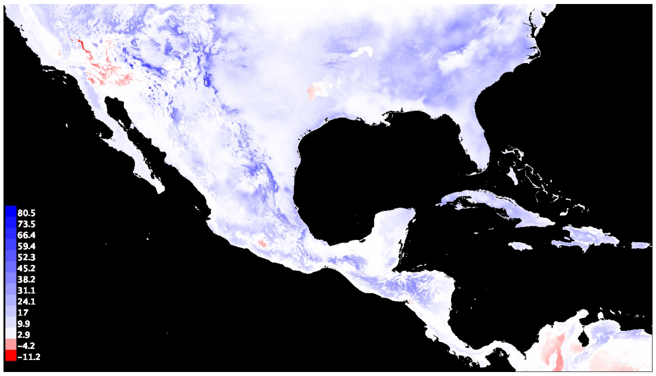

CanESM5

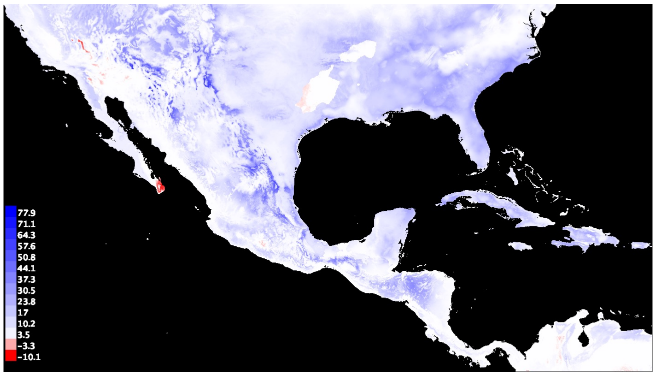

CNRM-CM6-1

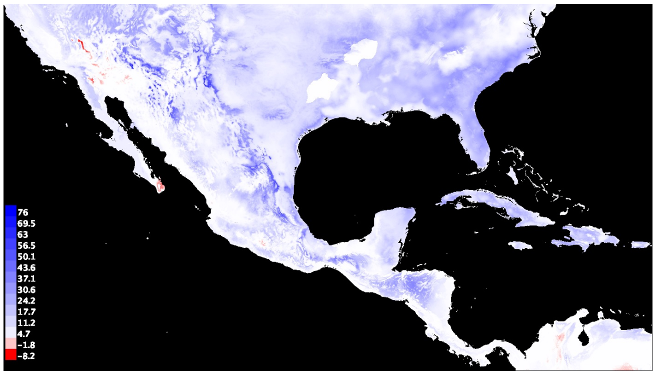

CNRM-ESM2-1

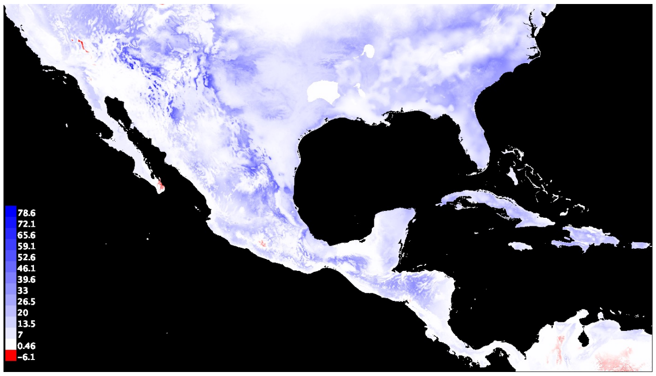

IPSL-CM6A-LR

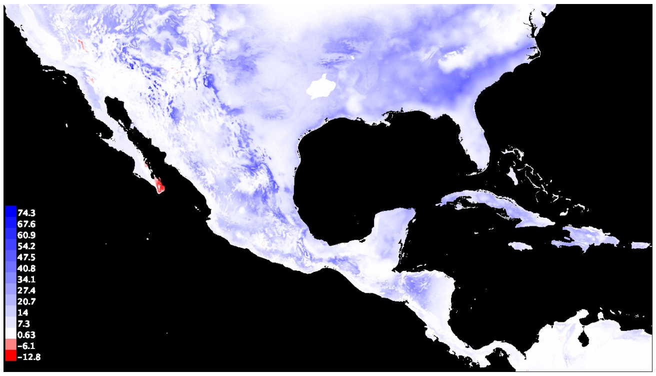

MIROC-ES2L

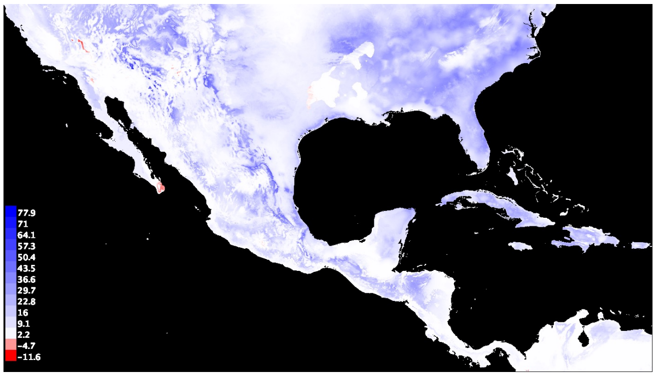

MIROC6

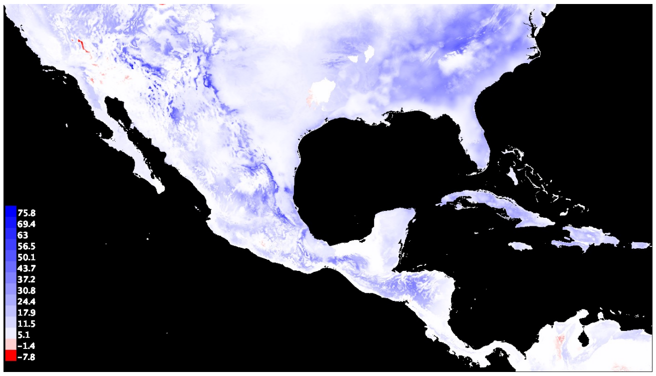

MRI-ESM2-0

2050 SSP=5_85

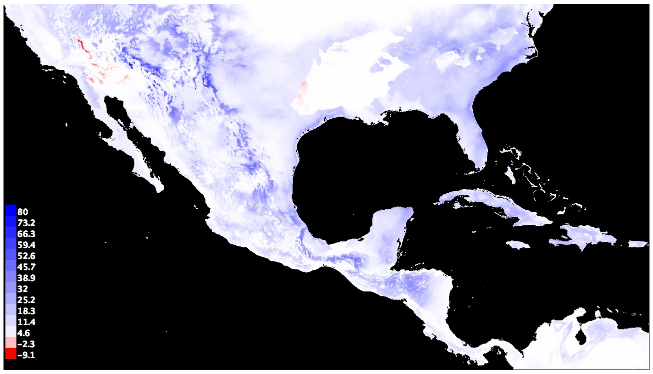

BCC-CSM2-MR

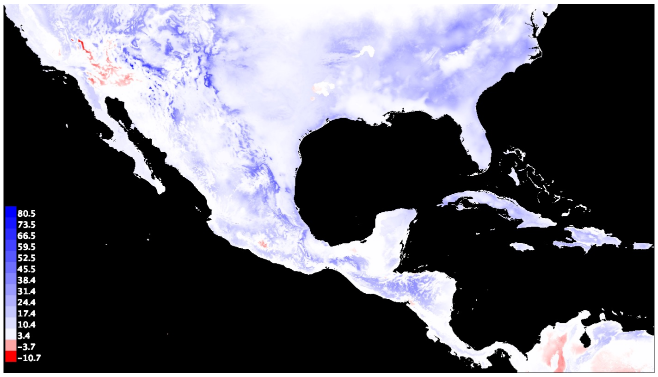

CanESM5

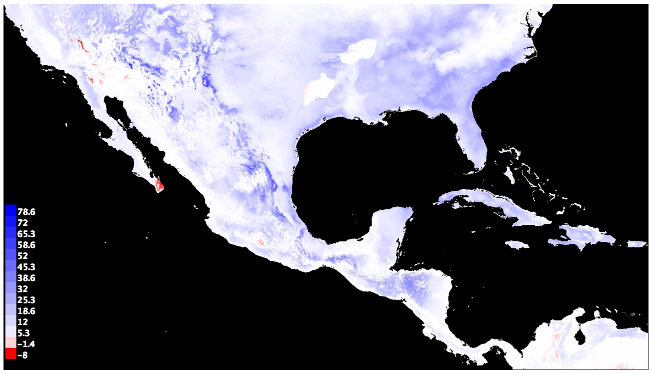

CNRM-CM6-1

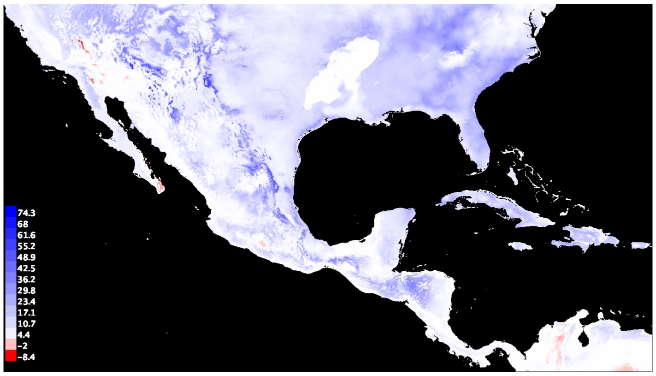

CNRM-ESM2-1

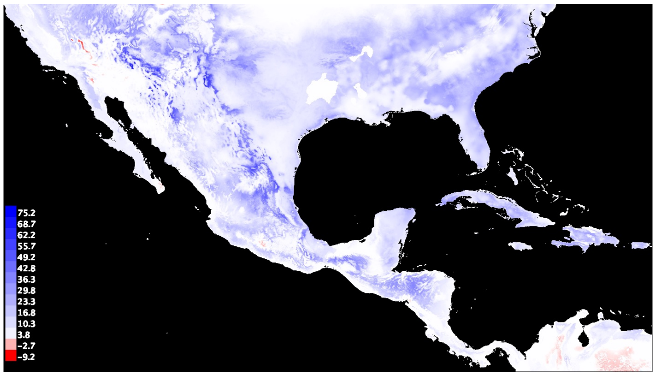

IPSL-CM6A-LR

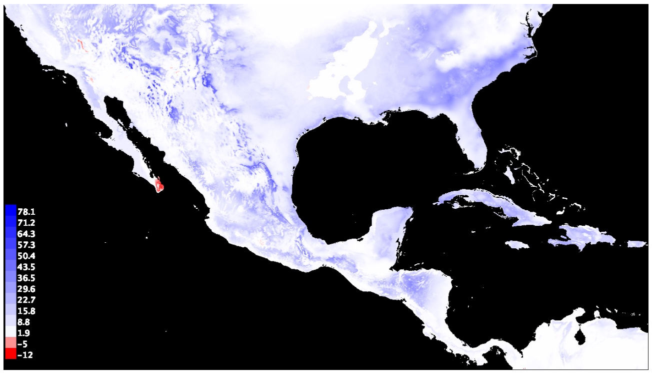

MIROC-ES2L

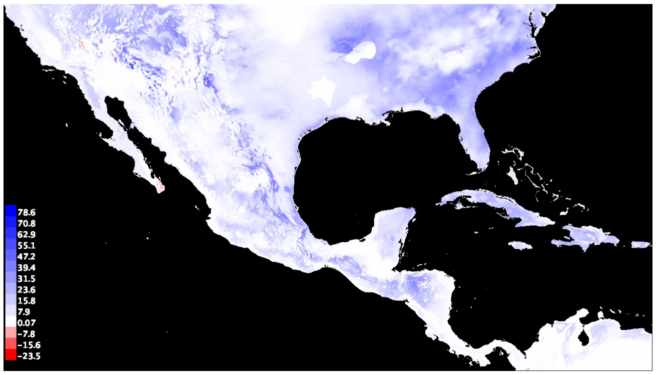

MIROC6

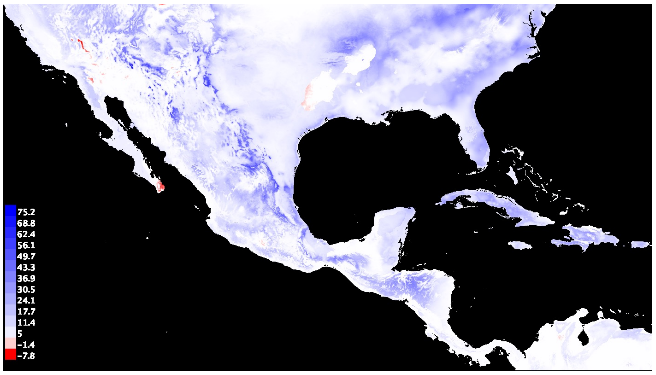

MRI-ESM2-0

2070 SSP=2_45

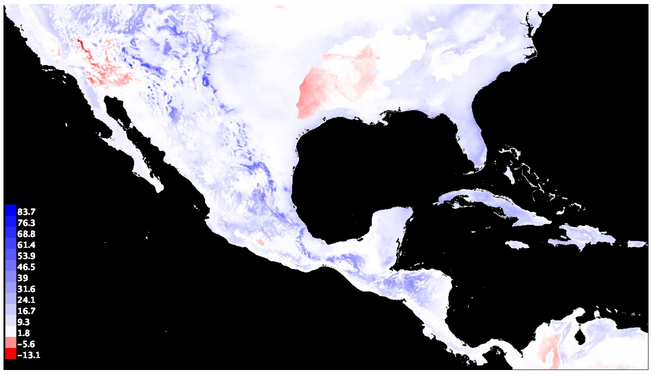

BCC-CSM2-MR

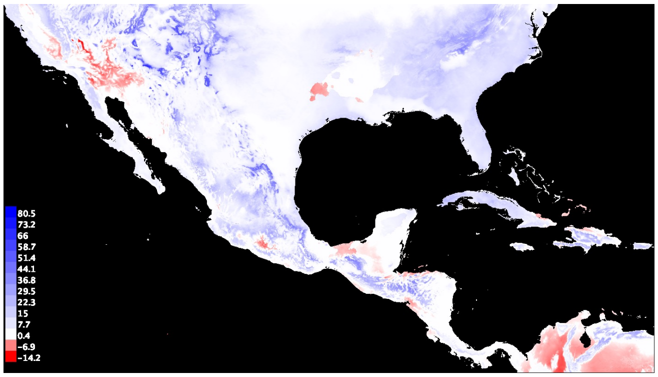

CanESM5

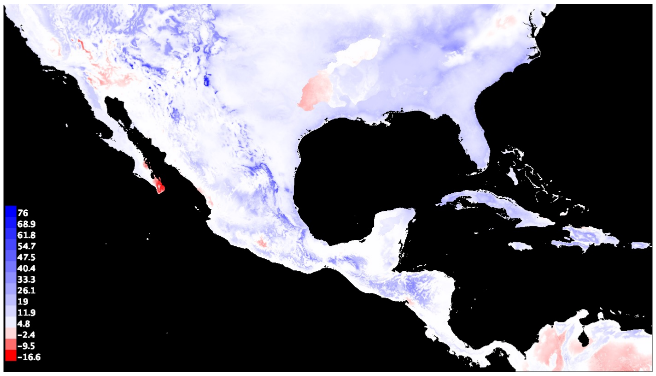

CNRM-CM6-1

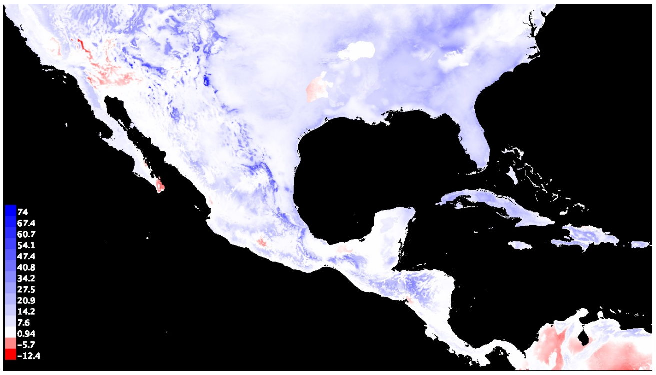

CNRM-ESM2-1

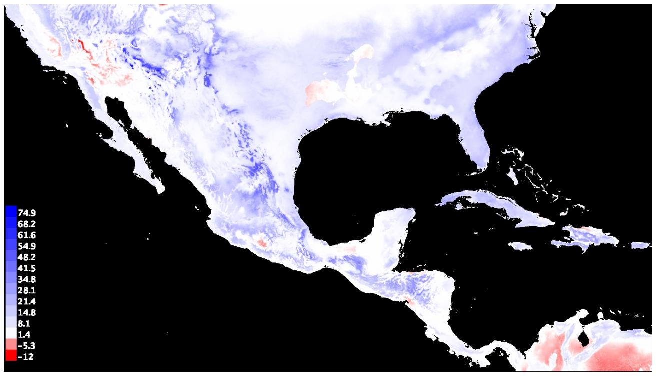

IPSL-CM6A-LR

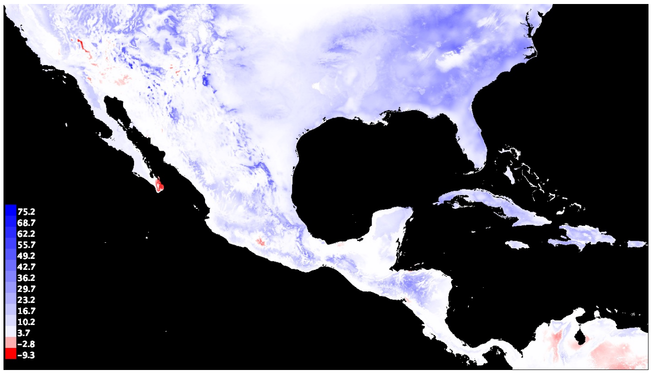

MIROC-ES2L

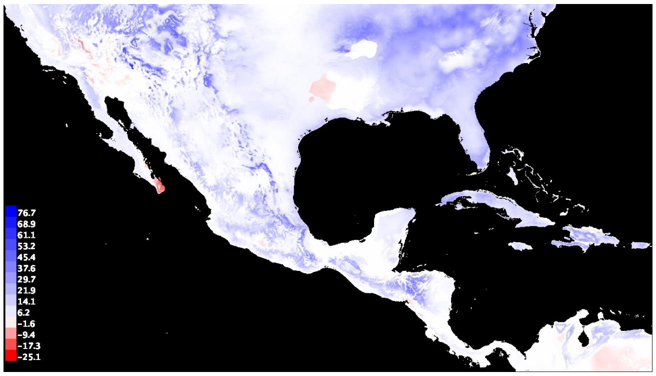

MIROC6

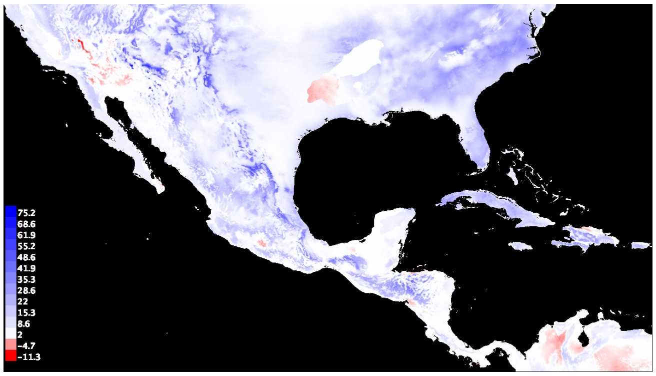

MRI-ESM2-0

2070 SSP=5_85

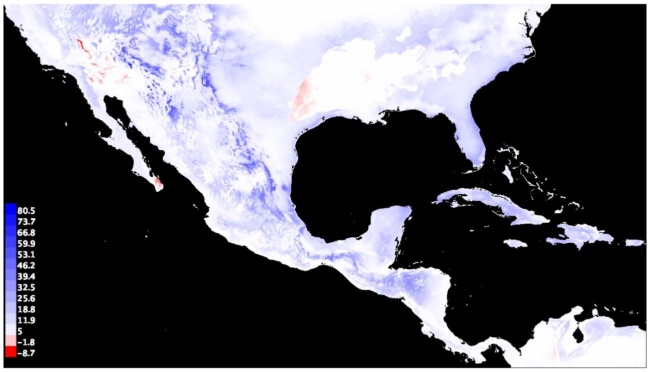

BCC-CSM2-MR

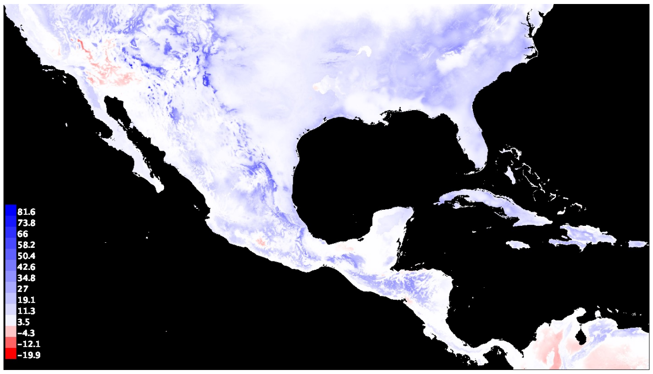

CanESM5

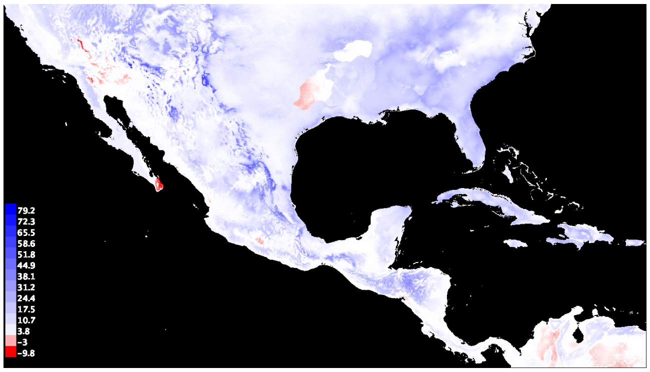

CNRM-CM6-1

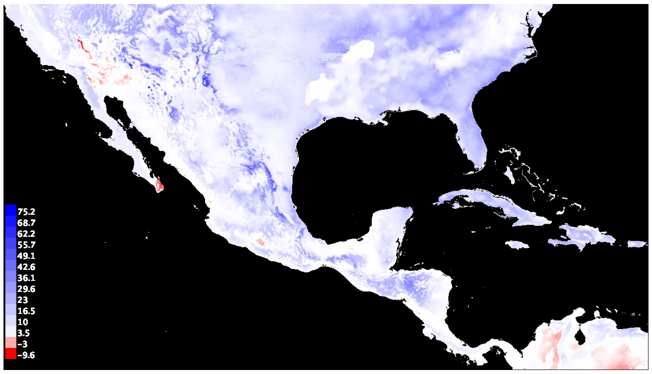

CNRM-ESM2-1

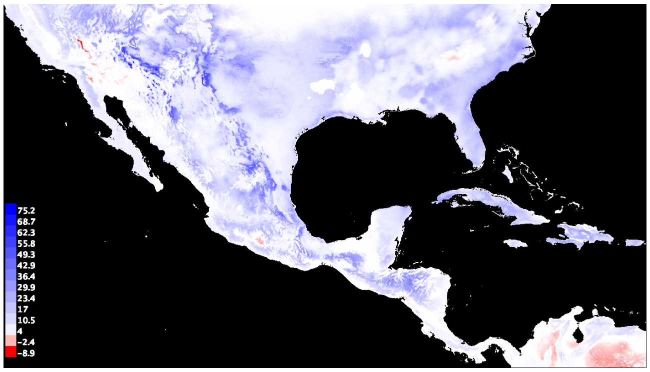

IPSL-CM6A-LR

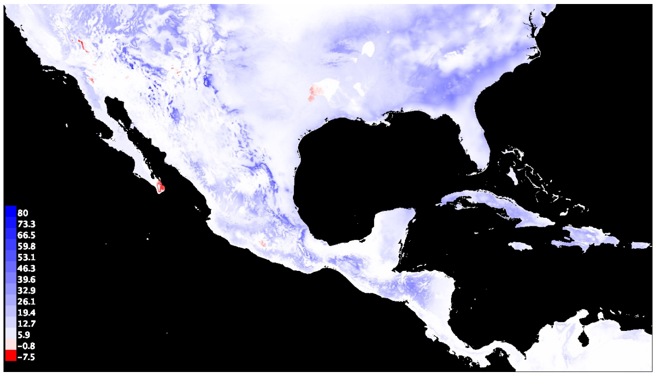

MIROC-ES2L

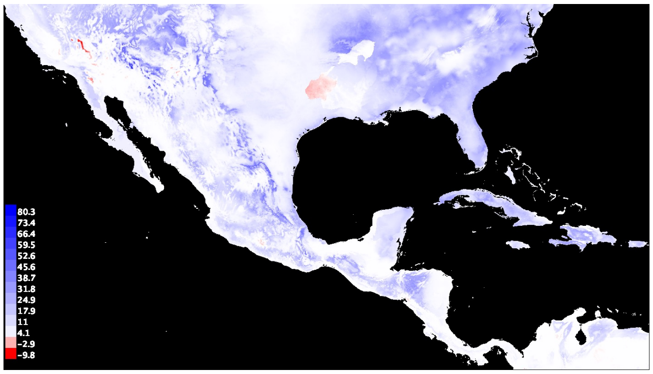

MIROC6

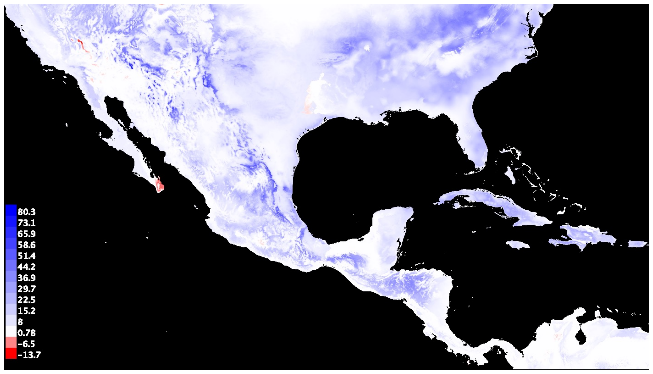

MRI-ESM2-0

2090 SSP=2_45

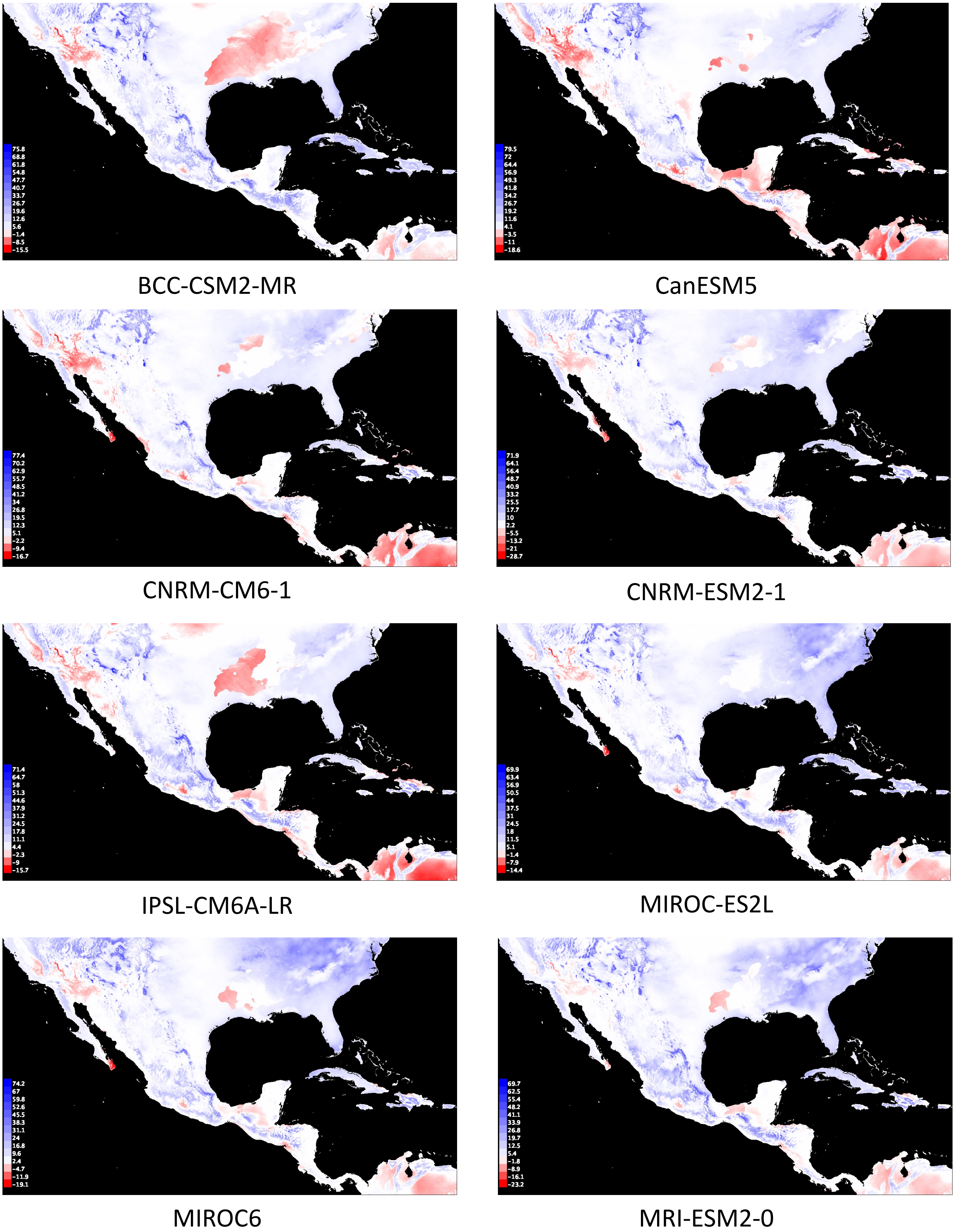
BCC-CSM2-MR
CanESM5
CNRM-CM6-1
CNRM-ESM2-1
IPSL-CM6A-LR
MIROC-ES2L
MIROC6
MRI-ESM2-0

2090 SSP=5_85
