# Supplementary material for: Fluctuation of ecological niches and geographic range shifts along chile pepper's domestication gradient
Source: Ecol Evol. 2023 Nov 28;13(11):e10731. doi: 10.1002/ece3.10731 (PMC10682905; doi:10.1002/ece3.10731)
Supplement: Supplementary file 1 — Appendix S1 [file ECE3-13-e10731-s001.zip › Supplementary Figures Index.docx]

##########################

### Supplementary Figures ###

##########################

**# SuppFig_1**

Maps of Mexico for each *C. annuum* domestication class showing the data point occurrences included in this study (thin1).

**# SuppFig_2**

*C. annuum* Mexico distribution PCA projection plots on first two principal components, showing pairwise combinations of stepwise domestication classes.

**# SuppFig_3**

Mexico maps for pairwise combinations of stepwise domestication classes depicting data points exclusive and shared to each class according to the convex hull areas plotted in the corresponding PCAs. Pairs plotted are: semiwild *vs* wild (a), semiwild *vs* landrace (b), landrace *vs* wild (c), commercial *vs* wild (d), semiwild *vs* commercial (e) and commercial *vs* landrace (f). Empty circles indicate occurrences found exclusively in one of the classes’ hull, filled circles indicate occurrences in the overlapping portion of the hulls.

**# SuppFig_4**

Violin plots of each variable’s dispersion included in *C. annuum* PCAs by domestication class in Mexico.

**# SuppFig_5**

*C. annuum* median Maxent logistic projection output over 10 replicate runs per domestication class in a Mexico-centered rectangle (a). Binary maps with unique thresholds for each domestication class, numbers in parenthesis indicate mean suitability values in withheld and non-withheld data points during modeling (b). Paired domestication classes binary plots showing overlap area (c). Standard deviation maps of the 10 replicate runs per domestication class (d).

**# SuppFig_6**

Stacked bar plots showing permutation importance of each variable included in *C. annuum* maxent niche modeling per domestication class.

**# SuppFig_7**

*C. annuum* background test histograms comparing niche similarity values for D and I in comparison pairs of wilds vs commercials and wilds vs landraces.

**# SuppFig_8**

Boxplots depicting pixels lost (a), kept (b) and new (c) for each *C.annuum* domestication class, according to area differences between present maxent niche model geographical projections and their future projections under three years and two SSP pathways associated to rcp 45 and 85.

**# SuppFig_9**

Multivariate Environmental Similarity Surfaces for each year-SSP-GCM Maxent model combinations.

**# SuppFig_10**

Multivariate Environmental Similarity Surfaces found to diverge in *C. annuum* Maxent models for landraces in a Mexico-centered rectangle.

#########################

### Supplementary Tables ###

#########################

**# SuppTable_1**

*Capsicum annuum* chile pepper occurrence points throughout Mexico labeled by domestication class. These data are the result of thinning at 5km.

**# SuppTable_2**

*C. annuum* Mexican occurrences PCA variable statistics: contributions, coordinates and loadings of each variable to the first five principal components.

**# SuppTable_3**

Overlap calculations for pairwise domestication classes minimum convex hulls. Top table marks the percentage of points found in the overlapping hull area on first two PC per pair, and the percentage of points according to each of the classes in the pair. Bottom table describes hull areas and volumes per domestication class and their paired intersections, (percentages shown for area overlaps only).

**# SuppTable_4**

Pixel occupancy per year-SSP combinations for each pairwise domestication class comparison and their overlap as area and percentage.

**# SuppTable_5**

Variable contributions to maxent models for each domestication class and Jackknife calculations for training points, test points and AUC when excluding the variable and when including exclusively the variable.

**# SuppTable_6**

Niche similarity measurements: Schoener’sD, Hellinger’s I (normalized by Warren) and Spearman rank correlation, for each pairwise domestication class comparison.

**# SuppTable_7**

Quantification of the area lost, kept and new, into future scenarios. Pixel occupancy comparing present model projections to per year-SSP combinations for each domestication class. Year median values as well as model intersections are also shown.

**# SuppTable_8**

Chile pepper landrace occurrences that are predicted to no longer occupy suitable habitat in future climate change scenarios.

###############

### Appendix ###

###############

**Appendix 1**

##################################

### Appendix Supplementary Figures ###

##################################

**# Supp Fig_A1**

Factor map for PCA analyses of all Mexican *C. annuum* samples, including soil and climate variables. The associated uncertainty per variable due to missing values imputation is shown as colored point deviations.

**# SuppFig_A2**

Correlation plots for climatic variables calculated for the whole Mexican *C. annuum* dataset, broad domestication categories (wild *sl* and cultivated) and stepwise domestication categories (wild, semiwild, landrace and commercial).

**# SuppFig_A3**

Effect of varying combinations of feature class and regularization multiplier values on average test AUC, AICc and OR for Mexican *C. annuum* maxent modeling tunning.

**# SuppFigs_A4**

Future projections of niche modeling per *C. annuum* domestication class for a Mexico-centered rectangle. For each class and year-ssp combination, all GCMs are plotted showing gradual shading when overlap occurs.

**# SuppFig_A5**

Future projections of niche modeling per *C. annuum* domestication class for a Mexico-centered rectangle, displaying the overlap of each class with respect to its present-day projection.

**# SuppFig_A6**

Future projections of niche modeling per *C. annuum* domestication class for a Mexico-centered rectangle, displaying the overlap among pairwise classes.

#################################

### Appendix Supplementary Tables ###

#################################

**# SuppTable_A1**

Chile pepper PCA variable contributions, scores and loadings for whole dataset including bioclim variables, soil variables obtained from ISRIC layers 2017(11 physical and 6 chemical), slope and aspect.

**# SuppTable_A2**

Maxent model running tests on whole C. annum Mexican dataset. Area under the curve (AUC), Akaike information criterion (AIC) and omission rate (OR) per domestication class under combinations of beta multiplier parameter (0.5, 1, 1.5, 2, 2.5, 3 and 3.5) and feature class: linear (L), quadratic (Q), hinge (H), product (P). TSS scores are shown for the selected parameter combinations in bold.

**# SuppTable_A3**

Additional Central American *C. annuum* occurrence points added to background tests (data shown without thinning) obtained from Kraft et al., (2014) and Khoury et al., (2019).

**# SuppTable_A4**

Pixel occupancy of present model projections for pairwise domestication class comparisons, showing area appointed to the overlapping area as well as exclusive to each class.
