# Supplementary material for: Fluctuation of ecological niches and geographic range shifts along chile pepper's domestication gradient
Source: Ecol Evol. 2023 Nov 28;13(11):e10731. doi: 10.1002/ece3.10731 (PMC10682905; doi:10.1002/ece3.10731)
Supplement: Supplementary file 1 — Appendix S1 [file ECE3-13-e10731-s001.zip › SuppTable_S2.docx]

**Supplementary table 2**

| **Variables Contribution** | | |  |  |  |
| --- | --- | --- | --- | --- | --- |
|  | Dim.1 | Dim.2 | Dim.3 | Dim.4 | Dim.5 |
| BIO2 | 18.8 | 0.8 | 0.1 | 0.6 | 65.8 |
| BIO3 | 7.4 | 26.8 | 0.3 | 8.8 | 16.2 |
| BIO4 | 13.6 | 19.1 | 2.1 | 3.8 | 0.6 |
| BIO5 | 0.0 | 5.0 | 53.7 | 2.7 | 2.8 |
| BIO9 | 10.9 | 0.8 | 33.6 | 3.4 | 0.6 |
| BIO14 | 16.3 | 13.8 | 2.6 | 0.0 | 2.4 |
| BIO15 | 4.8 | 16.8 | 6.7 | 36.8 | 0.0 |
| BIO18 | 14.8 | 0.2 | 0.3 | 41.8 | 6.8 |
| BIO19 | 13.3 | 16.7 | 0.6 | 2.1 | 4.8 |
|  |  |  |  |  |  |
|  |  |  |  |  |  |
| **Variables Coordinates** | | |  |  |  |
|  | Dim.1 | Dim.2 | Dim.3 | Dim.4 | Dim.5 |
| BIO2 | -0.83 | 0.13 | -0.03 | -0.07 | 0.53 |
| BIO3 | 0.52 | -0.75 | -0.06 | -0.27 | 0.26 |
| BIO4 | -0.71 | 0.63 | 0.18 | 0.17 | -0.05 |
| BIO5 | -0.02 | 0.32 | 0.91 | -0.15 | 0.11 |
| BIO9 | 0.63 | -0.13 | 0.72 | -0.17 | -0.05 |
| BIO14 | 0.77 | 0.54 | -0.20 | 0.02 | 0.10 |
| BIO15 | -0.42 | -0.60 | 0.32 | 0.54 | 0.01 |
| BIO18 | 0.74 | -0.07 | 0.07 | 0.58 | 0.17 |
| BIO19 | 0.70 | 0.59 | -0.10 | 0.13 | 0.14 |
|  |  |  |  |  |  |
|  |  |  |  |  |  |
| **Variables Loadings** | | |  |  |  |
|  | Dim.1 | Dim.2 | Dim.3 | Dim.4 | Dim.5 |
| BIO2 | -0.43 | 0.09 | -0.03 | -0.08 | 0.81 |
| BIO3 | 0.27 | -0.52 | -0.05 | -0.30 | 0.40 |
| BIO4 | -0.37 | 0.44 | 0.15 | 0.19 | -0.08 |
| BIO5 | -0.01 | 0.22 | 0.73 | -0.16 | 0.17 |
| BIO9 | 0.33 | -0.09 | 0.58 | -0.18 | -0.08 |
| BIO14 | 0.40 | 0.37 | -0.16 | 0.02 | 0.15 |
| BIO15 | -0.22 | -0.41 | 0.26 | 0.61 | 0.02 |
| BIO18 | 0.39 | -0.04 | 0.06 | 0.65 | 0.26 |
| BIO19 | 0.37 | 0.41 | -0.08 | 0.14 | 0.22 |
