# Supplementary material for: Fluctuation of ecological niches and geographic range shifts along chile pepper's domestication gradient
Source: Ecol Evol. 2023 Nov 28;13(11):e10731. doi: 10.1002/ece3.10731 (PMC10682905; doi:10.1002/ece3.10731)
Supplement: Supplementary file 1 — Appendix S1 [file ECE3-13-e10731-s001.zip › SuppTable_S3.docx]

**Supplementary table 3**

| **WILD-SEMIWILD** |  |
| --- | --- |
| percentage points overlap | 0.91 |
| SEMIWILD percentage points overlap | 0.99 |
| WILD percentage points overlap | 0.89 |
| **WILD-LANDRACE** |  |
| percentage points overlap | 0.93 |
| LANDRACE percentage points overlap | 0.99 |
| WILD percentage points overlap | 0.89 |
| **WILD-COMMERCIAL** |  |
| percentage points overlap | 0.82 |
| COMMERCIAL percentage points overlap | 0.78 |
| WILD percentage points overlap | 0.98 |
| **LANDRACE-COMMERCIAL** |  |
| percentage points overlap | 0.72 |
| COMMERCIAL percentage points overlap | 0.65 |
| LANDRACE percentage points overlap | 0.99 |
| **SEMIWILD-LANDRACE** |  |
| percentage points overlap | 0.90 |
| SEMIWILD percentage points overlap | 0.98 |
| LANDRACE percentage points overlap | 0.88 |
| **SEMIWILD-COMMERCIAL** |  |
| percentage points overlap | 0.52 |
| SEMIWILD percentage points overlap | 0.96 |
| COMMERCIAL percentage points overlap | 0.48 |
|  |  |
|  |  |
| **WILD-SEMIWILD** |  |
| SEMIWILD hull area | 142.94 |
| WILD hull area | 214.65 |
| intersection of hulls area | 142.08 |
| SEMIWILD hull volume | 104.92 |
| WILD hull volume | 207.45 |
| intersection of hulls volume | 104.39 |
| percentage area overlap | 0.79 |
| SEMIWILDpercentage area overlap | 0.99 |
| WILDpercentage area overlap | 0.66 |
|  |  |
| **WILD-LANDRACE** |  |
| LANDRACE hull area | 163.81 |
| WILD hull area | 214.65 |
| intersection of hulls area | 144.31 |
| LANDRACE hull volume | 121.77 |
| WILD hull volume | 207.45 |
| intersection of hulls volume | 109.21 |
| percentage area overlap | 0.76 |
| LANDRACEpercentage area overlap | 0.88 |
| WILDpercentage area overlap | 0.67 |
|  |  |
| **WILD-COMMERCIAL** |  |
| COMMERCIAL hull area | 202.49 |
| WILD hull area | 214.65 |
| intersection of hulls area | 162.46 |
| COMMERCIAL hull volume | 204.22 |
| WILD hull volume | 207.45 |
| intersection of hulls volume | 154.42 |
| percentage area overlap | 0.78 |
| COMMERCIALpercentage area overlap | 0.80 |
| WILDpercentage area overlap | 0.76 |
|  |  |
| **LANDRACE-COMMERCIAL** |  |
| COMMERCIAL hull area | 202.49 |
| LANDRACE hull area | 163.81 |
| intersection of hulls area | 141.98 |
| COMMERCIAL hull volume | 204.22 |
| LANDRACE hull volume | 121.77 |
| intersection of hulls volume | 111.24 |
| percentage area overlap | 0.78 |
| COMMERCIALpercentage area overlap | 0.70 |
| LANDRACEpercentage area overlap | 0.87 |
|  |  |
| **SEMIWILD-LANDRACE** |  |
| SEMIWILD hull area | 142.94 |
| LANDRACE hull area | 163.81 |
| intersection of hulls area | 127.08 |
| SEMIWILD hull volume | 104.92 |
| LANDRACE hull volume | 121.77 |
| intersection of hulls volume | 90.18 |
| percentage area overlap | 0.83 |
| SEMIWILDpercentage area overlap | 0.89 |
| LANDRACEpercentage area overlap | 0.78 |
|  |  |
| **SEMIWILD-COMMERCIAL** |  |
| SEMIWILD hull area | 142.94 |
| COMMERCIAL hull area | 202.49 |
| intersection of hulls area | 128.01 |
| SEMIWILD hull volume | 104.92 |
| COMMERCIAL hull volume | 204.22 |
| intersection of hulls volume | 96.87 |
| percentage area overlap | 0.74 |
| SEMIWILDpercentage area overlap | 0.90 |
| COMMERCIALpercentage area overlap | 0.63 |
