# Supplementary material for: Fluctuation of ecological niches and geographic range shifts along chile pepper's domestication gradient
Source: Ecol Evol. 2023 Nov 28;13(11):e10731. doi: 10.1002/ece3.10731 (PMC10682905; doi:10.1002/ece3.10731)
Supplement: Supplementary file 1 — Appendix S1 [file ECE3-13-e10731-s001.zip › SuppTable_S4.docx]

**Supplementary table 4**

| year | ssp | FirstInPair | SecondInPair | none | only_FirstInPair | only_SecondInPair | overlap | percent |
| --- | --- | --- | --- | --- | --- | --- | --- | --- |
| 2050 | 45 | ARVENSE | COMMERCIAL | 306379 | 39247 | 40977 | 30506 | 43.20 |
| 2050 | 85 | ARVENSE | COMMERCIAL | 308610 | 38505 | 40087 | 29907 | 43.22 |
| 2070 | 45 | ARVENSE | COMMERCIAL | 308709 | 38064 | 39251 | 31085 | 44.57 |
| 2070 | 85 | ARVENSE | COMMERCIAL | 310142 | 37665 | 39154 | 30148 | 43.97 |
| 2090 | 45 | ARVENSE | COMMERCIAL | 307880 | 39860 | 38978 | 30391 | 43.53 |
| 2090 | 85 | ARVENSE | COMMERCIAL | 310403 | 39611 | 37217 | 29878 | 43.75 |
| 2050 | 45 | ARVENSE | LANDRACE | 346047 | 29092 | 1309 | 40661 | 72.79 |
| 2050 | 85 | ARVENSE | LANDRACE | 347507 | 27864 | 1190 | 40548 | 73.62 |
| 2070 | 45 | ARVENSE | LANDRACE | 346895 | 30059 | 1065 | 39090 | 71.53 |
| 2070 | 85 | ARVENSE | LANDRACE | 348752 | 29352 | 544 | 38461 | 72.01 |
| 2090 | 45 | ARVENSE | LANDRACE | 346055 | 29052 | 803 | 41199 | 73.40 |
| 2090 | 85 | ARVENSE | LANDRACE | 347277 | 34059 | 343 | 35430 | 67.32 |
| 2050 | 45 | ARVENSE | WILD | 328060 | 18228 | 19296 | 51525 | 73.31 |
| 2050 | 85 | ARVENSE | WILD | 328557 | 18470 | 20140 | 49942 | 72.12 |
| 2070 | 45 | ARVENSE | WILD | 329096 | 18989 | 18864 | 50160 | 72.60 |
| 2070 | 85 | ARVENSE | WILD | 329101 | 19865 | 20195 | 47948 | 70.53 |
| 2090 | 45 | ARVENSE | WILD | 328873 | 18141 | 17985 | 52110 | 74.26 |
| 2090 | 85 | ARVENSE | WILD | 326961 | 25499 | 20659 | 43990 | 65.59 |
| 2050 | 45 | COMMERCIAL | LANDRACE | 325798 | 49341 | 19828 | 22142 | 39.03 |
| 2050 | 85 | COMMERCIAL | LANDRACE | 327055 | 48316 | 20060 | 21678 | 38.80 |
| 2070 | 45 | COMMERCIAL | LANDRACE | 329076 | 47878 | 17697 | 22458 | 40.65 |
| 2070 | 85 | COMMERCIAL | LANDRACE | 327948 | 50156 | 19859 | 19146 | 35.36 |
| 2090 | 45 | COMMERCIAL | LANDRACE | 327087 | 48020 | 20653 | 21349 | 38.34 |
| 2090 | 85 | COMMERCIAL | LANDRACE | 331312 | 50024 | 18702 | 17071 | 33.19 |
| 2050 | 45 | COMMERCIAL | WILD | 308427 | 37861 | 37199 | 33622 | 47.25 |
| 2050 | 85 | COMMERCIAL | WILD | 310128 | 36899 | 36987 | 33095 | 47.25 |
| 2070 | 45 | COMMERCIAL | WILD | 311088 | 36997 | 35685 | 33339 | 47.85 |
| 2070 | 85 | COMMERCIAL | WILD | 312342 | 36624 | 35465 | 32678 | 47.55 |
| 2090 | 45 | COMMERCIAL | WILD | 311494 | 35520 | 36246 | 33849 | 48.54 |
| 2090 | 85 | COMMERCIAL | WILD | 316540 | 35920 | 33474 | 31175 | 47.33 |
| 2050 | 45 | LANDRACE | WILD | 339952 | 6336 | 35187 | 35634 | 63.19 |
| 2050 | 85 | LANDRACE | WILD | 340211 | 6816 | 35160 | 34922 | 62.46 |
| 2070 | 45 | LANDRACE | WILD | 341298 | 6787 | 35656 | 33368 | 61.13 |
| 2070 | 85 | LANDRACE | WILD | 342964 | 6002 | 35140 | 33003 | 61.60 |
| 2090 | 45 | LANDRACE | WILD | 341582 | 5432 | 33525 | 36570 | 65.25 |
| 2090 | 85 | LANDRACE | WILD | 346364 | 6096 | 34972 | 29677 | 59.10 |
| 2050 | 45 | CULTIVATED | WILDsl | 306051 | 39792 | 31662 | 39604 | 52.57 |
| 2050 | 85 | CULTIVATED | WILDsl | 308168 | 38115 | 31864 | 38962 | 52.69 |
| 2070 | 45 | CULTIVATED | WILDsl | 308650 | 39121 | 31255 | 38083 | 51.98 |
| 2070 | 85 | CULTIVATED | WILDsl | 309870 | 38633 | 31541 | 37065 | 51.37 |
| 2090 | 45 | CULTIVATED | WILDsl | 308387 | 37492 | 30874 | 40356 | 54.14 |
| 2090 | 85 | CULTIVATED | WILDsl | 312699 | 38114 | 30618 | 35678 | 50.94 |
