# Supplementary material for: Fluctuation of ecological niches and geographic range shifts along chile pepper's domestication gradient
Source: Ecol Evol. 2023 Nov 28;13(11):e10731. doi: 10.1002/ece3.10731 (PMC10682905; doi:10.1002/ece3.10731)
Supplement: Supplementary file 1 — Appendix S1 [file ECE3-13-e10731-s001.zip › SuppTable_S5.docx]

**Supplementary table 5**

|  | #Training samples | Regularized training gain | Unregularized training gain | Iterations | Training AUC | #Test samples | Test gain | Test AUC | AUC Standard Deviation |
| --- | --- | --- | --- | --- | --- | --- | --- | --- | --- |
| Wild | 271.8 | 0.8495 | 1.0329 | 700 | 0.8714 | 30.2 | 0.9302 | 0.8559 | 0.0217 |
| Semiwild | 77.4 | 1.1297 | 1.3147 | 334 | 0.9001 | 8.6 | 1.1511 | 0.8841 | 0.0347 |
| Landrace | 249.3 | 1.3064 | 1.5128 | 690 | 0.9212 | 27.7 | 1.456 | 0.9157 | 0.0148 |
| Commercial | 1960.2 | 0.6455 | 0.7081 | 1194 | 0.8126 | 217.8 | 0.6923 | 0.809 | 0.0102 |
| Wild_sl | 330.3 | 0.8498 | 1.0036 | 656 | 0.8657 | 36.7 | 0.9387 | 0.8561 | 0.02 |
| Cultivated | 2224.8 | 0.573 | 0.6352 | 1046 | 0.7951 | 247.2 | 0.6211 | 0.7913 | 0.0101 |
|  | contribution | | | | | | | | |
|  | bio_14 | bio_15 | bio_18 | bio_19 | bio_2 | bio_3 | bio_4 | bio_5 | bio_9 |
| Wild | 1.1173 | 7.2201 | 41.7222 | 1.9789 | 4.3034 | 3.4145 | 29.0677 | 7.5937 | 3.5821 |
| Semiwild | 1.7299 | 0.0277 | 12.1187 | 5.352 | 2.4884 | 0.6326 | 70.1724 | 2.248 | 5.2302 |
| Landrace | 4.483 | 4.1452 | 5.4641 | 5.1419 | 5.5949 | 5.0484 | 69.5326 | 0.472 | 0.1177 |
| Commercial | 4.2893 | 45.0421 | 0.6327 | 8.7361 | 2.5235 | 19.5061 | 8.3892 | 1.2988 | 9.5822 |
| Wild_sl | 1.0763 | 2.4468 | 38.7077 | 3.3654 | 4.496 | 7.4522 | 31.2184 | 5.4351 | 5.8021 |
| Cultivated | 5.2589 | 26.1385 | 0.5607 | 7.6797 | 1.6585 | 39.1997 | 10.3586 | 1.9244 | 7.2211 |
|  | permutation | | | | | | | | |
|  | bio_14 | bio_15 | bio_18 | bio_19 | bio_2 | bio_3 | bio_4 | bio_5 | bio_9 |
| Wild | 6.1369 | 9.2432 | 27.5373 | 8.2303 | 6.5256 | 17.7181 | 7.1079 | 16.2013 | 1.2994 |
| Semiwild | 7.8949 | 0.0409 | 10.3086 | 9.9771 | 0.3308 | 0.1653 | 62.4933 | 8.789 | 0 |
| Landrace | 17.7008 | 5.2002 | 3.152 | 8.4909 | 1.3861 | 4.4064 | 58.3173 | 1.2829 | 0.0635 |
| Commercial | 10.0148 | 21.6384 | 1.4768 | 21.2309 | 2.2365 | 17.8384 | 13.1728 | 0.7322 | 11.6592 |
| Wild_sl | 6.5036 | 4.0372 | 36.9543 | 12.4955 | 3.615 | 18.455 | 5.9227 | 11.4114 | 0.6054 |
| Cultivated | 11.5519 | 16.64 | 1.0677 | 21.0796 | 1.0011 | 24.651 | 13.5664 | 1.0952 | 9.347 |
|  | Training gain without | | | | | | | | |
|  | bio_14 | bio_15 | bio_18 | bio_19 | bio_2 | bio_3 | bio_4 | bio_5 | bio_9 |
| Wild | 0.8426 | 0.8399 | 0.7996 | 0.8228 | 0.8301 | 0.8351 | 0.8381 | 0.8241 | 0.8467 |
| Semiwild | 1.0959 | 1.1297 | 1.0847 | 1.0768 | 1.1287 | 1.1293 | 1.0431 | 1.1124 | 1.1297 |
| Landrace | 1.1664 | 1.2684 | 1.2789 | 1.2332 | 1.2939 | 1.2761 | 1.2015 | 1.3047 | 1.3064 |
| Commercial | 0.6272 | 0.6061 | 0.6401 | 0.6083 | 0.6418 | 0.6362 | 0.6168 | 0.6427 | 0.6223 |
| Wild_sl | 0.8435 | 0.8448 | 0.7949 | 0.8152 | 0.8312 | 0.8353 | 0.8435 | 0.832 | 0.8485 |
| Cultivated | 0.552 | 0.5472 | 0.5684 | 0.5423 | 0.5718 | 0.5613 | 0.5443 | 0.5706 | 0.5534 |
|  | Training gain with | | | | | | | | |
|  | bio_14 | bio_15 | bio_18 | bio_19 | bio_2 | bio_3 | bio_4 | bio_5 | bio_9 |
| Wild | 0.007 | 0.2389 | 0.5065 | 0.0653 | 0.1907 | 0.4146 | 0.4843 | 0.0326 | 0.2929 |
| Semiwild | 0.0219 | 0.1411 | 0.5684 | 0.1168 | 0.2385 | 0.7234 | 0.8658 | 0.0089 | 0.4336 |
| Landrace | 0.0962 | 0.1838 | 0.5472 | 0.1711 | 0.3992 | 0.602 | 0.9184 | 0.0324 | 0.2854 |
| Commercial | 0.1549 | 0.37 | 0.0511 | 0.1566 | 0.0629 | 0.3515 | 0.2964 | 0.0302 | 0.1678 |
| Wild_sl | 0.0104 | 0.2196 | 0.5091 | 0.0722 | 0.2095 | 0.441 | 0.51 | 0.0273 | 0.3109 |
| Cultivated | 0.0972 | 0.3026 | 0.0565 | 0.1076 | 0.0378 | 0.3364 | 0.2899 | 0.028 | 0.1519 |
|  | Test gain without | | | | | | | | |
|  | bio_14 | bio_15 | bio_18 | bio_19 | bio_2 | bio_3 | bio_4 | bio_5 | bio_9 |
| Wild | 0.9271 | 0.9201 | 0.9212 | 0.8845 | 0.906 | 0.9012 | 0.926 | 0.9169 | 0.9251 |
| Semiwild | 1.1042 | 1.1528 | 1.1106 | 1.0929 | 1.1605 | 1.1532 | 1.1014 | 1.1393 | 1.1512 |
| Landrace | 1.3628 | 1.4462 | 1.4116 | 1.4005 | 1.4344 | 1.4179 | 1.3369 | 1.4584 | 1.4568 |
| Commercial | 0.6696 | 0.6552 | 0.6822 | 0.6551 | 0.6883 | 0.6846 | 0.6611 | 0.6884 | 0.6688 |
| Wild_sl | 0.936 | 0.9296 | 0.9183 | 0.897 | 0.9178 | 0.9286 | 0.9371 | 0.9346 | 0.9345 |
| Cultivated | 0.5976 | 0.5955 | 0.6121 | 0.5897 | 0.6201 | 0.6095 | 0.5868 | 0.6177 | 0.6 |
|  | Test gain with only | | | | | | | | |
|  | bio_14 | bio_15 | bio_18 | bio_19 | bio_2 | bio_3 | bio_4 | bio_5 | bio_9 |
| Wild | 0.0058 | 0.2517 | 0.5546 | 0.1143 | 0.2242 | 0.4463 | 0.5167 | 0.0456 | 0.3255 |
| Semiwild | -0.0021 | 0.175 | 0.6446 | 0.2077 | 0.2934 | 0.769 | 0.8974 | 0.0174 | 0.4417 |
| Landrace | 0.1308 | 0.2101 | 0.5939 | 0.2904 | 0.4418 | 0.636 | 0.9814 | 0.0499 | 0.3042 |
| Commercial | 0.1763 | 0.377 | 0.0652 | 0.1625 | 0.0663 | 0.3693 | 0.3105 | 0.0397 | 0.1778 |
| Wild_sl | 0.0171 | 0.2383 | 0.5536 | 0.1186 | 0.2447 | 0.4676 | 0.5425 | 0.0401 | 0.3439 |
| Cultivated | 0.1152 | 0.3104 | 0.0687 | 0.1129 | 0.0413 | 0.352 | 0.3032 | 0.0346 | 0.1619 |
|  | AUC without | | | | | | | | |
|  | bio_14 | bio_15 | bio_18 | bio_19 | bio_2 | bio_3 | bio_4 | bio_5 | bio_9 |
| Wild | 0.855 | 0.8543 | 0.8546 | 0.8477 | 0.8522 | 0.8512 | 0.8551 | 0.8534 | 0.8557 |
| Semiwild | 0.8793 | 0.8843 | 0.8796 | 0.878 | 0.8848 | 0.8845 | 0.8773 | 0.8839 | 0.8841 |
| Landrace | 0.9119 | 0.9163 | 0.9123 | 0.912 | 0.9141 | 0.9127 | 0.905 | 0.9159 | 0.9158 |
| Commercial | 0.8049 | 0.8011 | 0.8072 | 0.8019 | 0.8079 | 0.8078 | 0.8024 | 0.8079 | 0.8035 |
| Wild_sl | 0.8557 | 0.8542 | 0.8537 | 0.8483 | 0.8534 | 0.8549 | 0.8556 | 0.8554 | 0.8557 |
| Cultivated | 0.7861 | 0.7853 | 0.7897 | 0.7849 | 0.791 | 0.7897 | 0.7837 | 0.7903 | 0.7861 |
|  | AUC with only | | | | | | | | |
|  | bio_14 | bio_15 | bio_18 | bio_19 | bio_2 | bio_3 | bio_4 | bio_5 | bio_9 |
| Wild | 0.5422 | 0.6856 | 0.7696 | 0.5978 | 0.6897 | 0.7277 | 0.7649 | 0.5771 | 0.7141 |
| Semiwild | 0.5206 | 0.6559 | 0.8127 | 0.632 | 0.723 | 0.8247 | 0.8158 | 0.5945 | 0.7619 |
| Landrace | 0.6457 | 0.6582 | 0.782 | 0.713 | 0.7608 | 0.7811 | 0.8534 | 0.5331 | 0.712 |
| Commercial | 0.665 | 0.7237 | 0.5916 | 0.6569 | 0.5923 | 0.7066 | 0.7033 | 0.5772 | 0.6342 |
| Wild_sl | 0.5475 | 0.6738 | 0.777 | 0.5965 | 0.6995 | 0.7389 | 0.7738 | 0.5664 | 0.7131 |
| Cultivated | 0.6346 | 0.6996 | 0.5952 | 0.6317 | 0.5701 | 0.6958 | 0.7022 | 0.5708 | 0.6226 |
