# Supplementary material for: Fluctuation of ecological niches and geographic range shifts along chile pepper's domestication gradient
Source: Ecol Evol. 2023 Nov 28;13(11):e10731. doi: 10.1002/ece3.10731 (PMC10682905; doi:10.1002/ece3.10731)
Supplement: Supplementary file 1 — Appendix S1 [file ECE3-13-e10731-s001.zip › SuppTable_S6.docx]

**Supplementary table 6**

| comparisonData1 | comparisonData2 | D | I | rank.cor |
| --- | --- | --- | --- | --- |
| SEMIWILD | COMMERCIAL | 0.526 | 0.797 | 0.755 |
| SEMIWILD | LANDRACE | 0.793 | 0.951 | 0.874 |
| SEMIWILD | WILD | 0.718 | 0.926 | 0.928 |
| SEMIWILD | CULTIVATED | 0.570 | 0.829 | 0.785 |
| SEMIWILD | WILDsl | 0.752 | 0.939 | 0.939 |
| COMMERCIAL | LANDRACE | 0.487 | 0.746 | 0.491 |
| COMMERCIAL | WILD | 0.611 | 0.855 | 0.760 |
| COMMERCIAL | CULTIVATED | 0.933 | 0.995 | 0.995 |
| COMMERCIAL | WILDsl | 0.603 | 0.851 | 0.759 |
| LANDRACE | WILD | 0.683 | 0.892 | 0.800 |
| LANDRACE | CULTIVATED | 0.526 | 0.775 | 0.526 |
| LANDRACE | WILDsl | 0.714 | 0.908 | 0.829 |
| WILD | CULTIVATED | 0.660 | 0.885 | 0.792 |
| WILD | WILDsl | 0.946 | 0.997 | 0.990 |
