# Supplementary material for: Fluctuation of ecological niches and geographic range shifts along chile pepper's domestication gradient
Source: Ecol Evol. 2023 Nov 28;13(11):e10731. doi: 10.1002/ece3.10731 (PMC10682905; doi:10.1002/ece3.10731)
Supplement: Supplementary file 1 — Appendix S1 [file ECE3-13-e10731-s001.zip › SuppTable_S7.docx]

Supplementary table S7

| **Single GCM models** | | |  |  |  |  |  |  |
| --- | --- | --- | --- | --- | --- | --- | --- | --- |
| year | ssp | GCM | Dom | none | pixLost | pixNew | pixKeep | percent |
| 2050 | 45 | BCC-CSM2-MR | SEMIWILD | 330066 | 891 | 14900 | 71252 | 90.02 |
| 2050 | 45 | CNRM-CM6-1 | SEMIWILD | 328664 | 1015 | 16302 | 71128 | 89.15 |
| 2050 | 45 | CNRM-ESM2-1 | SEMIWILD | 331587 | 2257 | 13379 | 69886 | 89.94 |
| 2050 | 45 | CanESM5 | SEMIWILD | 319371 | 1634 | 25595 | 70509 | 83.82 |
| 2050 | 45 | IPSL-CM6A-LR | SEMIWILD | 328026 | 2110 | 16940 | 70033 | 88.03 |
| 2050 | 45 | MIROC-ES2L | SEMIWILD | 332945 | 4055 | 12021 | 68088 | 89.44 |
| 2050 | 45 | MIROC6 | SEMIWILD | 328663 | 4926 | 16303 | 67217 | 86.36 |
| 2050 | 45 | MRI-ESM2-0 | SEMIWILD | 327540 | 3498 | 17426 | 68645 | 86.77 |
| 2050 | 85 | BCC-CSM2-MR | SEMIWILD | 324766 | 185 | 20200 | 71958 | 87.59 |
| 2050 | 85 | CNRM-CM6-1 | SEMIWILD | 329759 | 1341 | 15207 | 70802 | 89.54 |
| 2050 | 85 | CNRM-ESM2-1 | SEMIWILD | 329014 | 1658 | 15952 | 70485 | 88.9 |
| 2050 | 85 | CanESM5 | SEMIWILD | 312962 | 1706 | 32004 | 70437 | 80.69 |
| 2050 | 85 | IPSL-CM6A-LR | SEMIWILD | 324962 | 1572 | 20004 | 70571 | 86.74 |
| 2050 | 85 | MIROC-ES2L | SEMIWILD | 331775 | 7772 | 13191 | 64371 | 86 |
| 2050 | 85 | MIROC6 | SEMIWILD | 325530 | 4778 | 19436 | 67365 | 84.77 |
| 2050 | 85 | MRI-ESM2-0 | SEMIWILD | 329832 | 4622 | 15134 | 67521 | 87.24 |
| 2070 | 45 | BCC-CSM2-MR | SEMIWILD | 324791 | 252 | 20175 | 71891 | 87.56 |
| 2070 | 45 | CNRM-CM6-1 | SEMIWILD | 327726 | 1400 | 17240 | 70743 | 88.36 |
| 2070 | 45 | CNRM-ESM2-1 | SEMIWILD | 328896 | 2274 | 16070 | 69869 | 88.4 |
| 2070 | 45 | CanESM5 | SEMIWILD | 314680 | 1652 | 30286 | 70491 | 81.53 |
| 2070 | 45 | IPSL-CM6A-LR | SEMIWILD | 326584 | 1751 | 18382 | 70392 | 87.49 |
| 2070 | 45 | MIROC-ES2L | SEMIWILD | 329507 | 6881 | 15459 | 65262 | 85.39 |
| 2070 | 45 | MIROC6 | SEMIWILD | 325101 | 5312 | 19865 | 66831 | 84.15 |
| 2070 | 45 | MRI-ESM2-0 | SEMIWILD | 326559 | 2555 | 18407 | 69588 | 86.91 |
| 2070 | 85 | BCC-CSM2-MR | SEMIWILD | 316336 | 283 | 28630 | 71860 | 83.25 |
| 2070 | 85 | CNRM-CM6-1 | SEMIWILD | 324151 | 1089 | 20815 | 71054 | 86.64 |
| 2070 | 85 | CNRM-ESM2-1 | SEMIWILD | 325394 | 2328 | 19572 | 69815 | 86.44 |
| 2070 | 85 | CanESM5 | SEMIWILD | 301767 | 1435 | 43199 | 70708 | 76.01 |
| 2070 | 85 | IPSL-CM6A-LR | SEMIWILD | 316719 | 1547 | 28247 | 70596 | 82.58 |
| 2070 | 85 | MIROC-ES2L | SEMIWILD | 325127 | 10243 | 19839 | 61900 | 80.45 |
| 2070 | 85 | MIROC6 | SEMIWILD | 313037 | 3863 | 31929 | 68280 | 79.23 |
| 2070 | 85 | MRI-ESM2-0 | SEMIWILD | 320785 | 4205 | 24181 | 67938 | 82.72 |
| 2090 | 45 | BCC-CSM2-MR | SEMIWILD | 326445 | 704 | 18521 | 71439 | 88.14 |
| 2090 | 45 | CNRM-CM6-1 | SEMIWILD | 326663 | 1100 | 18303 | 71043 | 87.98 |
| 2090 | 45 | CNRM-ESM2-1 | SEMIWILD | 327396 | 1872 | 17570 | 70271 | 87.85 |
| 2090 | 45 | CanESM5 | SEMIWILD | 313060 | 1732 | 31906 | 70411 | 80.72 |
| 2090 | 45 | IPSL-CM6A-LR | SEMIWILD | 320864 | 1456 | 24102 | 70687 | 84.69 |
| 2090 | 45 | MIROC-ES2L | SEMIWILD | 331569 | 6083 | 13397 | 66060 | 87.15 |
| 2090 | 45 | MIROC6 | SEMIWILD | 324226 | 4219 | 20740 | 67924 | 84.48 |
| 2090 | 45 | MRI-ESM2-0 | SEMIWILD | 325957 | 3306 | 19009 | 68837 | 86.05 |
| 2090 | 85 | BCC-CSM2-MR | SEMIWILD | 312458 | 109 | 32508 | 72034 | 81.54 |
| 2090 | 85 | CNRM-CM6-1 | SEMIWILD | 320024 | 1975 | 24942 | 70168 | 83.91 |
| 2090 | 85 | CNRM-ESM2-1 | SEMIWILD | 322222 | 2431 | 22744 | 69712 | 84.71 |
| 2090 | 85 | CanESM5 | SEMIWILD | 293230 | 1318 | 51736 | 70825 | 72.75 |
| 2090 | 85 | IPSL-CM6A-LR | SEMIWILD | 312588 | 3299 | 32378 | 68844 | 79.42 |
| 2090 | 85 | MIROC-ES2L | SEMIWILD | 314962 | 8672 | 30004 | 63471 | 76.65 |
| 2090 | 85 | MIROC6 | SEMIWILD | 303708 | 4462 | 41258 | 67681 | 74.75 |
| 2090 | 85 | MRI-ESM2-0 | SEMIWILD | 316752 | 4999 | 28214 | 67144 | 80.17 |
| 2050 | 45 | BCC-CSM2-MR | COMMERCIAL | 330038 | 846 | 21703 | 64522 | 85.13 |
| 2050 | 45 | CNRM-CM6-1 | COMMERCIAL | 332763 | 1197 | 18978 | 64171 | 86.42 |
| 2050 | 45 | CNRM-ESM2-1 | COMMERCIAL | 327051 | 856 | 24690 | 64512 | 83.47 |
| 2050 | 45 | CanESM5 | COMMERCIAL | 326128 | 893 | 25613 | 64475 | 82.95 |
| 2050 | 45 | IPSL-CM6A-LR | COMMERCIAL | 331851 | 908 | 19890 | 64460 | 86.11 |
| 2050 | 45 | MIROC-ES2L | COMMERCIAL | 325691 | 401 | 26050 | 64967 | 83.09 |
| 2050 | 45 | MIROC6 | COMMERCIAL | 322699 | 335 | 29042 | 65033 | 81.58 |
| 2050 | 45 | MRI-ESM2-0 | COMMERCIAL | 331661 | 1052 | 20080 | 64316 | 85.89 |
| 2050 | 85 | BCC-CSM2-MR | COMMERCIAL | 335348 | 2273 | 16393 | 63095 | 87.11 |
| 2050 | 85 | CNRM-CM6-1 | COMMERCIAL | 328358 | 1253 | 23383 | 64115 | 83.88 |
| 2050 | 85 | CNRM-ESM2-1 | COMMERCIAL | 327704 | 1324 | 24037 | 64044 | 83.47 |
| 2050 | 85 | CanESM5 | COMMERCIAL | 325412 | 1605 | 26329 | 63763 | 82.03 |
| 2050 | 85 | IPSL-CM6A-LR | COMMERCIAL | 330111 | 1883 | 21630 | 63485 | 84.37 |
| 2050 | 85 | MIROC-ES2L | COMMERCIAL | 323557 | 1493 | 28184 | 63875 | 81.15 |
| 2050 | 85 | MIROC6 | COMMERCIAL | 319380 | 355 | 32361 | 65013 | 79.9 |
| 2050 | 85 | MRI-ESM2-0 | COMMERCIAL | 333618 | 2446 | 18123 | 62922 | 85.95 |
| 2070 | 45 | BCC-CSM2-MR | COMMERCIAL | 328909 | 2283 | 22832 | 63085 | 83.4 |
| 2070 | 45 | CNRM-CM6-1 | COMMERCIAL | 327593 | 1373 | 24148 | 63995 | 83.38 |
| 2070 | 45 | CNRM-ESM2-1 | COMMERCIAL | 326410 | 1533 | 25331 | 63835 | 82.62 |
| 2070 | 45 | CanESM5 | COMMERCIAL | 323659 | 1272 | 28082 | 64096 | 81.37 |
| 2070 | 45 | IPSL-CM6A-LR | COMMERCIAL | 332070 | 2280 | 19671 | 63088 | 85.18 |
| 2070 | 45 | MIROC-ES2L | COMMERCIAL | 326783 | 1695 | 24958 | 63673 | 82.69 |
| 2070 | 45 | MIROC6 | COMMERCIAL | 314803 | 1011 | 36938 | 64357 | 77.23 |
| 2070 | 45 | MRI-ESM2-0 | COMMERCIAL | 335127 | 2118 | 16614 | 63250 | 87.1 |
| 2070 | 85 | BCC-CSM2-MR | COMMERCIAL | 330681 | 4173 | 21060 | 61195 | 82.91 |
| 2070 | 85 | CNRM-CM6-1 | COMMERCIAL | 325442 | 2514 | 26299 | 62854 | 81.35 |
| 2070 | 85 | CNRM-ESM2-1 | COMMERCIAL | 325890 | 2632 | 25851 | 62736 | 81.5 |
| 2070 | 85 | CanESM5 | COMMERCIAL | 324011 | 3103 | 27730 | 62265 | 80.15 |
| 2070 | 85 | IPSL-CM6A-LR | COMMERCIAL | 326549 | 4325 | 25192 | 61043 | 80.53 |
| 2070 | 85 | MIROC-ES2L | COMMERCIAL | 317631 | 2135 | 34110 | 63233 | 77.72 |
| 2070 | 85 | MIROC6 | COMMERCIAL | 310303 | 2105 | 41438 | 63263 | 74.4 |
| 2070 | 85 | MRI-ESM2-0 | COMMERCIAL | 321306 | 1223 | 30435 | 64145 | 80.21 |
| 2090 | 45 | BCC-CSM2-MR | COMMERCIAL | 330878 | 1438 | 20863 | 63930 | 85.15 |
| 2090 | 45 | CNRM-CM6-1 | COMMERCIAL | 330607 | 2126 | 21134 | 63242 | 84.47 |
| 2090 | 45 | CNRM-ESM2-1 | COMMERCIAL | 323753 | 1552 | 27988 | 63816 | 81.21 |
| 2090 | 45 | CanESM5 | COMMERCIAL | 324338 | 1762 | 27403 | 63606 | 81.35 |
| 2090 | 45 | IPSL-CM6A-LR | COMMERCIAL | 327643 | 2260 | 24098 | 63108 | 82.72 |
| 2090 | 45 | MIROC-ES2L | COMMERCIAL | 323607 | 1626 | 28134 | 63742 | 81.07 |
| 2090 | 45 | MIROC6 | COMMERCIAL | 317269 | 710 | 34472 | 64658 | 78.61 |
| 2090 | 45 | MRI-ESM2-0 | COMMERCIAL | 331428 | 3156 | 20313 | 62212 | 84.13 |
| 2090 | 85 | BCC-CSM2-MR | COMMERCIAL | 328492 | 4594 | 23249 | 60774 | 81.36 |
| 2090 | 85 | CNRM-CM6-1 | COMMERCIAL | 325028 | 4240 | 26713 | 61128 | 79.8 |
| 2090 | 85 | CNRM-ESM2-1 | COMMERCIAL | 325075 | 4641 | 26666 | 60727 | 79.51 |
| 2090 | 85 | CanESM5 | COMMERCIAL | 324774 | 5173 | 26967 | 60195 | 78.93 |
| 2090 | 85 | IPSL-CM6A-LR | COMMERCIAL | 327197 | 6354 | 24544 | 59014 | 79.25 |
| 2090 | 85 | MIROC-ES2L | COMMERCIAL | 313060 | 2850 | 38681 | 62518 | 75.07 |
| 2090 | 85 | MIROC6 | COMMERCIAL | 304844 | 2387 | 46897 | 62981 | 71.88 |
| 2090 | 85 | MRI-ESM2-0 | COMMERCIAL | 316816 | 2038 | 34925 | 63330 | 77.41 |
| 2050 | 45 | BCC-CSM2-MR | LANDRACE | 357356 | 1224 | 12657 | 45872 | 86.86 |
| 2050 | 45 | CNRM-CM6-1 | LANDRACE | 355770 | 958 | 14243 | 46138 | 85.86 |
| 2050 | 45 | CNRM-ESM2-1 | LANDRACE | 357206 | 1198 | 12807 | 45898 | 86.76 |
| 2050 | 45 | CanESM5 | LANDRACE | 349176 | 494 | 20837 | 46602 | 81.38 |
| 2050 | 45 | IPSL-CM6A-LR | LANDRACE | 356487 | 1623 | 13526 | 45473 | 85.72 |
| 2050 | 45 | MIROC-ES2L | LANDRACE | 359888 | 4549 | 10125 | 42547 | 85.29 |
| 2050 | 45 | MIROC6 | LANDRACE | 357272 | 6070 | 12741 | 41026 | 81.35 |
| 2050 | 45 | MRI-ESM2-0 | LANDRACE | 354474 | 3720 | 15539 | 43376 | 81.83 |
| 2050 | 85 | BCC-CSM2-MR | LANDRACE | 355245 | 1031 | 14768 | 46065 | 85.36 |
| 2050 | 85 | CNRM-CM6-1 | LANDRACE | 356521 | 1191 | 13492 | 45905 | 86.21 |
| 2050 | 85 | CNRM-ESM2-1 | LANDRACE | 354651 | 823 | 15362 | 46273 | 85.11 |
| 2050 | 85 | CanESM5 | LANDRACE | 346363 | 670 | 23650 | 46426 | 79.24 |
| 2050 | 85 | IPSL-CM6A-LR | LANDRACE | 353592 | 2649 | 16421 | 44447 | 82.34 |
| 2050 | 85 | MIROC-ES2L | LANDRACE | 359256 | 6960 | 10757 | 40136 | 81.92 |
| 2050 | 85 | MIROC6 | LANDRACE | 356574 | 5873 | 13439 | 41223 | 81.02 |
| 2050 | 85 | MRI-ESM2-0 | LANDRACE | 357496 | 4136 | 12517 | 42960 | 83.76 |
| 2070 | 45 | BCC-CSM2-MR | LANDRACE | 353870 | 790 | 16143 | 46306 | 84.54 |
| 2070 | 45 | CNRM-CM6-1 | LANDRACE | 355445 | 1619 | 14568 | 45477 | 84.89 |
| 2070 | 45 | CNRM-ESM2-1 | LANDRACE | 355930 | 1576 | 14083 | 45520 | 85.32 |
| 2070 | 45 | CanESM5 | LANDRACE | 347259 | 633 | 22754 | 46463 | 79.89 |
| 2070 | 45 | IPSL-CM6A-LR | LANDRACE | 356271 | 2801 | 13742 | 44295 | 84.26 |
| 2070 | 45 | MIROC-ES2L | LANDRACE | 356780 | 7877 | 13233 | 39219 | 78.79 |
| 2070 | 45 | MIROC6 | LANDRACE | 357200 | 7488 | 12813 | 39608 | 79.6 |
| 2070 | 45 | MRI-ESM2-0 | LANDRACE | 353040 | 1706 | 16973 | 45390 | 82.94 |
| 2070 | 85 | BCC-CSM2-MR | LANDRACE | 350896 | 1397 | 19117 | 45699 | 81.67 |
| 2070 | 85 | CNRM-CM6-1 | LANDRACE | 352625 | 1495 | 17388 | 45601 | 82.85 |
| 2070 | 85 | CNRM-ESM2-1 | LANDRACE | 352174 | 1591 | 17839 | 45505 | 82.41 |
| 2070 | 85 | CanESM5 | LANDRACE | 343933 | 2216 | 26080 | 44880 | 76.03 |
| 2070 | 85 | IPSL-CM6A-LR | LANDRACE | 350574 | 2813 | 19439 | 44283 | 79.92 |
| 2070 | 85 | MIROC-ES2L | LANDRACE | 355638 | 8386 | 14375 | 38710 | 77.28 |
| 2070 | 85 | MIROC6 | LANDRACE | 350305 | 7806 | 19708 | 39290 | 74.07 |
| 2070 | 85 | MRI-ESM2-0 | LANDRACE | 351562 | 2530 | 18451 | 44566 | 80.95 |
| 2090 | 45 | BCC-CSM2-MR | LANDRACE | 355766 | 1739 | 14247 | 45357 | 85.02 |
| 2090 | 45 | CNRM-CM6-1 | LANDRACE | 356289 | 1462 | 13724 | 45634 | 85.73 |
| 2090 | 45 | CNRM-ESM2-1 | LANDRACE | 354244 | 1277 | 15769 | 45819 | 84.32 |
| 2090 | 45 | CanESM5 | LANDRACE | 346828 | 770 | 23185 | 46326 | 79.46 |
| 2090 | 45 | IPSL-CM6A-LR | LANDRACE | 354536 | 2583 | 15477 | 44513 | 83.14 |
| 2090 | 45 | MIROC-ES2L | LANDRACE | 357832 | 6132 | 12181 | 40964 | 81.73 |
| 2090 | 45 | MIROC6 | LANDRACE | 355669 | 5993 | 14344 | 41103 | 80.17 |
| 2090 | 45 | MRI-ESM2-0 | LANDRACE | 351232 | 1985 | 18781 | 45111 | 81.29 |
| 2090 | 85 | BCC-CSM2-MR | LANDRACE | 347878 | 2044 | 22135 | 45052 | 78.84 |
| 2090 | 85 | CNRM-CM6-1 | LANDRACE | 350693 | 2646 | 19320 | 44450 | 80.19 |
| 2090 | 85 | CNRM-ESM2-1 | LANDRACE | 350958 | 2270 | 19055 | 44826 | 80.78 |
| 2090 | 85 | CanESM5 | LANDRACE | 340647 | 4717 | 29366 | 42379 | 71.32 |
| 2090 | 85 | IPSL-CM6A-LR | LANDRACE | 349567 | 5223 | 20446 | 41873 | 76.54 |
| 2090 | 85 | MIROC-ES2L | LANDRACE | 352606 | 7782 | 17407 | 39314 | 75.74 |
| 2090 | 85 | MIROC6 | LANDRACE | 350538 | 8815 | 19475 | 38281 | 73.02 |
| 2090 | 85 | MRI-ESM2-0 | LANDRACE | 351899 | 4491 | 18114 | 42605 | 79.03 |
| 2050 | 45 | BCC-CSM2-MR | WILD | 327003 | 633 | 16875 | 72598 | 89.24 |
| 2050 | 45 | CNRM-CM6-1 | WILD | 326857 | 1623 | 17021 | 71608 | 88.48 |
| 2050 | 45 | CNRM-ESM2-1 | WILD | 325835 | 2350 | 18043 | 70881 | 87.42 |
| 2050 | 45 | CanESM5 | WILD | 309023 | 1683 | 34855 | 71548 | 79.66 |
| 2050 | 45 | IPSL-CM6A-LR | WILD | 325172 | 3768 | 18706 | 69463 | 86.08 |
| 2050 | 45 | MIROC-ES2L | WILD | 326200 | 3638 | 17678 | 69593 | 86.72 |
| 2050 | 45 | MIROC6 | WILD | 323345 | 3394 | 20533 | 69837 | 85.37 |
| 2050 | 45 | MRI-ESM2-0 | WILD | 318045 | 4448 | 25833 | 68783 | 81.96 |
| 2050 | 85 | BCC-CSM2-MR | WILD | 324188 | 302 | 19690 | 72929 | 87.95 |
| 2050 | 85 | CNRM-CM6-1 | WILD | 325675 | 1821 | 18203 | 71410 | 87.7 |
| 2050 | 85 | CNRM-ESM2-1 | WILD | 325143 | 2053 | 18735 | 71178 | 87.26 |
| 2050 | 85 | CanESM5 | WILD | 302572 | 2186 | 41306 | 71045 | 76.56 |
| 2050 | 85 | IPSL-CM6A-LR | WILD | 321680 | 3629 | 22198 | 69602 | 84.35 |
| 2050 | 85 | MIROC-ES2L | WILD | 322789 | 4175 | 21089 | 69056 | 84.54 |
| 2050 | 85 | MIROC6 | WILD | 321140 | 4868 | 22738 | 68363 | 83.2 |
| 2050 | 85 | MRI-ESM2-0 | WILD | 322085 | 3081 | 21793 | 70150 | 84.94 |
| 2070 | 45 | BCC-CSM2-MR | WILD | 324618 | 1114 | 19260 | 72117 | 87.62 |
| 2070 | 45 | CNRM-CM6-1 | WILD | 325937 | 2914 | 17941 | 70317 | 87.09 |
| 2070 | 45 | CNRM-ESM2-1 | WILD | 321212 | 2687 | 22666 | 70544 | 84.77 |
| 2070 | 45 | CanESM5 | WILD | 304033 | 2149 | 39845 | 71082 | 77.2 |
| 2070 | 45 | IPSL-CM6A-LR | WILD | 322421 | 3164 | 21457 | 70067 | 85.06 |
| 2070 | 45 | MIROC-ES2L | WILD | 322553 | 4448 | 21325 | 68783 | 84.22 |
| 2070 | 45 | MIROC6 | WILD | 318709 | 6074 | 25169 | 67157 | 81.13 |
| 2070 | 45 | MRI-ESM2-0 | WILD | 316465 | 2233 | 27413 | 70998 | 82.73 |
| 2070 | 85 | BCC-CSM2-MR | WILD | 313559 | 885 | 30319 | 72346 | 82.26 |
| 2070 | 85 | CNRM-CM6-1 | WILD | 317491 | 2504 | 26387 | 70727 | 83.04 |
| 2070 | 85 | CNRM-ESM2-1 | WILD | 316264 | 2489 | 27614 | 70742 | 82.46 |
| 2070 | 85 | CanESM5 | WILD | 292043 | 4147 | 51835 | 69084 | 71.17 |
| 2070 | 85 | IPSL-CM6A-LR | WILD | 310358 | 4080 | 33520 | 69151 | 78.62 |
| 2070 | 85 | MIROC-ES2L | WILD | 308128 | 6777 | 35750 | 66454 | 75.76 |
| 2070 | 85 | MIROC6 | WILD | 307117 | 5655 | 36761 | 67576 | 76.11 |
| 2070 | 85 | MRI-ESM2-0 | WILD | 308478 | 4230 | 35400 | 69001 | 77.69 |
| 2090 | 45 | BCC-CSM2-MR | WILD | 324074 | 1202 | 19804 | 72029 | 87.27 |
| 2090 | 45 | CNRM-CM6-1 | WILD | 323482 | 3003 | 20396 | 70228 | 85.72 |
| 2090 | 45 | CNRM-ESM2-1 | WILD | 318610 | 2428 | 25268 | 70803 | 83.64 |
| 2090 | 45 | CanESM5 | WILD | 301930 | 1938 | 41948 | 71293 | 76.47 |
| 2090 | 45 | IPSL-CM6A-LR | WILD | 316442 | 3217 | 27436 | 70014 | 82.04 |
| 2090 | 45 | MIROC-ES2L | WILD | 322002 | 5511 | 21876 | 67720 | 83.18 |
| 2090 | 45 | MIROC6 | WILD | 318631 | 3451 | 25247 | 69780 | 82.94 |
| 2090 | 45 | MRI-ESM2-0 | WILD | 318472 | 3333 | 25406 | 69898 | 82.95 |
| 2090 | 85 | BCC-CSM2-MR | WILD | 310235 | 1549 | 33643 | 71682 | 80.29 |
| 2090 | 85 | CNRM-CM6-1 | WILD | 307906 | 4013 | 35972 | 69218 | 77.59 |
| 2090 | 85 | CNRM-ESM2-1 | WILD | 308681 | 3191 | 35197 | 70040 | 78.49 |
| 2090 | 85 | CanESM5 | WILD | 284775 | 7764 | 59103 | 65467 | 66.19 |
| 2090 | 85 | IPSL-CM6A-LR | WILD | 302631 | 5462 | 41247 | 67769 | 74.37 |
| 2090 | 85 | MIROC-ES2L | WILD | 293934 | 4281 | 49944 | 68950 | 71.78 |
| 2090 | 85 | MIROC6 | WILD | 287872 | 6935 | 56006 | 66296 | 67.81 |
| 2090 | 85 | MRI-ESM2-0 | WILD | 299067 | 5336 | 44811 | 67895 | 73.03 |
| 2050 | 45 | BCC-CSM2-MR | CULTIVATED | 323250 | 762 | 22449 | 70648 | 85.89 |
| 2050 | 45 | CNRM-CM6-1 | CULTIVATED | 323360 | 751 | 22339 | 70659 | 85.96 |
| 2050 | 45 | CNRM-ESM2-1 | CULTIVATED | 318460 | 520 | 27239 | 70890 | 83.63 |
| 2050 | 45 | CanESM5 | CULTIVATED | 317207 | 431 | 28492 | 70979 | 83.07 |
| 2050 | 45 | IPSL-CM6A-LR | CULTIVATED | 323177 | 743 | 22522 | 70667 | 85.87 |
| 2050 | 45 | MIROC-ES2L | CULTIVATED | 316677 | 289 | 29022 | 71121 | 82.91 |
| 2050 | 45 | MIROC6 | CULTIVATED | 315317 | 378 | 30382 | 71032 | 82.2 |
| 2050 | 45 | MRI-ESM2-0 | CULTIVATED | 323030 | 874 | 22669 | 70536 | 85.7 |
| 2050 | 85 | BCC-CSM2-MR | CULTIVATED | 326476 | 2155 | 19223 | 69255 | 86.63 |
| 2050 | 85 | CNRM-CM6-1 | CULTIVATED | 319593 | 807 | 26106 | 70603 | 83.99 |
| 2050 | 85 | CNRM-ESM2-1 | CULTIVATED | 317832 | 816 | 27867 | 70594 | 83.11 |
| 2050 | 85 | CanESM5 | CULTIVATED | 315680 | 690 | 30019 | 70720 | 82.16 |
| 2050 | 85 | IPSL-CM6A-LR | CULTIVATED | 320909 | 1127 | 24790 | 70283 | 84.43 |
| 2050 | 85 | MIROC-ES2L | CULTIVATED | 314693 | 809 | 31006 | 70601 | 81.61 |
| 2050 | 85 | MIROC6 | CULTIVATED | 312145 | 416 | 33554 | 70994 | 80.69 |
| 2050 | 85 | MRI-ESM2-0 | CULTIVATED | 324746 | 2214 | 20953 | 69196 | 85.66 |
| 2070 | 45 | BCC-CSM2-MR | CULTIVATED | 321881 | 3178 | 23818 | 68232 | 83.48 |
| 2070 | 45 | CNRM-CM6-1 | CULTIVATED | 318412 | 1014 | 27287 | 70396 | 83.26 |
| 2070 | 45 | CNRM-ESM2-1 | CULTIVATED | 316753 | 957 | 28946 | 70453 | 82.49 |
| 2070 | 45 | CanESM5 | CULTIVATED | 314301 | 422 | 31398 | 70988 | 81.69 |
| 2070 | 45 | IPSL-CM6A-LR | CULTIVATED | 323747 | 2203 | 21952 | 69207 | 85.14 |
| 2070 | 45 | MIROC-ES2L | CULTIVATED | 316644 | 899 | 29055 | 70511 | 82.48 |
| 2070 | 45 | MIROC6 | CULTIVATED | 307082 | 533 | 38617 | 70877 | 78.36 |
| 2070 | 45 | MRI-ESM2-0 | CULTIVATED | 325354 | 1621 | 20345 | 69789 | 86.4 |
| 2070 | 85 | BCC-CSM2-MR | CULTIVATED | 322340 | 4060 | 23359 | 67350 | 83.09 |
| 2070 | 85 | CNRM-CM6-1 | CULTIVATED | 314161 | 2069 | 31538 | 69341 | 80.49 |
| 2070 | 85 | CNRM-ESM2-1 | CULTIVATED | 315389 | 2101 | 30310 | 69309 | 81.05 |
| 2070 | 85 | CanESM5 | CULTIVATED | 313883 | 2087 | 31816 | 69323 | 80.35 |
| 2070 | 85 | IPSL-CM6A-LR | CULTIVATED | 318010 | 3974 | 27689 | 67436 | 80.99 |
| 2070 | 85 | MIROC-ES2L | CULTIVATED | 307568 | 1270 | 38131 | 70140 | 78.07 |
| 2070 | 85 | MIROC6 | CULTIVATED | 302725 | 1490 | 42974 | 69920 | 75.87 |
| 2070 | 85 | MRI-ESM2-0 | CULTIVATED | 312780 | 477 | 32919 | 70933 | 80.95 |
| 2090 | 45 | BCC-CSM2-MR | CULTIVATED | 323078 | 2342 | 22621 | 69068 | 84.69 |
| 2090 | 45 | CNRM-CM6-1 | CULTIVATED | 321075 | 1619 | 24624 | 69791 | 84.17 |
| 2090 | 45 | CNRM-ESM2-1 | CULTIVATED | 313493 | 940 | 32206 | 70470 | 80.96 |
| 2090 | 45 | CanESM5 | CULTIVATED | 315003 | 716 | 30696 | 70694 | 81.82 |
| 2090 | 45 | IPSL-CM6A-LR | CULTIVATED | 319853 | 1653 | 25846 | 69757 | 83.53 |
| 2090 | 45 | MIROC-ES2L | CULTIVATED | 316648 | 1325 | 29051 | 70085 | 82.19 |
| 2090 | 45 | MIROC6 | CULTIVATED | 309379 | 391 | 36320 | 71019 | 79.46 |
| 2090 | 45 | MRI-ESM2-0 | CULTIVATED | 322144 | 2372 | 23555 | 69038 | 84.19 |
| 2090 | 85 | BCC-CSM2-MR | CULTIVATED | 319898 | 5030 | 25801 | 66380 | 81.15 |
| 2090 | 85 | CNRM-CM6-1 | CULTIVATED | 313307 | 4008 | 32392 | 67402 | 78.74 |
| 2090 | 85 | CNRM-ESM2-1 | CULTIVATED | 313434 | 4056 | 32265 | 67354 | 78.76 |
| 2090 | 85 | CanESM5 | CULTIVATED | 313381 | 4575 | 32318 | 66835 | 78.37 |
| 2090 | 85 | IPSL-CM6A-LR | CULTIVATED | 316801 | 6335 | 28898 | 65075 | 78.7 |
| 2090 | 85 | MIROC-ES2L | CULTIVATED | 304764 | 1322 | 40935 | 70088 | 76.84 |
| 2090 | 85 | MIROC6 | CULTIVATED | 295694 | 1440 | 50005 | 69970 | 73.12 |
| 2090 | 85 | MRI-ESM2-0 | CULTIVATED | 308178 | 1772 | 37521 | 69638 | 78 |
| 2050 | 45 | BCC-CSM2-MR | WILDsl | 326345 | 1023 | 17400 | 72341 | 88.7 |
| 2050 | 45 | CNRM-CM6-1 | WILDsl | 325472 | 1446 | 18273 | 71918 | 87.94 |
| 2050 | 45 | CNRM-ESM2-1 | WILDsl | 326017 | 2504 | 17728 | 70860 | 87.51 |
| 2050 | 45 | CanESM5 | WILDsl | 309819 | 1862 | 33926 | 71502 | 79.98 |
| 2050 | 45 | IPSL-CM6A-LR | WILDsl | 323599 | 3911 | 20146 | 69453 | 85.24 |
| 2050 | 45 | MIROC-ES2L | WILDsl | 325969 | 3517 | 17776 | 69847 | 86.77 |
| 2050 | 45 | MIROC6 | WILDsl | 324228 | 3327 | 19517 | 70037 | 85.98 |
| 2050 | 45 | MRI-ESM2-0 | WILDsl | 318885 | 5060 | 24860 | 68304 | 82.03 |
| 2050 | 85 | BCC-CSM2-MR | WILDsl | 321613 | 270 | 22132 | 73094 | 86.71 |
| 2050 | 85 | CNRM-CM6-1 | WILDsl | 324406 | 1807 | 19339 | 71557 | 87.13 |
| 2050 | 85 | CNRM-ESM2-1 | WILDsl | 324470 | 2070 | 19275 | 71294 | 86.98 |
| 2050 | 85 | CanESM5 | WILDsl | 303764 | 2397 | 39981 | 70967 | 77.01 |
| 2050 | 85 | IPSL-CM6A-LR | WILDsl | 319162 | 3260 | 24583 | 70104 | 83.43 |
| 2050 | 85 | MIROC-ES2L | WILDsl | 322684 | 4509 | 21061 | 68855 | 84.34 |
| 2050 | 85 | MIROC6 | WILDsl | 320659 | 5210 | 23086 | 68154 | 82.81 |
| 2050 | 85 | MRI-ESM2-0 | WILDsl | 322619 | 3464 | 21126 | 69900 | 85.04 |
| 2070 | 45 | BCC-CSM2-MR | WILDsl | 322962 | 1724 | 20783 | 71640 | 86.42 |
| 2070 | 45 | CNRM-CM6-1 | WILDsl | 324599 | 2760 | 19146 | 70604 | 86.57 |
| 2070 | 45 | CNRM-ESM2-1 | WILDsl | 320975 | 2893 | 22770 | 70471 | 84.6 |
| 2070 | 45 | CanESM5 | WILDsl | 305232 | 2377 | 38513 | 70987 | 77.64 |
| 2070 | 45 | IPSL-CM6A-LR | WILDsl | 321341 | 2604 | 22404 | 70760 | 84.98 |
| 2070 | 45 | MIROC-ES2L | WILDsl | 323422 | 4940 | 20323 | 68424 | 84.42 |
| 2070 | 45 | MIROC6 | WILDsl | 319352 | 6032 | 24393 | 67332 | 81.57 |
| 2070 | 45 | MRI-ESM2-0 | WILDsl | 317757 | 2572 | 25988 | 70792 | 83.21 |
| 2070 | 85 | BCC-CSM2-MR | WILDsl | 309566 | 1201 | 34179 | 72163 | 80.31 |
| 2070 | 85 | CNRM-CM6-1 | WILDsl | 318056 | 2664 | 25689 | 70700 | 83.3 |
| 2070 | 85 | CNRM-ESM2-1 | WILDsl | 316335 | 2673 | 27410 | 70691 | 82.46 |
| 2070 | 85 | CanESM5 | WILDsl | 292203 | 3371 | 51542 | 69993 | 71.82 |
| 2070 | 85 | IPSL-CM6A-LR | WILDsl | 310306 | 3593 | 33439 | 69771 | 79.03 |
| 2070 | 85 | MIROC-ES2L | WILDsl | 312297 | 7935 | 31448 | 65429 | 76.87 |
| 2070 | 85 | MIROC6 | WILDsl | 307203 | 6499 | 36542 | 66865 | 75.65 |
| 2070 | 85 | MRI-ESM2-0 | WILDsl | 309764 | 4848 | 33981 | 68516 | 77.92 |
| 2090 | 45 | BCC-CSM2-MR | WILDsl | 321087 | 1511 | 22658 | 71853 | 85.6 |
| 2090 | 45 | CNRM-CM6-1 | WILDsl | 322080 | 2860 | 21665 | 70504 | 85.18 |
| 2090 | 45 | CNRM-ESM2-1 | WILDsl | 318714 | 2704 | 25031 | 70660 | 83.59 |
| 2090 | 45 | CanESM5 | WILDsl | 303451 | 2188 | 40294 | 71176 | 77.02 |
| 2090 | 45 | IPSL-CM6A-LR | WILDsl | 315461 | 2735 | 28284 | 70629 | 81.99 |
| 2090 | 45 | MIROC-ES2L | WILDsl | 323595 | 5656 | 20150 | 67708 | 83.99 |
| 2090 | 45 | MIROC6 | WILDsl | 317802 | 3767 | 25943 | 69597 | 82.41 |
| 2090 | 45 | MRI-ESM2-0 | WILDsl | 318249 | 3392 | 25496 | 69972 | 82.89 |
| 2090 | 85 | BCC-CSM2-MR | WILDsl | 305489 | 1573 | 38256 | 71791 | 78.28 |
| 2090 | 85 | CNRM-CM6-1 | WILDsl | 311270 | 4073 | 32475 | 69291 | 79.13 |
| 2090 | 85 | CNRM-ESM2-1 | WILDsl | 311251 | 3603 | 32494 | 69761 | 79.45 |
| 2090 | 85 | CanESM5 | WILDsl | 285499 | 5298 | 58246 | 68066 | 68.18 |
| 2090 | 85 | IPSL-CM6A-LR | WILDsl | 306255 | 5458 | 37490 | 67906 | 75.97 |
| 2090 | 85 | MIROC-ES2L | WILDsl | 298376 | 5555 | 45369 | 67809 | 72.7 |
| 2090 | 85 | MIROC6 | WILDsl | 290236 | 8022 | 53509 | 65342 | 67.99 |
| 2090 | 85 | MRI-ESM2-0 | WILDsl | 303956 | 5963 | 39789 | 67401 | 74.66 |
|  |  |  |  |  |  |  |  |  |
| **GCM median** | | |  |  |  |  |  |  |
| year | ssp | GCM | Dom | none | pixLost | pixNew | pixKeep | percent |
| 2050 | 45 | Median | SEMIWILD | 329674 | 1227 | 15292 | 70916 | 89.57 |
| 2050 | 85 | Median | SEMIWILD | 328349 | 980 | 16617 | 71163 | 89 |
| 2070 | 45 | Median | SEMIWILD | 327492 | 1241 | 17474 | 70902 | 88.34 |
| 2070 | 85 | Median | SEMIWILD | 320410 | 799 | 24556 | 71344 | 84.91 |
| 2090 | 45 | Median | SEMIWILD | 327123 | 874 | 17843 | 71269 | 88.39 |
| 2090 | 85 | Median | SEMIWILD | 313900 | 1166 | 31066 | 70977 | 81.5 |
| 2050 | 45 | Median | COMMERCIAL | 329286 | 372 | 22455 | 64996 | 85.06 |
| 2050 | 85 | Median | COMMERCIAL | 328719 | 1121 | 23022 | 64247 | 84.18 |
| 2070 | 45 | Median | COMMERCIAL | 328327 | 1325 | 23414 | 64043 | 83.81 |
| 2070 | 85 | Median | COMMERCIAL | 324787 | 2259 | 26954 | 63109 | 81.21 |
| 2090 | 45 | Median | COMMERCIAL | 327051 | 1397 | 24690 | 63971 | 83.06 |
| 2090 | 85 | Median | COMMERCIAL | 323286 | 2869 | 28455 | 62499 | 79.96 |
| 2050 | 45 | Median | LANDRACE | 357115 | 1055 | 12898 | 46041 | 86.84 |
| 2050 | 85 | Median | LANDRACE | 356499 | 1315 | 13514 | 45781 | 86.06 |
| 2070 | 45 | Median | LANDRACE | 355963 | 1160 | 14050 | 45936 | 85.8 |
| 2070 | 85 | Median | LANDRACE | 353484 | 1122 | 16529 | 45974 | 83.89 |
| 2090 | 45 | Median | LANDRACE | 356260 | 1135 | 13753 | 45961 | 86.06 |
| 2090 | 85 | Median | LANDRACE | 352102 | 1924 | 17911 | 45172 | 82 |
| 2050 | 45 | Median | WILD | 324800 | 1398 | 19078 | 71833 | 87.53 |
| 2050 | 85 | Median | WILD | 323222 | 1421 | 20656 | 71810 | 86.68 |
| 2070 | 45 | Median | WILD | 322962 | 1978 | 20916 | 71253 | 86.16 |
| 2070 | 85 | Median | WILD | 314002 | 1825 | 29876 | 71406 | 81.83 |
| 2090 | 45 | Median | WILD | 321168 | 1639 | 22710 | 71592 | 85.47 |
| 2090 | 85 | Median | WILD | 304283 | 2065 | 39595 | 71166 | 77.36 |
| 2050 | 45 | Median | CULTIVATED | 320583 | 125 | 25116 | 71285 | 84.96 |
| 2050 | 85 | Median | CULTIVATED | 319648 | 489 | 26051 | 70921 | 84.24 |
| 2070 | 45 | Median | CULTIVATED | 319182 | 709 | 26517 | 70701 | 83.85 |
| 2070 | 85 | Median | CULTIVATED | 315294 | 1468 | 30405 | 69942 | 81.44 |
| 2090 | 45 | Median | CULTIVATED | 318725 | 754 | 26974 | 70656 | 83.6 |
| 2090 | 85 | Median | CULTIVATED | 312817 | 2304 | 32882 | 69106 | 79.71 |
| 2050 | 45 | Median | WILDsl | 324102 | 1415 | 19643 | 71949 | 87.23 |
| 2050 | 85 | Median | WILDsl | 321692 | 1446 | 22053 | 71918 | 85.96 |
| 2070 | 45 | Median | WILDsl | 321947 | 2007 | 21798 | 71357 | 85.7 |
| 2070 | 85 | Median | WILDsl | 312706 | 2086 | 31039 | 71278 | 81.14 |
| 2090 | 45 | Median | WILDsl | 319582 | 1716 | 24163 | 71648 | 84.7 |
| 2090 | 85 | Median | WILDsl | 306030 | 2500 | 37715 | 70864 | 77.9 |
|  |  |  |  |  |  |  |  |  |
| **GCM Intersecion** | | |  |  |  |  |  |  |
| year | ssp | GCM | Dom | none | pixLost | pixNew | pixKeep | percent |
| 2050 | 45 | Interseciton | SEMIWILD | 338278 | 9078 | 6688 | 63065 | 88.89 |
| 2050 | 85 | Interseciton | SEMIWILD | 337680 | 11017 | 7286 | 61126 | 86.98 |
| 2070 | 45 | Interseciton | SEMIWILD | 337511 | 10449 | 7455 | 61694 | 87.33 |
| 2070 | 85 | Interseciton | SEMIWILD | 335865 | 13431 | 9101 | 58712 | 83.9 |
| 2090 | 45 | Interseciton | SEMIWILD | 337137 | 9721 | 7829 | 62422 | 87.68 |
| 2090 | 85 | Interseciton | SEMIWILD | 334195 | 13425 | 10771 | 58718 | 82.92 |
| 2050 | 45 | Interseciton | COMMERCIAL | 342337 | 3289 | 9404 | 62079 | 90.72 |
| 2050 | 85 | Interseciton | COMMERCIAL | 342483 | 4632 | 9258 | 60736 | 89.74 |
| 2070 | 45 | Interseciton | COMMERCIAL | 341895 | 4878 | 9846 | 60490 | 89.15 |
| 2070 | 85 | Interseciton | COMMERCIAL | 340523 | 7284 | 11218 | 58084 | 86.26 |
| 2090 | 45 | Interseciton | COMMERCIAL | 341923 | 5817 | 9818 | 59551 | 88.4 |
| 2090 | 85 | Interseciton | COMMERCIAL | 339595 | 10419 | 12146 | 54949 | 82.97 |
| 2050 | 45 | Interseciton | LANDRACE | 365736 | 9403 | 4277 | 37693 | 84.64 |
| 2050 | 85 | Interseciton | LANDRACE | 365240 | 10131 | 4773 | 36965 | 83.22 |
| 2070 | 45 | Interseciton | LANDRACE | 365883 | 11071 | 4130 | 36025 | 82.58 |
| 2070 | 85 | Interseciton | LANDRACE | 365415 | 12689 | 4598 | 34407 | 79.92 |
| 2090 | 45 | Interseciton | LANDRACE | 365423 | 9684 | 4590 | 37412 | 83.98 |
| 2090 | 85 | Interseciton | LANDRACE | 364449 | 16887 | 5564 | 30209 | 72.91 |
| 2050 | 45 | Interseciton | WILD | 338013 | 8275 | 5865 | 64956 | 90.18 |
| 2050 | 85 | Interseciton | WILD | 337440 | 9587 | 6438 | 63644 | 88.82 |
| 2070 | 45 | Interseciton | WILD | 338179 | 9906 | 5699 | 63325 | 89.03 |
| 2070 | 85 | Interseciton | WILD | 335206 | 13760 | 8672 | 59471 | 84.13 |
| 2090 | 45 | Interseciton | WILD | 337170 | 9844 | 6708 | 63387 | 88.45 |
| 2090 | 85 | Interseciton | WILD | 333646 | 18814 | 10232 | 54417 | 78.93 |
| 2050 | 45 | Interseciton | CULTIVATED | 334765 | 2948 | 10934 | 68462 | 90.79 |
| 2050 | 85 | Interseciton | CULTIVATED | 335648 | 4384 | 10051 | 67026 | 90.28 |
| 2070 | 45 | Interseciton | CULTIVATED | 334878 | 5027 | 10821 | 66383 | 89.34 |
| 2070 | 85 | Interseciton | CULTIVATED | 334290 | 7121 | 11409 | 64289 | 87.4 |
| 2090 | 45 | Interseciton | CULTIVATED | 334024 | 5237 | 11675 | 66173 | 88.67 |
| 2090 | 85 | Interseciton | CULTIVATED | 333070 | 10247 | 12629 | 61163 | 84.25 |
| 2050 | 45 | Interseciton | WILDsl | 336917 | 8926 | 6828 | 64438 | 89.11 |
| 2050 | 85 | Interseciton | WILDsl | 336763 | 9520 | 6982 | 63844 | 88.56 |
| 2070 | 45 | Interseciton | WILDsl | 337202 | 10569 | 6543 | 62795 | 88.01 |
| 2070 | 85 | Interseciton | WILDsl | 334754 | 13749 | 8991 | 59615 | 83.98 |
| 2090 | 45 | Interseciton | WILDsl | 335949 | 9930 | 7796 | 63434 | 87.74 |
| 2090 | 85 | Interseciton | WILDsl | 333484 | 17329 | 10261 | 56035 | 80.24 |
