# Supplementary material for: Fluctuation of ecological niches and geographic range shifts along chile pepper's domestication gradient
Source: Ecol Evol. 2023 Nov 28;13(11):e10731. doi: 10.1002/ece3.10731 (PMC10682905; doi:10.1002/ece3.10731)
Supplement: Supplementary file 1 — Appendix S1 [file ECE3-13-e10731-s001.zip › SuppTable_S8.docx]

**Supplementary table S8**

|  |  |  | 2050 | | 2070 | | 2090 | |
| --- | --- | --- | --- | --- | --- | --- | --- | --- |
| State | Landrace name | Cultivation type* | SSP 2_45 | SSP 5_85 | SSP 2_45 | SSP 5_85 | SSP 2_45 | SSP 5_85 |
| Campeche | MAAX IK | B | - | - | - | - | - | at risk |
| Campeche | TABAQUERO | M | - | at risk | at risk | at risk | - | at risk |
| Chiapas | CHILE DE ÁRBOL | B | - | - | - | at risk | - | at risk |
| Chiapas | CHILITO | NA | - | - | - | at risk | - | - |
| Chiapas | MIRAPARRIBA | B | - | - | - | at risk | - | at risk |
| Chiapas | MIRAPARRIBA | B | - | - | - | at risk | - | at risk |
| Oaxaca | BOLITA | B | - | - | - | at risk | - | at risk |
| Oaxaca | CHILE PARADITO | B | - | - | - | - | - | at risk |
| Oaxaca | CHILGOLE | B | - | - | - | at risk | - | at risk |
| Oaxaca | COSTEÑO ROJO | P | - | at risk | - | at risk | at risk | at risk |
| Oaxaca | DE AGUA | P | - | - | - | at risk | - | - |
| Oaxaca | GUAJILLO | B | - | - | - | at risk | - | at risk |
| Oaxaca | HUACLE | M | - | - | - | - | - | at risk |
| Oaxaca | JALAPEÑO | B | - | - | - | at risk | - | at risk |
| Oaxaca | MIRASOL-TRAS | B | - | - | - | at risk | - | at risk |
| Oaxaca | PIJITA | B | - | - | - | at risk | - | at risk |
| Oaxaca | TAVICHE | M | - | - | - | at risk | - | - |
| Oaxaca | TUSTA | B | - | - | - | - | - | at risk |
| Oaxaca | TUSTA | B | - | - | - | at risk | - | at risk |
| Oaxaca | TUSTA | M | - | - | - | at risk | - | at risk |
| Oaxaca | TUSTA | B | - | - | - | - | - | at risk |
| Oaxaca | TUSTA | M | - | - | - | - | - | at risk |
| Oaxaca | TUSTA | M | - | - | - | at risk | - | at risk |
| Oaxaca | TUSTA | NA | - | - | - | - | - | at risk |
| Oaxaca | TUSTA | NA | - | - | - | at risk | - | at risk |
| Querétaro | CHILE CRIOLLO | B | - | - | - | at risk | at risk | at risk |
| Querétaro | CHILE CRIOLLO | B | - | at risk | - | at risk | at risk | at risk |
| Tabasco | GARBANZO | F | - | at risk | - | - | at risk | - |
| Yucatán | CHILE DULCE | M | - | - | - | at risk | - | at risk |
| Yucatán | CHILE DULCE | M | - | - | - | - | - | at risk |
| Yucatán | XCATIC | M | - | - | - | - | - | at risk |
|  |  |  |  |  |  |  |  |  |
| (*) Cultivation types: F=forest, B=backyard, M=milpa, P=plantation | | | | |  |  |  |  |
