# Supplementary figures and images for: Fluctuation of ecological niches and geographic range shifts along chile pepper's domestication gradient
Source: Ecol Evol. 2023 Nov 28;13(11):e10731. doi: 10.1002/ece3.10731 (PMC10682905; doi:10.1002/ece3.10731)

wild

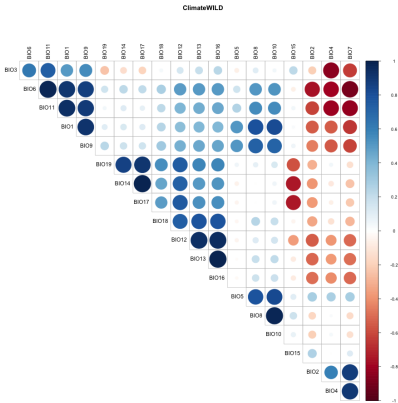

wild sl

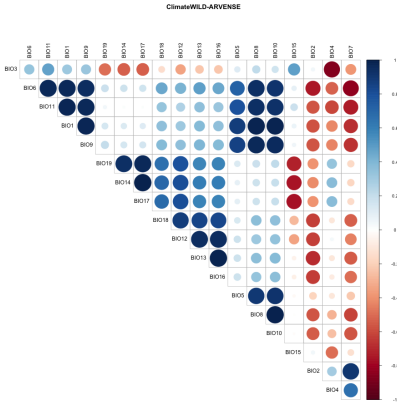

semiwild

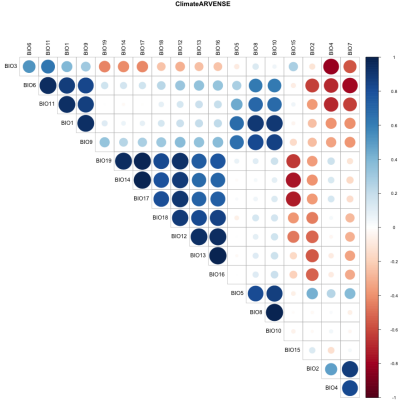

ALL

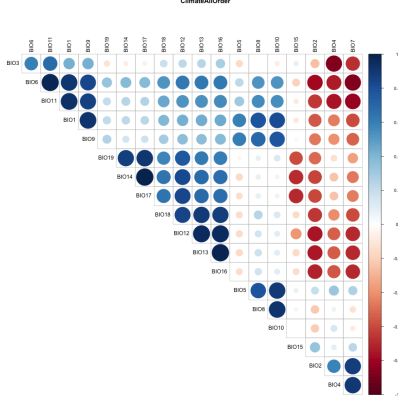

landrace

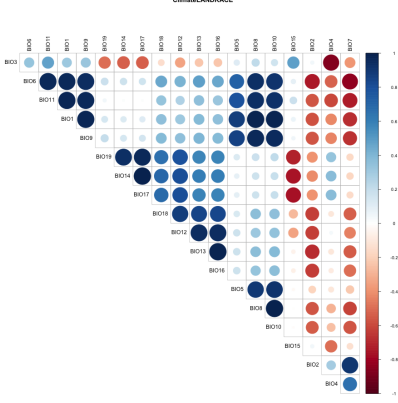

cultivated

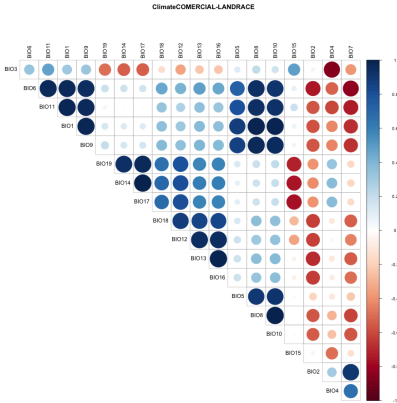

commercial

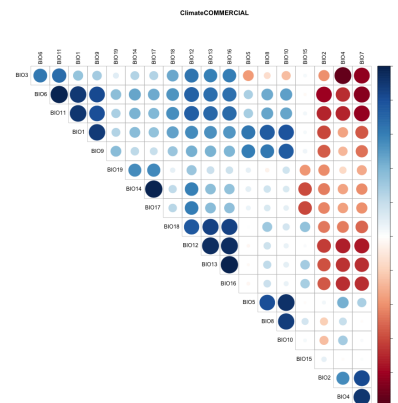

Supplement: Supplementary file 1 — Appendix S1 [file ECE3-13-e10731-s001.zip › Appendix1_SuppFig_SA2.pdf]

Appendix1 Supp. figure SA3

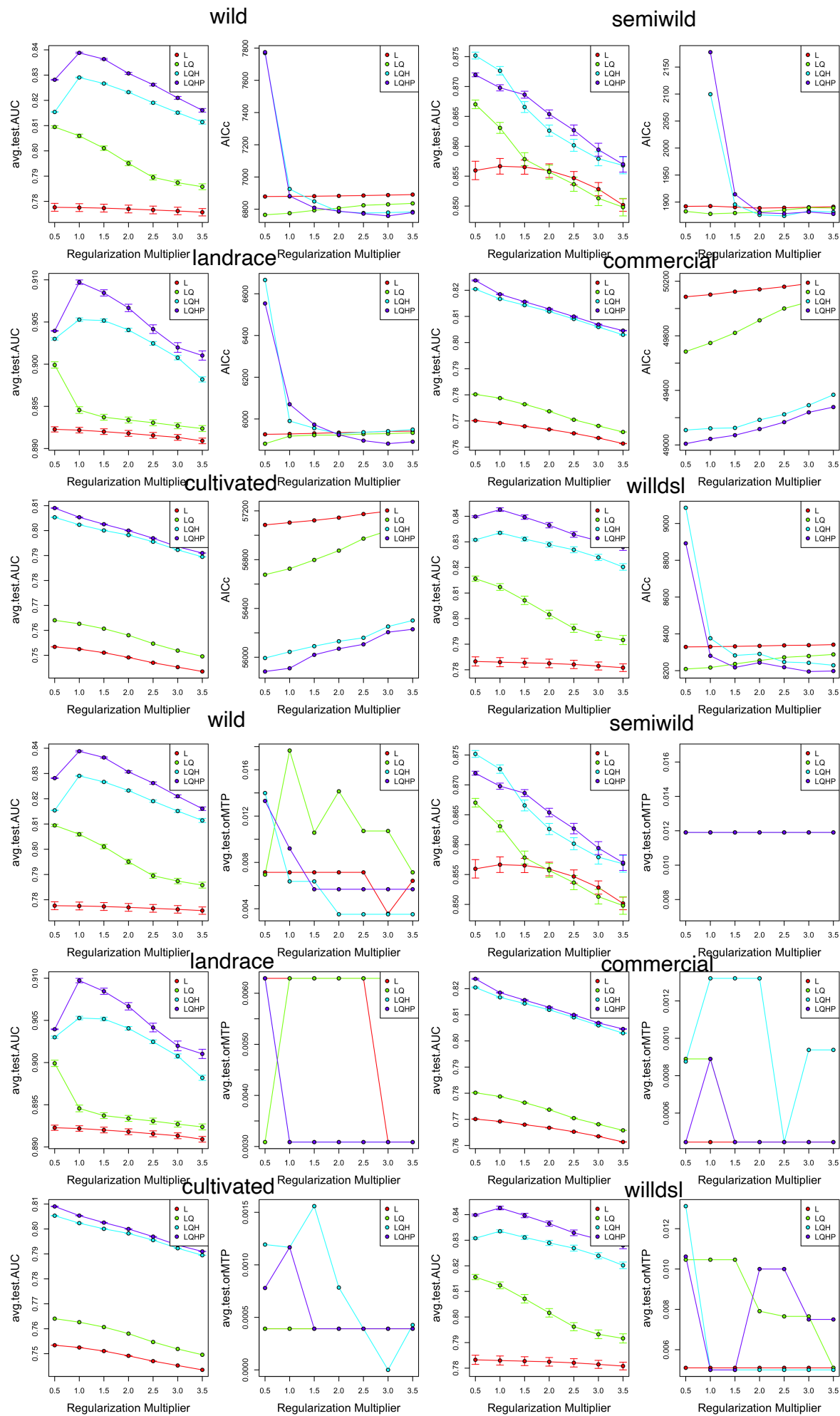

Supplement: Supplementary file 1 — Appendix S1 [file ECE3-13-e10731-s001.zip › Appendix1_SuppFig_SA3.pdf]

Supp. figure S1

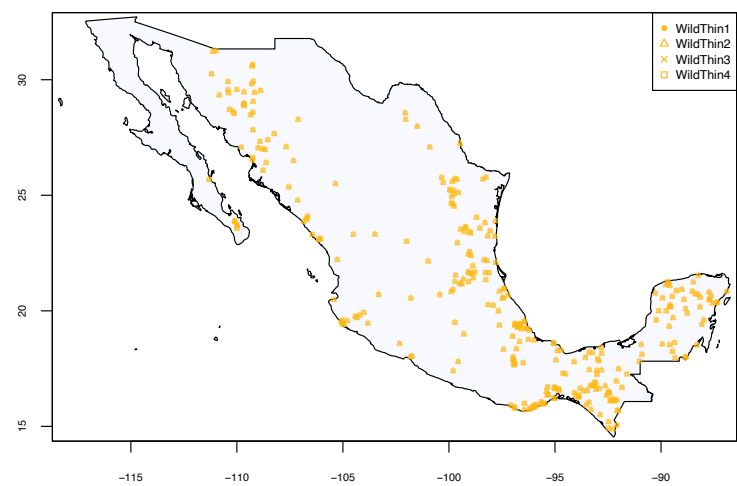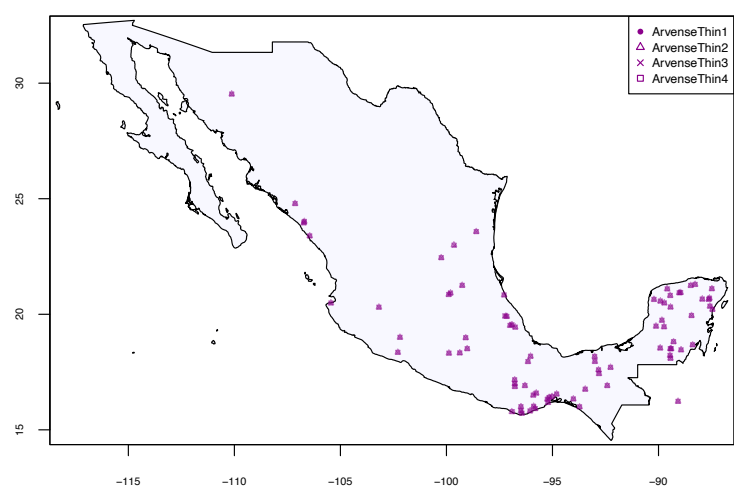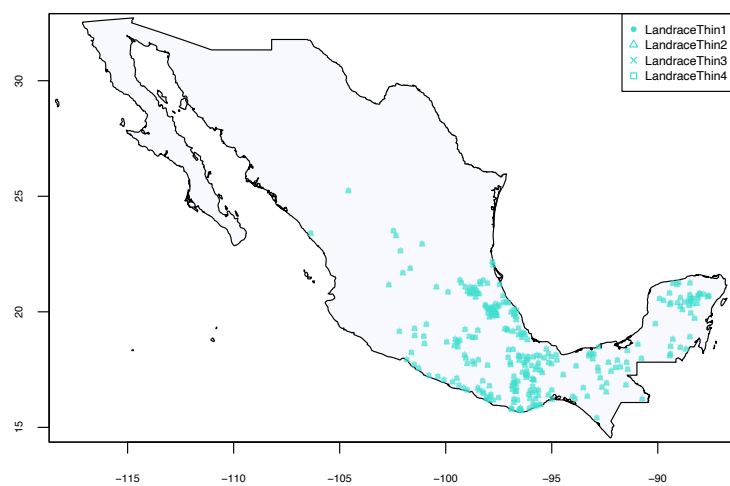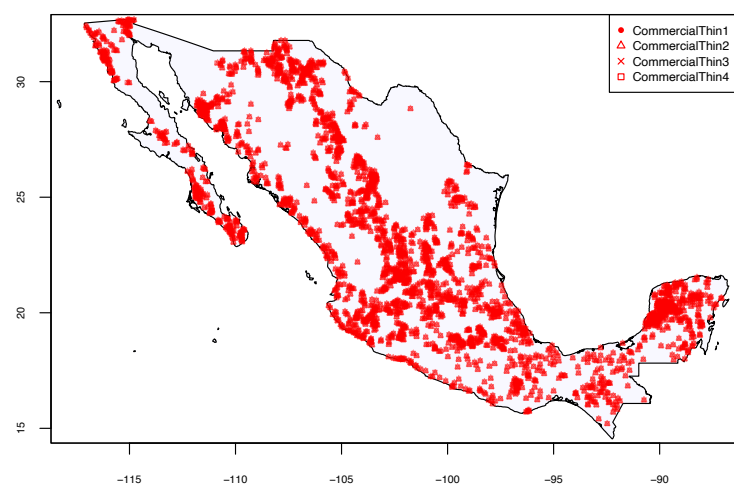

Supplement: Supplementary file 1 — Appendix S1 [file ECE3-13-e10731-s001.zip › SuppFig_S1.pdf]

LANDRACES

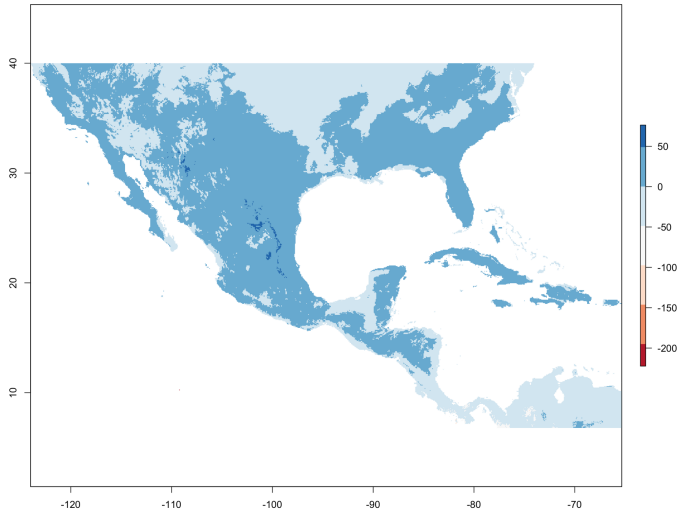

MRI-ESM2-0

2090 SSP=2\_45

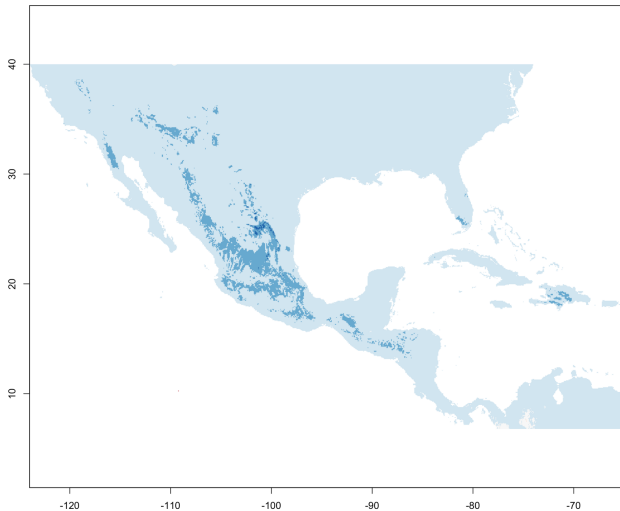

IPSL-CM6A-LR

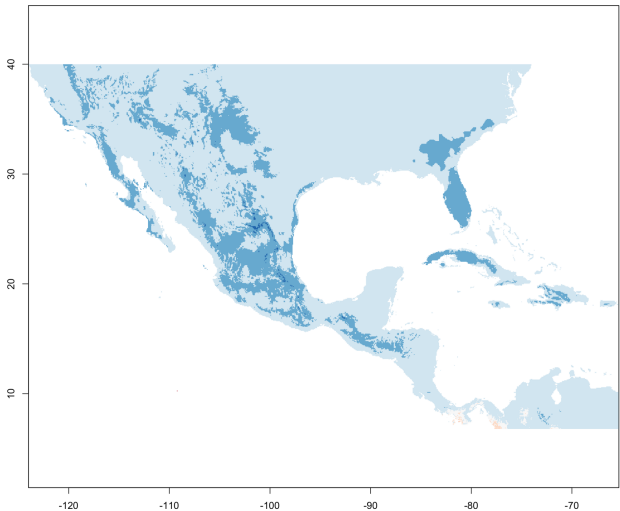

MRI-ESM2-0

2090 SSP=5\_85

Supplement: Supplementary file 1 — Appendix S1 [file ECE3-13-e10731-s001.zip › SuppFig_S10.pdf]

## Supp. figure S2

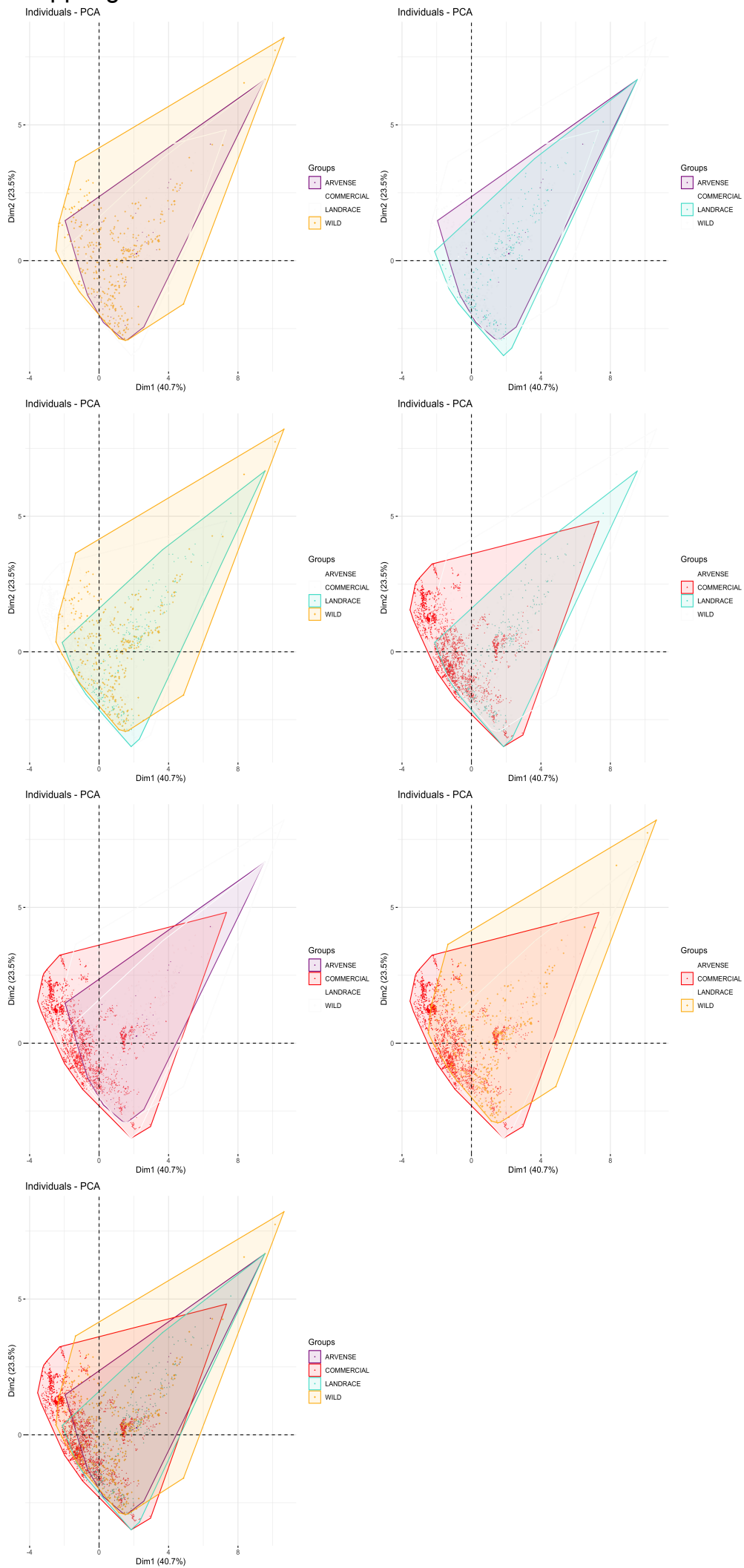

Supplement: Supplementary file 1 — Appendix S1 [file ECE3-13-e10731-s001.zip › SuppFig_S2.pdf]

Supp. figure S3

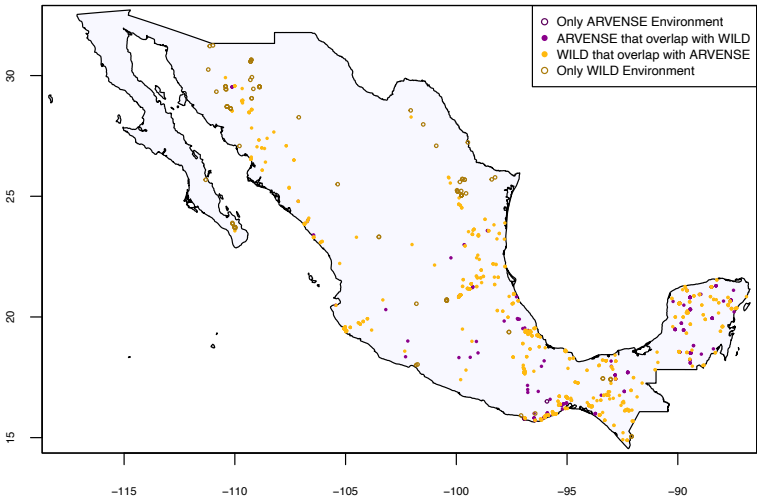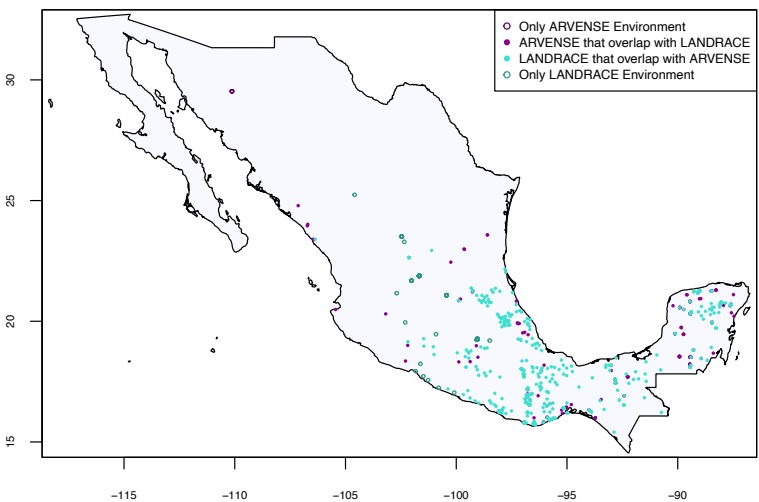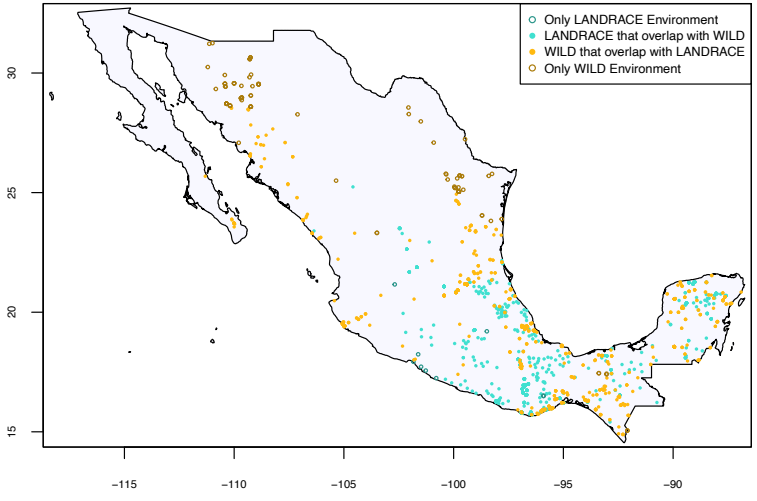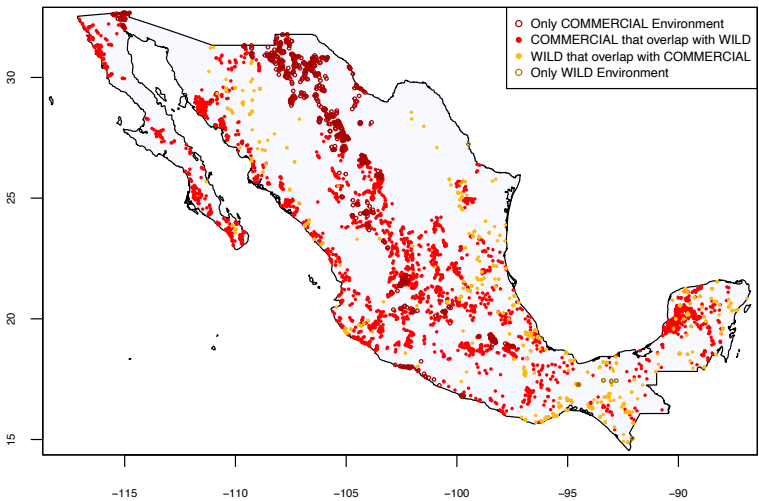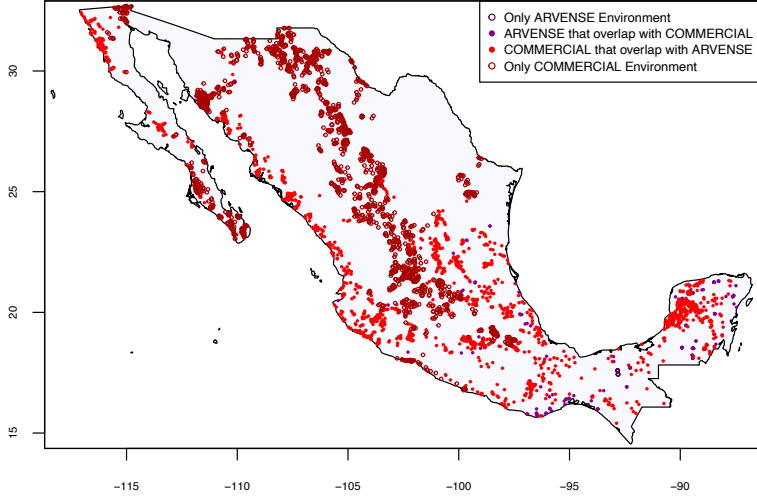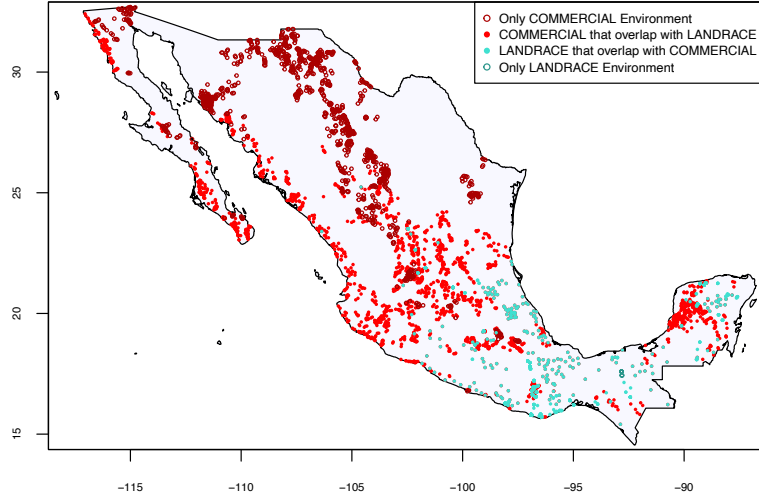

Supplement: Supplementary file 1 — Appendix S1 [file ECE3-13-e10731-s001.zip › SuppFig_S3.pdf]

Supp. figure S6

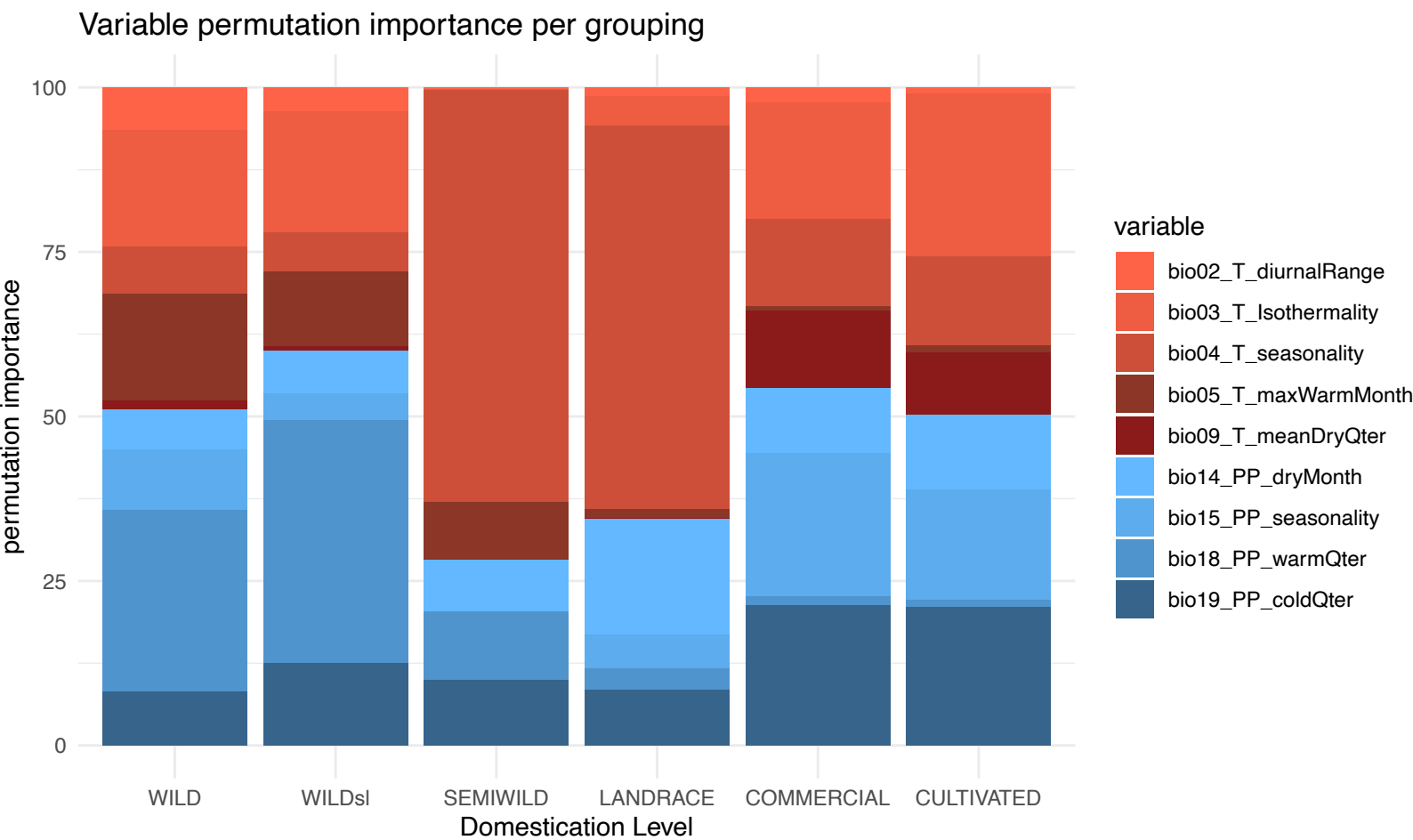

Supplement: Supplementary file 1 — Appendix S1 [file ECE3-13-e10731-s001.zip › SuppFig_S6.pdf]

Supp. figure S7

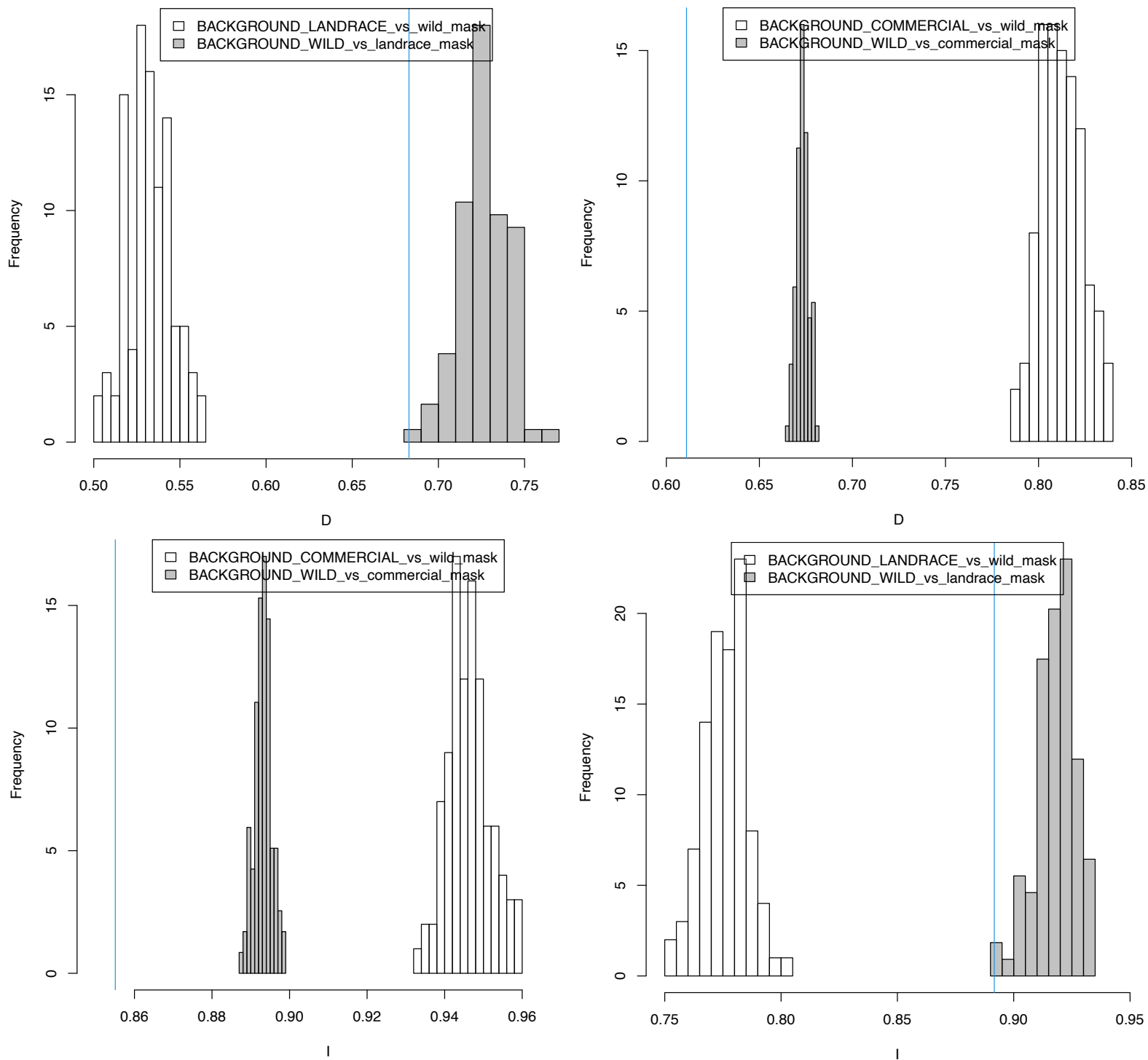

Supplement: Supplementary file 1 — Appendix S1 [file ECE3-13-e10731-s001.zip › SuppFig_S7.pdf]

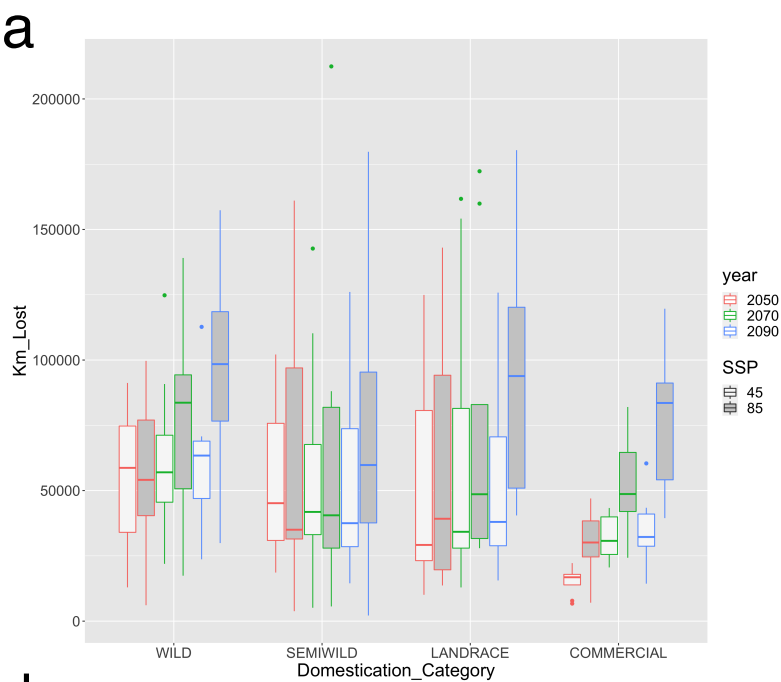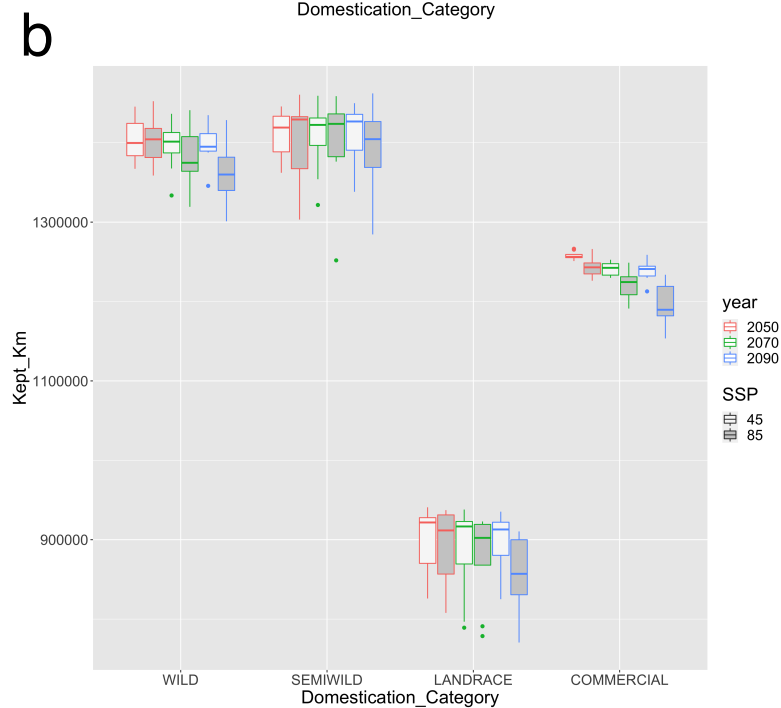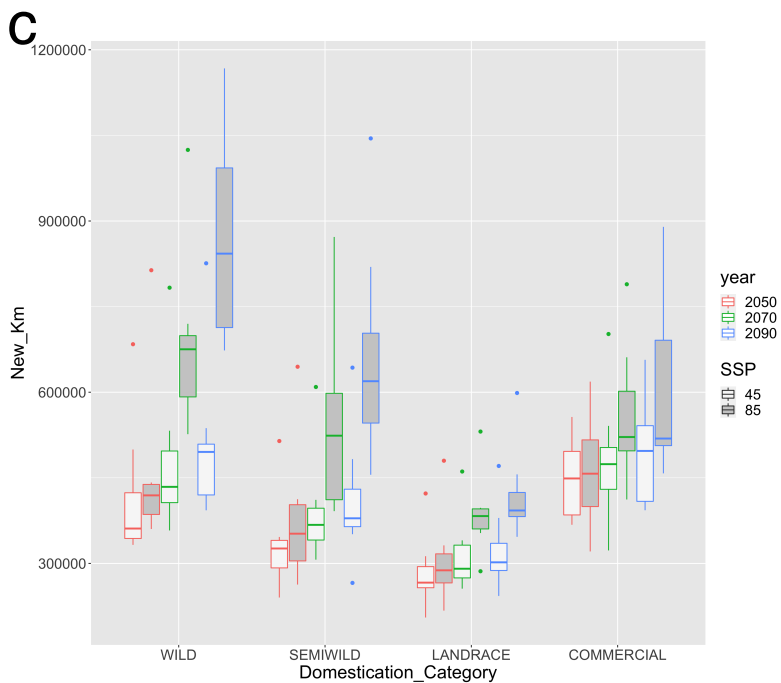

Supplement: Supplementary file 1 — Appendix S1 [file ECE3-13-e10731-s001.zip › SuppFig_S8.pdf]
